# UFL1‐Mediated UFMylation of ENO1 Restrains Aerobic Glycolysis and Colorectal Cancer Progression

**DOI:** 10.1002/advs.76875

**Published:** 2026-07-29

**Authors:** Xiuqing Ma, Rui Wan, Yueyuan Zhong, Ziyang Cui, Xuan Zhang, Xiaoqiang He, Rong Wang, Lei Huang, Wan‐Yang Sun, Rong‐Rong He, Yang Zhou, Jianshuang Li, Li Shen, Shao‐Hua Wang, Tongzheng Liu

**Affiliations:** ^1^ State Key Laboratory of Bioactive Molecules and Druggability Assessment Guangdong Basic Research Center of Excellence for Natural Bioactive Molecules and Discovery of Innovative Drugs College of Pharmacy Jinan University Guangzhou China; ^2^ Department of Dermatology and Venereology Peking University First Hospital Peking University Beijing China; ^3^ School of Pharmacy Lanzhou University Lanzhou China; ^4^ Guangdong Engineering Research Center of Chinese Medicine and Disease Susceptibility College of Pharmacy Jinan University Guangzhou China; ^5^ College of Life Science and Technology Jinan University Guangzhou Guangdong China; ^6^ Guangdong Provincial Engineering Technology Research Center for Innovative Drugs and Bioproducts School of Pharmacy Guangdong Medical University Dongguan Guangdong China

**Keywords:** aerobic glycolysis, colorectal cancer, ENO1, UFL1, UFMylation

## Abstract

Metabolic reprogramming toward enhanced aerobic glycolysis is a hallmark of cancer, yet the contribution of ubiquitin‐like modifications to this process remains poorly understood. Here, we identify the UFMylation E3 ligase UFL1 as a critical suppressor of glycolytic metabolism and colorectal cancer (CRC) progression. Mechanistically, UFL1 directly interacts with the glycolytic enzyme enolase 1 (ENO1) and catalyzes its UFMylation at lysine residues K285 and K420. This modification disrupts ENO1 dimerization, attenuates its enzymatic activity, and consequently suppresses glycolytic flux. Functionally, UFL1‐mediated UFMylation inhibits tumor growth both in vitro and in vivo. Notably, pharmacological enhancement of the UFL1‐ENO1 interaction using the FDA‐approved antibiotic torezolid significantly potentiates the antitumor efficacy of 5‐fluorouracil (5‐FU) across multiple preclinical models, including CRC patient‐derived organoids and xenografts, without detectable toxicity. Collectively, these findings identify ENO1 as a direct substrate of UFMylation, establish UFMylation as a previously unrecognized regulator of cancer metabolic reprogramming, and highlight the UFL1‐ENO1 axis as a promising therapeutic target for colorectal cancer.

## Graphical abstract

UFMylation restrains aerobic glycolysis and colorectal cancer progression by modifying the glycolytic enzyme ENO1. Pharmacological enhancement of the UFL1–ENO1 interaction by the FDA‐approved antibiotic torezolid promotes ENO1 UFMylation, suppresses tumor metabolism, and sensitizes colorectal cancer to chemotherapy, revealing an actionable metabolic vulnerability.

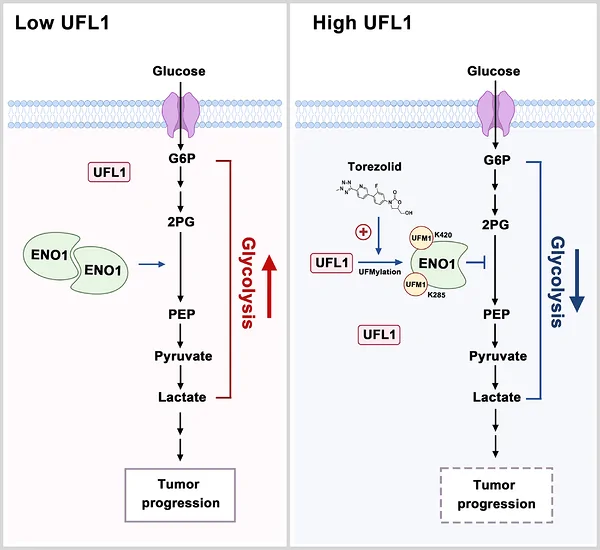

## 1 Introduction

Colorectal cancer (CRC) is one of the most prevalent malignancies and a leading cause of cancer‐related mortality worldwide [1]. Despite improvement in screening and therapeutic strategies, disease recurrence and drug resistance remain major clinical challenges [2]. A deeper understanding of the molecular mechanisms that drive CRC progression is therefore essential for the development of more effective treatments.

Metabolic reprogramming, particularly enhanced glycolysis even under normoxic conditions, a phenomenon known as the Warburg effect, is a hallmark of cancer [3]. This metabolic shift supports rapid proliferation by diverting glycolytic intermediates into anabolic pathways for nucleotide, amino acid, and lipid synthesis, thereby sustaining tumor growth and survival [4]. Oncogenic activation of KRAS, MYC, and PI3K/AKT further reinforces the Warburg effect by upregulating glucose transporters and key glycolytic enzymes [5]. Enolase 1 (ENO1), which catalyzes the conversion of 2‐phosphoglycerate to phosphoenolpyruvate, is frequently overexpressed and hyperactivated in multiple cancers, including CRC, where it enhances glycolytic flux and correlates with poor patient outcomes [6]. Consequently, inhibiting glycolysis has emerged as an attractive anticancer strategy. Although inhibitors targeting glycolytic enzymes and lactate metabolism have shown promise in preclinical models [7, 8], their clinical translation has been limited by suboptimal efficacy, systemic toxicity, metabolic plasticity, and incomplete understanding of upstream regulatory mechanisms governing glycolysis in cancer [7].

UFMylation is a dynamic and reversible post‐translational modification mediated by a three‐enzyme cascade comprising the E1 activating enzyme UBA5, the E2 conjugating enzyme UFC1, and the E3 ligase UFL1 [9]. De‐UFMylation is catalyzed by the cysteine proteases UFSP1 and UFSP2 [10]. Notably, UFSP2 functions as the principal de‐UFMylase in mammalian cells, whereas UFSP1 is expressed at substantially lower levels and primarily contributes to precursor UFM1 maturation [11, 12]. UFMylation regulates diverse biological processes, including endoplasmic reticulum (ER) homeostasis, proteostasis, DNA damage response and immune regulation [13, 14, 15, 16]. Despite these broad functional roles, whether UFMylation contributes to metabolic reprogramming in cancer remains largely unknown.

Here, we demonstrate that UFL1‐mediated UFMylation of ENO1 suppresses aerobic glycolysis and limits CRC growth. UFL1 interacts with and conjugates UFM1 to ENO1 at lysine residues K285 and K420, a modification that disrupts ENO1 dimerization and reduces its enzymatic activity. Furthermore, enhancing the UFL1‐ENO1 interaction using the FDA‐approved antibiotic torezolid potentiates the anti‐tumor efficacy of 5‐fluorouracil (5‐FU) across multiple preclinical models, including CRC patient‐derived organoids and xenografts. Collectively, these findings uncover a previously unrecognized regulatory layer controlling cancer metabolism and suggest that pharmacological modulation of the UFL1‐ENO1 axis represents a promising therapeutic strategy for CRC.

## 2 Results

### 2.1 UFL1 Suppresses Aerobic Glycolysis in Colorectal Cancer

To assess the clinical relevance of UFL1 in CRC, we first evaluated its expression in 60 primary CRC specimens by immunohistochemistry (IHC) and in 10 paired tumor and adjacent normal tissue samples by immunoblotting. UFL1 protein levels were markedly reduced in tumor tissues compared to adjacent normal tissues (Figure 1A–C). Furthermore, UFL1 expression levels progressively decreased with advancing clinicopathological stages in CRC patients (Table S1). Kaplan‐Meier analysis further showed that high UFL1 expression was associated with improved recurrence‐free survival (Figure S1A). To explore the functional significance of UFL1 in CRC, we analyzed single‐cell RNA‐sequencing (scRNA‐seq) data from 102 samples, including 79 CRC tumors and 23 healthy controls (Table S2). As shown in Figure 1D and Figure S1B, UFL1 expression exhibited a strong inverse correlation with the glycolysis score in malignant epithelial cells. This relationship was independently validated using an additional human CRC scRNA‐seq dataset (GEO: GSE132465) [17], in which UFL1 expression similarly showed a negative correlation with the glycolysis score across 23 CRC samples (Figure 1E).

**FIGURE 1:**
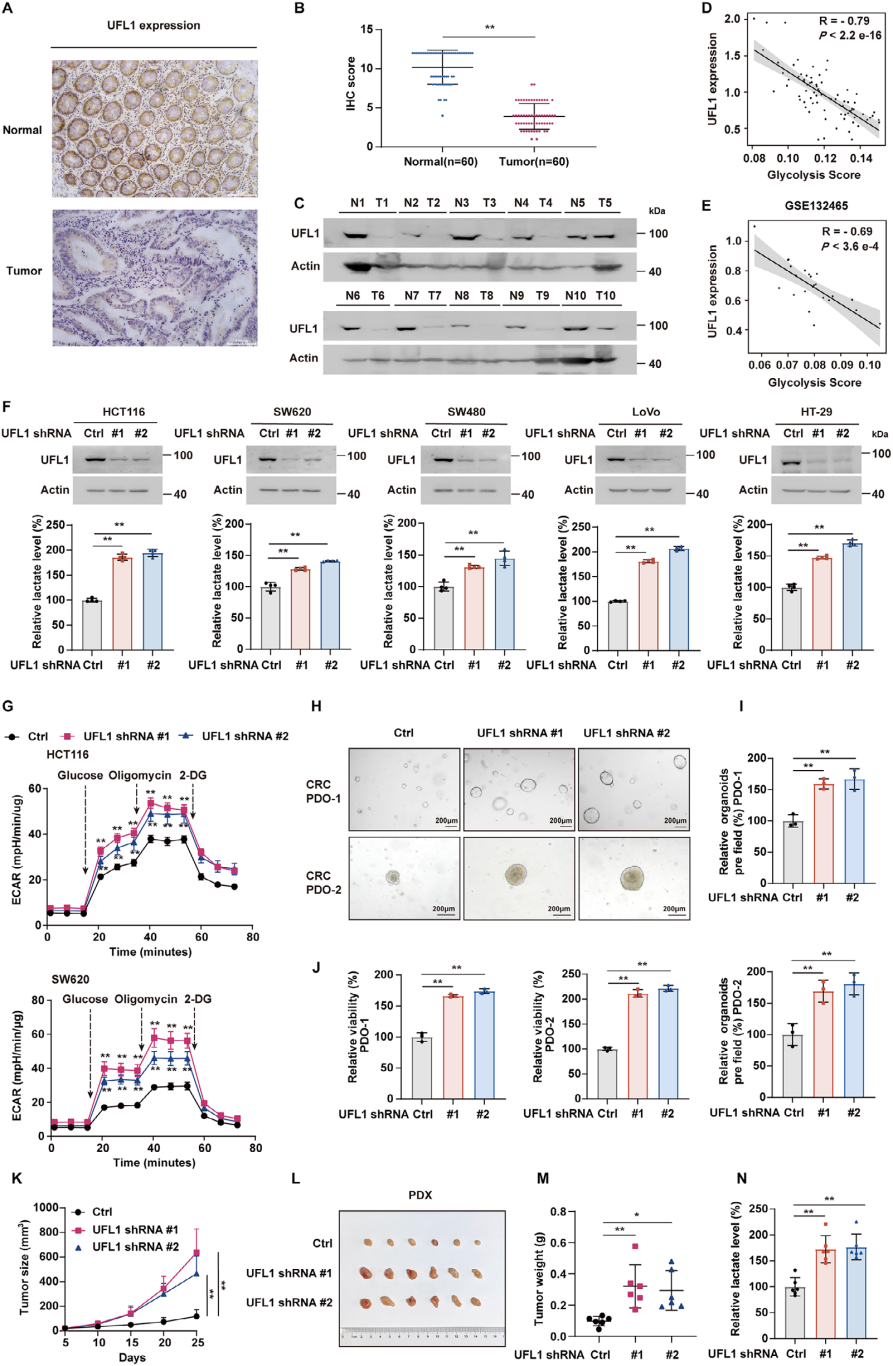
UFL1 suppresses aerobic glycolysis in colorectal cancer. (A, B) Representative immunohistochemical images of UFL1 in CRC patient samples (n = 60) and normal tissues (n = 60). Scale bar, 100 µm. The analysis of UFL1 IHC score was shown in right panel. (C) Western blotting analysis of UFL1 expression in 10 paired CRC specimens and adjacent normal tissues. N, adjacent normal tissue; T, tumor tissue. (D) Analysis of the correlation between UFL1 expression and glycolysis score in scRNA‐seq data. (E) Reanalysis of the correlation between UFL1 expression and glycolysis score in scRNA‐seq dataset (GSE132465). (F) Lactate production assays in various CRC cells stably expressing control (Ctrl), or UFL1 shRNAs (n = 4). (G) Seahorse assay assessing ECAR in HCT116 and SW620 cells stably expressing control (Ctrl), or UFL1 shRNAs (n = 4). (H) Representative images of CRC patient‐derived organoids stably expressing control (Ctrl), or UFL1 shRNAs. Scale bar, 200 µm. Upper row  =  CRC PDO‐1; lower row  =  CRC PDO‐2. (I) Quantification of organoids ≥100 µm in diameter per field (n = 3). (J) Cell viability assays were performed to assess the effect of UFL1 knockdown on CRC organoid viability (n = 3). (K‐M) Representative tumor growth curve from CRC patient‐derived xenografts (PDXs) in control and experimental groups(K). Analysis of tumor image (L) and tumor weight (M) (n = 6). (N) Tumors from PDX models were homogenized, and lactate levels were analyzed (n = 6). Error bars represent the SD. Statistical significance was determined using an unpaired Student's *t*‐test (B) or one‐way ANOVA followed by Tukey's multiple comparisons test (F, G, I, J, K, M, N); ^**^ *p* < 0.01, ^*^ *p* < 0.05. Raw microscopy images are available in Data S2. Raw blots are available in Data S3.

Based on these observations, we hypothesized that UFL1 suppresses aerobic glycolysis in CRC. Consistent with this hypothesis, UFL1 knockdown significantly increased lactate production in multiple CRC cell lines (Figure 1F), whereas UFL1 overexpression produced the opposite effect (Figure S1C). This metabolic regulation was independent of p53 status, as UFL1 depletion similarly enhanced lactate accumulation in p53‐/‐ HCT116 cells (Figure S1D). In line with these findings, basal extracellular acidification rates (ECARs) were markedly elevated upon UFL1 knockdown (Figure 1G and Figure S1E), indicating enhanced glycolytic activity.

Importantly, UFL1 deficiency promoted tumor growth and increased lactate production in patient‐derived models, including CRC‐derived organoids (PDOs) and xenografts (PDXs) (Figure 1H–N and Figure S1F, G). Given the dependence of CRC cells on glycolysis for proliferation [5], we next examined whether UFL1‐mediated regulation of glycolysis affects cell growth. As shown in Figure S1H, I, UFL1 overexpression significantly suppressed cell proliferation, and this effect was largely rescued by exogenous supplementation with pyruvate or lactate. Together, these results support a functional link between UFL1‐mediated suppression of glycolysis and inhibition of tumor cell growth.

### 2.2 UFL1 Directly Interacts With ENO1

To elucidate how UFL1 regulates glycolysis, we performed affinity purification of Flag‐tagged UFL1 followed by mass spectrometry analysis. In addition to known interactors such as histone H4 [15], CYB5R3 [18], RPL26 [13], and the core UFMylation machinery components UFC1 and UFBP1, we identified enolase 1 (ENO1), a glycolytic enzyme that converts 2‐phosphoglycerate (2‐PG) to phosphoenolpyruvate (PEP), as a putative UFL1‐binding protein (Figure 2A and Data S1). Co‐immunoprecipitation assays confirmed the interaction between UFL1 and ENO1 in HCT116 and SW620 cells (Figure 2B,C), and direct binding was further validated in vitro using purified GST‐ENO1 and His‐UFL1 (Figure 2D). To further substantiate the association between UFL1 and ENO1 in cells, we performed a TurboID‐based proximity labeling assay followed by streptavidin pulldown. ENO1 was robustly enriched in the streptavidin pulldown fraction from TurboID‐UFL1‐expressing cells in the presence of biotin, whereas no enrichment was detected in TurboID‐empty vector‐expressing or biotin‐free control cells (Figure 2E). These results provide independent evidence that UFL1 and ENO1 reside within close spatial proximity in CRC cells. Domain‐mapping analysis revealed that UFL1 associated with ENO1 through its N‐terminal region (amino acids 1–247 and 247–530) (Figure 2F,G). Immunofluorescence staining further demonstrated the cytoplasmic colocalization of these two proteins in CRC cells (Figure 2H).

**FIGURE 2:**
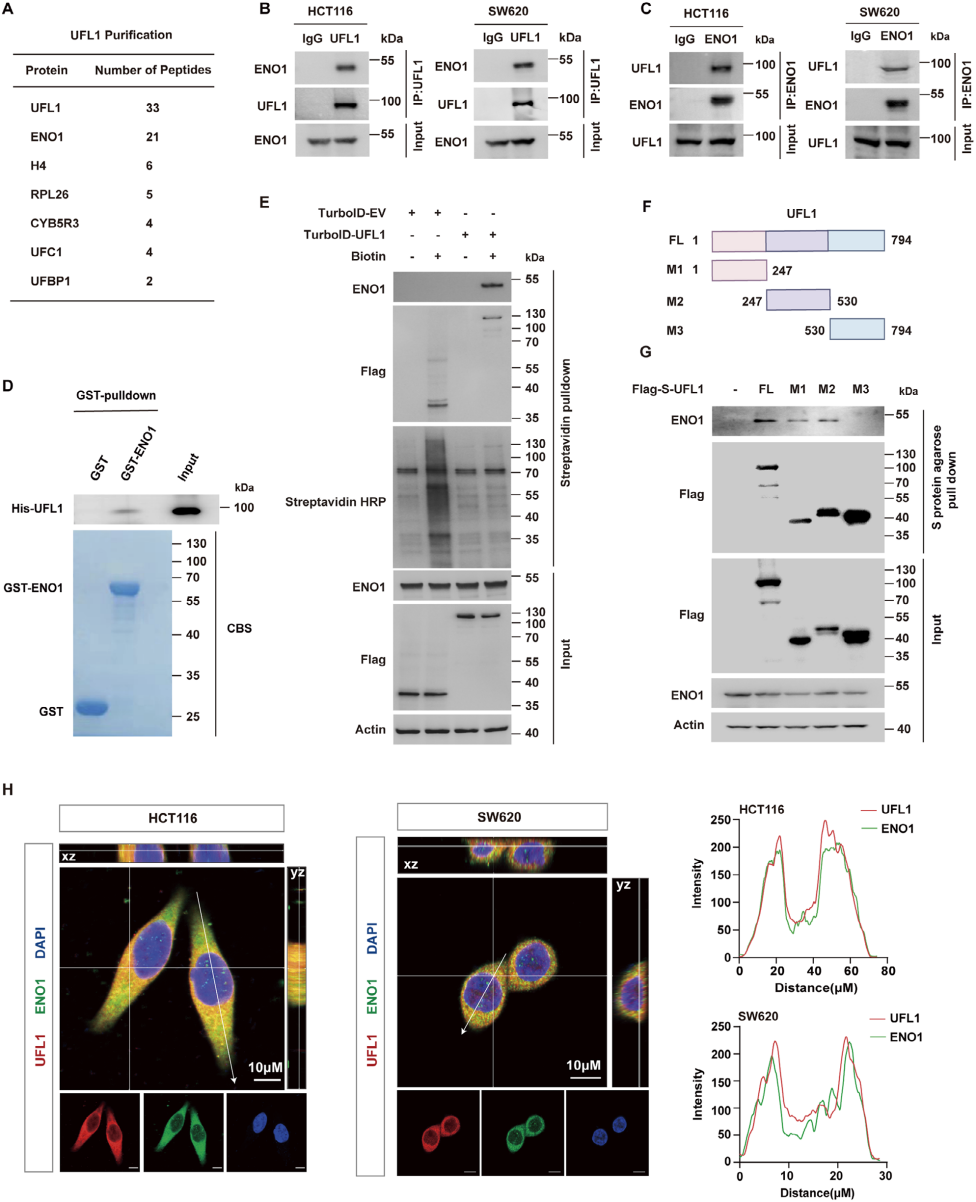
UFL1 directly interacts with ENO1. (A) UFL1‐associated proteins identified by affinity purification followed by mass spectrometry in HCT116 cells stably expressing Flag‐UFL1. (B, C) Co‐immunoprecipitation assays in CRC cells using control IgG, anti‐UFL1 (B) or anti‐ENO1 (C) antibodies, followed by immunoblotting with the indicated antibodies. (D) In vitro binding assay using purified recombinant GST, GST‐ENO1, and His‐UFL1 proteins. Protein interaction was detected by immunoblotting. Coomassie blue staining (CBS) was used as loading control. (E) HCT116 cells expressing TurboID‐EV or TurboID‐UFL1 were incubated with biotin (50 µM, 30 min) to induce proximity‐dependent biotinylation. Streptavidin pulldown followed by immunoblotting was performed to detect biotinylated proteins and candidate proximal interactors. ENO1 was specifically enriched in the streptavidin pulldown fraction under TurboID–UFL1 expression in the presence of biotin. Input lysates confirmed comparable ENO1 expression and similar levels of TurboID constructs (Flag). Global protein biotinylation was detected by streptavidin‐HRP. Actin was used as a loading control for input samples. (F) Schematic diagram of UFL1 constructs, including full‐length (FL), M1 (1‐247 aa), M2 (247‐530 aa) and M2 (530‐794 aa) truncations. (G) Cells expressing pIRES‐UFL1 (containing Flag and S tag) or its truncation mutants were subjected to pull‐down using S‐protein agarose. (H) Co‐localization of endogenous ENO1 (green) and UFL1 (red) was detected by immunofluorescence staining in CRC cells. Nuclei were counterstained with DAPI (blue). Scale bar, 10 µm. Colocalization analysis was performed on orthogonal views of confocal microscopy images and raw microscopy images are available in Data S2. Raw blots are available in Data S3.

### 2.3 UFL1 Suppresses CRC Cell Proliferation by Inhibiting ENO1 Enzymatic Activity

Previous studies have shown that UFL1‐mediated UFMylation can regulate substrate stability [14, 19]. We therefore examined whether UFL1 regulates ENO1 expression. UFL1 depletion did not alter ENO1 protein abundance in CRC cells (Figure 3A and Figure S2A) but markedly increased its enzymatic activity (Figure 3B). Because ENO1 functions as an active dimer [20], we next assessed ENO1 dimerization and found that UFL1 depletion enhanced ENO1 dimer formation (Figure 3C,D and Figure S2B). These results suggest that UFL1 inhibits ENO1 enzymatic activity by limiting the formation of its active dimer.

**FIGURE 3:**
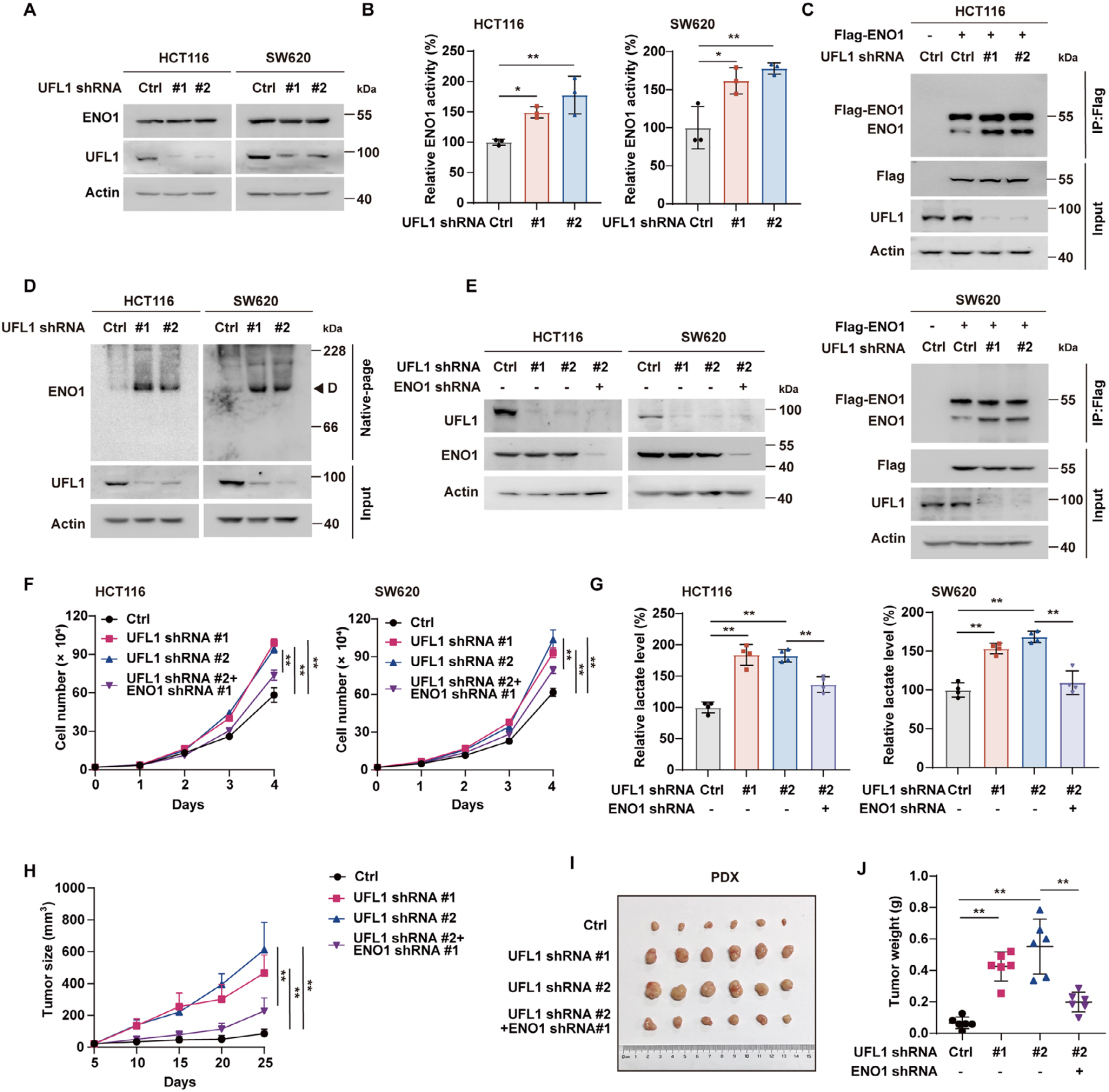
UFL1 suppresses CRC cell proliferation by inhibiting ENO1 enzymatic activity. (A) HCT116 and SW620 cells stably expressing control (Ctrl) or UFL1 shRNAs were generated, and cell lysates were immunoblotted with the indicated antibodies. (B) ENO1 activity assay in HCT116 and SW620 cells stably expressing control (Ctrl), or UFL1 shRNAs (n = 3). (C) HCT116 and SW620 cells stably expressing control (Ctrl) or UFL1 shRNAs were stably transfected with Flag‐tagged ENO1. ENO1 dimerization was assessed by pull‐down with anti‐Flag affinity gel followed by immunoblotting. (D) HCT116 and SW620 cells stably expressing control (Ctrl) or UFL1 shRNAs were generated, and endogenous ENO1 dimers was detected by native‐PAGE under nonreducing conditions (D, dimeric ENO1). (E) Western blotting analysis confirming UFL1 or ENO1 knockdown in HCT116 and SW620 cells. (F, G) Cell proliferation (F) (n = 3) and lactate production(G) (n = 4) assays in HCT116 and SW620 cells stably expressing control (Ctrl), UFL1 or ENO1 shRNAs. (H‐J) Representative tumor growth curve from CRC patient‐derived xenografts (PDXs) in control and experimental groups(H). Analysis of tumor image (I) and tumor weight (J) (n = 6). Error bars represent the SD. Statistical significance were determined using one‐way ANOVA followed by Tukey's multiple comparisons test (B, F, G, H, J); ^**^ *p* < 0.01, ^*^ *p* < 0.05. Raw blots are available in Data S3.

Consistent with its reported tumor‐promoting role [21], ENO1 was expressed at relatively higher levels in CRC cells than in normal colon epithelial cells NCM460 (Figure S2C). Moreover, ENO1 depletion markedly suppressed CRC cell proliferation and lactate production (Figure S2D–F). Given that ENO1 also functions as a cell surface plasminogen receptor and a nuclear transcriptional regulator, we next examined whether UFL1 influences these non‐glycolytic functions. UFL1 depletion did not affect integrin αvβ3 expression or *c‐Myc* mRNA levels in CRC cells, two established downstream readouts of the non‐glycolytic functions of ENO1 (Figure S2G, H). These results suggest that UFL1 may primarily modulate the metabolic function of ENO1 rather than its non‐glycolytic activities in CRC cells. Importantly, the increased proliferation and lactate production induced by UFL1 depletion were largely reversed by concomitant ENO1 knockdown (Figure 3E–G). Similar effects were observed in both cell‐derived xenograft (CDX) and patient‐derived xenograft (PDX) models (Figure 3H–J and Figure S2I–L). Together, these results demonstrate that UFL1 suppresses CRC progression by inhibiting ENO1 dimerization and enzymatic activity.

### 2.4 UFL1 Catalyzes UFMylation of ENO1 at Lys285 and Lys420

To determine whether UFL1 regulates ENO1 through UFMylation, we co‐expressed ENO1 with the core UFMylation machinery components (UBA5, UFC1, UFL1, UFBP1 and UFM1ΔC2). A UFMylated ENO1 species exhibiting the characteristic 18 kD mobility shift was detected in both in vivo and in vitro assays (Figure 4A,B). Endogenous ENO1 UFMylation was confirmed in CRC cells and PDX tumor samples and was markedly diminished upon UFL1 depletion (Figure 4C and Figure S3A).

**FIGURE 4:**
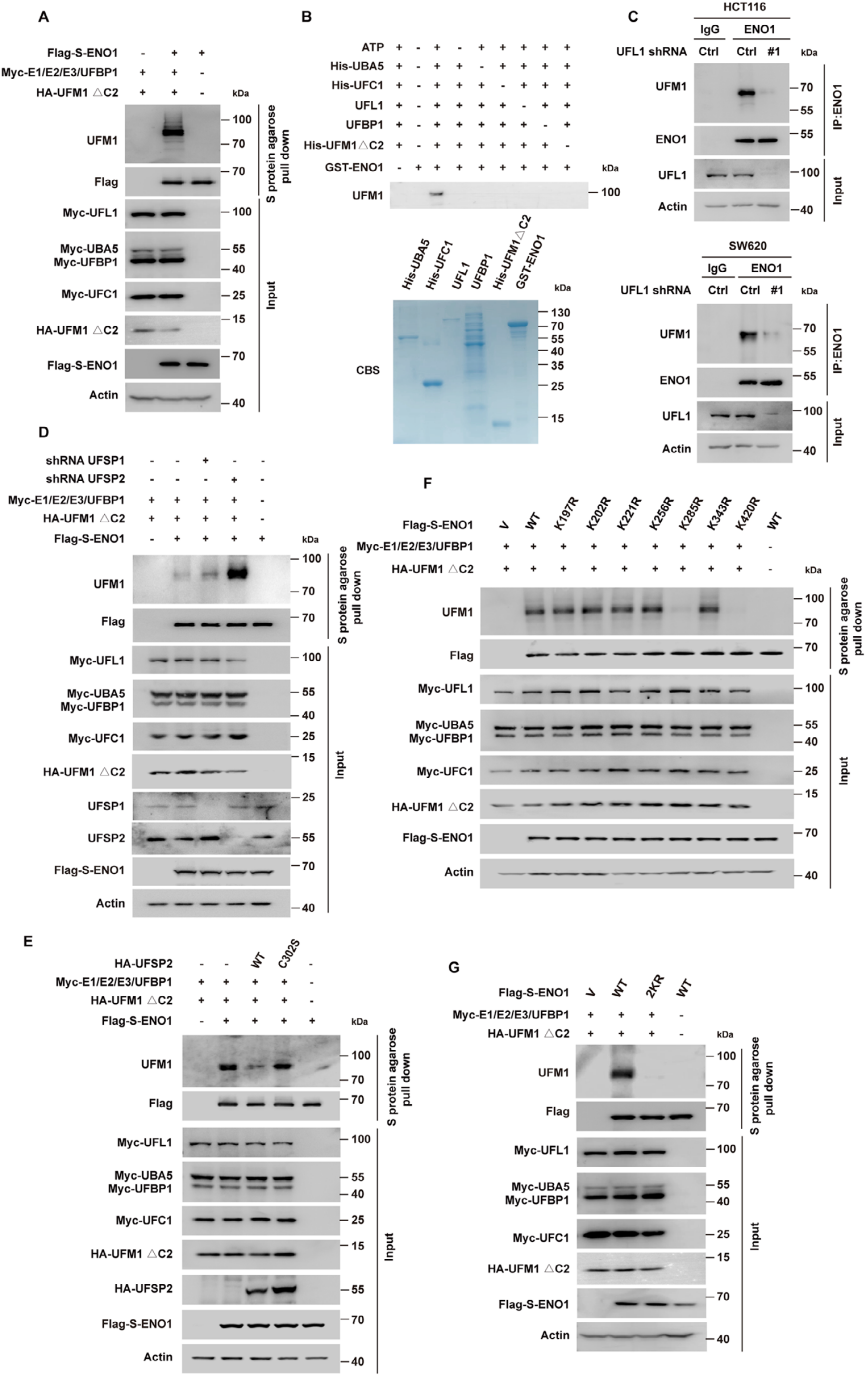
UFL1 catalyzes UFMylation of ENO1 at Lys285 and Lys420. (A, B) In vivo (A) and in vitro (B) UFMylation assays demonstrating the UFMylation of ENO1. (C) Endogenous UFMylation of ENO1 was detected by immunoprecipitation using anti‐ENO1 antibody, followed by immunoblotting with anti‐UFM1 antibody in HCT116 and SW620 cells stably expressing control (Ctrl) or UFL1 shRNA. (D) In vivo UFMylation assays showing that ENO1 UFMylation is regulated by UFSP2 in HEK293T cells with knockdown of UFSP1 or UFSP2. (E) In vivo UFMylation assays showing that ENO1 UFMylation is regulated by UFSP2 in HEK293T cells co‐expressing HA‐vector, HA‐UFSP2 or HA‐UFSP2 C302S. (F) Mutational analysis of seven lysine residues in full‐length Flag‐S‐ENO1 to identify potential UFMylation sites. HEK293T cells were transfected with vector control (V), wildtype FlagSENO1 (WT), or the indicated lysine mutants, together with the UFMylation machinery components, and subjected to an in vivo UFMylation assay. (G) In vivo UFMylation assay comparing vector control (V), Flag‐S‐ENO1 wild‐type (WT), and the K285/K420R (2KR) mutant in HEK293T cells co‐expressing the indicated UFMylation‐machinery components. Raw blots are available in Data S3.

UFSP1 and UFSP2 together maintain the dynamic reversibility of protein UFMylation in cells [10]. Among them, UFSP2 is more abundantly expressed in human tissues and serves as the predominant active de‐UFMylase [22]. Consistent with this, UFSP2 knockdown markedly increased ENO1 UFMylation, whereas depletion of UFSP1 exerted only a modest effect (Figure 4D). Moreover, ectopic expression of wild‐type UFSP2 (WT), but not the catalytically inactive C302S mutant, significantly reduced ENO1 UFMylation (Figure 4E). These results identify UFSP2 as the principal de‐UFMylase responsible for removing UFM1 from ENO1 in CRC cells.

Further mapping analysis revealed that UFMylation occurred within the full‐length and C‐terminal region of ENO1 (amino acids 140–434) (Figure S3B, C). To identify UFMylation sites, we generated a series of lysine (K) to arginine (R) mutants within this region. Single mutations at K285R or K420R partially reduced UFMylation (Figure 4F), whereas the double K285/420R mutant (2KR) almost completely abolished UFMylation (Figure 4G). These results identify K285 and K420 as the principal UFMylation sites on ENO1.

### 2.5 UFL1‐Mediated UFMylation Inhibits ENO1 Activity and CRC Progression

We next examined how UFMylation at K285 and K420 affects ENO1 function. A co‐immunoprecipitation assay using HA‐ and Flag‐tagged ENO1 showed that the 2KR mutant displayed a stronger self‐association than wild‐type ENO1 (Figure 5A). Native PAGE performed under non‐reducing conditions further confirmed increased formation of dimeric ENO1 in the 2KR mutant (Figure 5B). To assess the functional consequence of this modification, ENO1‐deficient CRC cells were reconstituted with ENO1 WT or 2KR mutant. Compared to WT ENO1, expression of the 2KR mutant significantly increased ENO1 enzymatic activity, glycolytic flux, cell proliferation, and lactate production (Figure 5C–G and Figure S4A, B). Consistent with these findings, tumors reconstituted with the ENO1 2KR mutant exhibited accelerated growth in both CDX and PDX models (Figure 5H–K and Figure S4C–F). Collectively, these results demonstrate that UFMylation of ENO1 at K285 and K420 inhibits its enzymatic activity by preventing ENO1 dimerization, thereby suppressing CRC progression.

**FIGURE 5:**
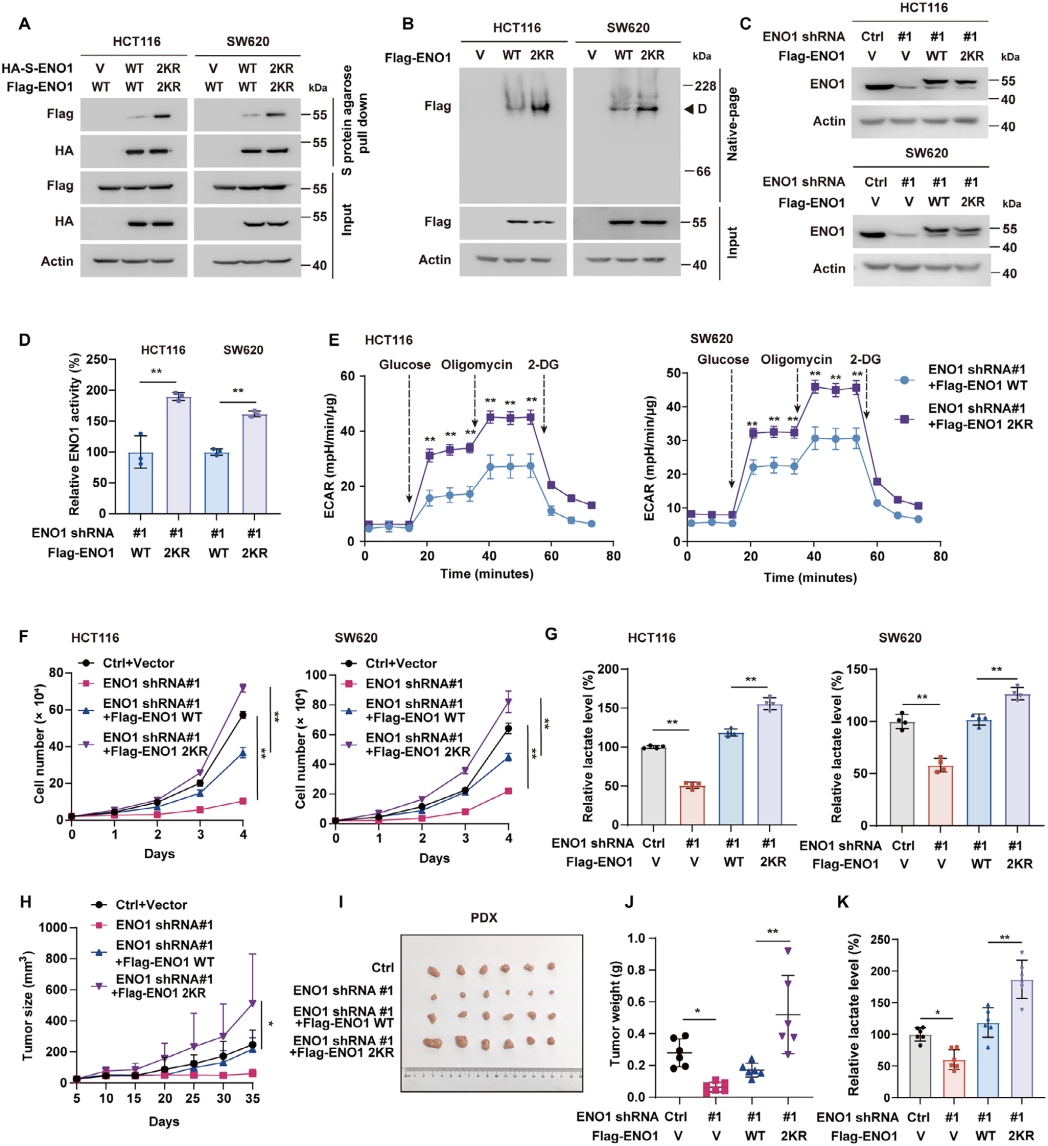
UFL1‐mediated UFMylation inhibits ENO1 activity and CRC progression. (A) HCT116 and SW620 cells stably expressing HA‐S and Flag‐ tagged wild‐type ENO1 or K285/K420R (2KR) mutants was subjected to pull‐down using S‐protein agarose followed by immunoblotting. V denotes vector control (emptyvector transfection). (B) HCT116 and SW620 cells stably expressing vector control(V), Flag‐ENO1(WT) or K285/K420R (2KR) mutants. Dimeric Flag‐ENO1 was detected by native‐PAGE under nonreducing conditions (D, dimeric Flag‐ENO1). (C) Western blotting analysis confirming endogenous ENO1‐depleted HCT116 and SW620 cells reconstituted with the indicated plasmids. V denotes vector control. (D‐G) ENO1 activity(D) (n = 3), Seahorse assay(E) (n = 4), Cell proliferation (F) (n = 3) and Lactate production (G) (n = 4) assays in endogenous ENO1‐depleted HCT116 and SW620 cells reconstituted with the indicated plasmids. V denotes vector control. (H‐J) Representative tumor growth curve from CRC patient‐derived xenografts (PDXs) in control and experimental groups(H). Analysis of tumor image (I) and tumor weight (J) (n = 6). (K) Tumors from PDX models were homogenized. Lactate levels were measured (n = 6). Error bars represent the SD. Statistical significance was determined using an unpaired Student's t‐test (D, E) or one‐way ANOVA followed by Tukey's multiple comparisons test (F, G, H, J, K); ^**^ *p* < 0.01, ^*^ *p* < 0.05. Raw blots are available in Data S3.

### 2.6 Torezolid Enhances the UFL1‐ENO1 Interaction and Potentiates the Antitumor Efficacy of 5‐FU

Given that ENO1 UFMylation restrains CRC progression, we hypothesized that pharmacological enhancement of the UFL1‐ENO1 interaction could provide therapeutic benefit. To test this hypothesis, we established a chemiluminescence‐based protein‐protein interaction assay using HCT116 cells expressing Flag‐UFL1 (amino acids 1–530) and screened an FDA‐approved drug library (Figure 6A). Several compounds increased the UFL1‐ENO1 interaction by more than 3.5‐fold, among which torezolid exhibited the strongest effect (Figure 6B). This interaction‐enhancing activity was validated in vitro using purified GST‐ENO1 and His‐UFL1 proteins and was further confirmed in CRC cells (Figure 6C,D). In addition, torezolid treatment increased ENO1 UFMylation, disrupted ENO1 dimerization, and reduced its enzymatic activity (Figure 6E–H). Consistent with these findings, torezolid markedly suppressed CRC cell proliferation and decreased lactate production (Figure 6I,J).

**FIGURE 6:**
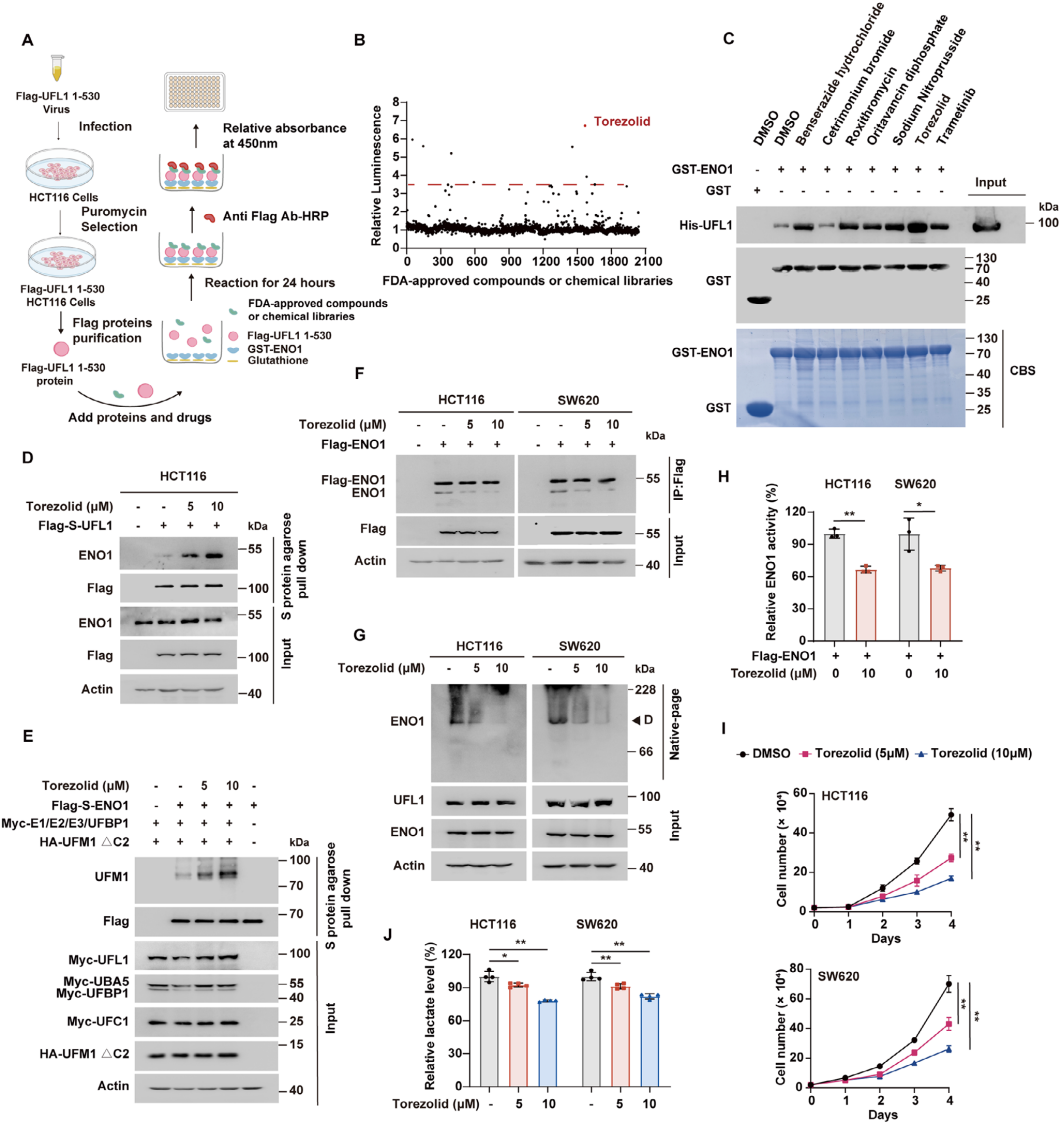
Torezolid enhances the UFL1‐ENO1 interaction and suppresses CRC cell growth. (A) Schematic diagram of the experimental procedure for the chemiluminescence‐based protein‐protein interaction assay. (B) Chemiluminescence assay was performed to screen compounds enhancing the UFL1‐ENO1 interaction. (C) In vitro binding assay using purified recombinant GST, GST‐ENO1, and His‐UFL1 proteins. DMSO or compounds (10 µM) were incubated with proteins for 24 h. The UFL1‐ENO1 interaction was detected by immunoblotting. Coomassie blue staining (CBS) was used as loading control. (D) HCT116 were transfected with empty vector or Flag‐UFL1 and treated with DMSO or torezolid for 24 h, and subjected to pull‐down using S‐protein agarose. (E) In vivo UFMylation assays showing that torezolid enhanced the UFMylation of ENO1. (F) HCT116 and SW620 cells stably expressing vector or Flag‐ENO1 were treated with DMSO or torezolid for 24 h. ENO1 dimerization was assessed by pull‐down with anti‐Flag affinity gel followed by immunoblotting. (G) HCT116 and SW620 cells were treated with DMSO or torezolid for 24 h. Endogenous ENO1 dimers was detected by native‐PAGE under nonreducing conditions (D, dimeric ENO1). (H‐J) ENO1 activity (H) (n = 3), Cell proliferation (I) (n = 3), and lactate production (J) (n = 4) assays in HCT116 and SW620 cells were treated with DMSO or torezolid. Error bars represent the SD. Statistical significance was determined using an unpaired Student's t‐test (H) or one‐way ANOVA followed by Tukey's multiple comparisons test (I, J); ^**^ *p* < 0.01, ^*^ *p* < 0.05. Raw blots are available in Data S3.

To evaluate the mechanistic specificity of torezolid, we performed loss‐of‐function studies to determine whether its anti‐tumor effects depend on the ENO1‐UFL1 axis. Notably, torezolid largely lost its ability to suppress cell proliferation and lactate production in CRC cells depleted of either UFL1 or ENO1 (Figure S5A–D), indicating that its anti‐glycolytic and anti‐proliferative effects require an intact UFL1‐ENO1 axis. Furthermore, rescue experiments using the UFMylation‐deficient ENO1 2KR mutant revealed that the inhibitory effects of torezolid on cell proliferation and lactate production were markedly attenuated in cells expressing the ENO1 2KR mutant compared with those expressing ENO1 WT (Figure S5E, F). Collectively, these results demonstrate that the anti‐tumor activity of torezolid in CRC is largely mediated through the UFL1‐ENO1 axis.

To further assess the therapeutic potential of torezolid, we examined its efficacy in combination with 5‐fluorouracil (5‐FU). Co‐treatment with torezolid and 5‐FU produced enhanced inhibition of tumor growth in CRC cell lines and PDOs (Figure 7A–D). In a PDX model, each agent alone produced moderate tumor suppression, whereas the combination treatment elicited the most pronounced antitumor effect without affecting body weight (Figure 7E–H and Figure S6). Consistent with these findings, the combination treatment further reduced Ki67 staining and increased cleaved caspase‐3 levels in tumor tissues, indicating decreased proliferation and enhanced apoptosis (Figure 7I,J). Collectively, these results demonstrate that torezolid potentiates the antitumor efficacy of 5‐FU by promoting UFL1‐mediated UFMylation and functional inactivation of ENO1.

**FIGURE 7:**
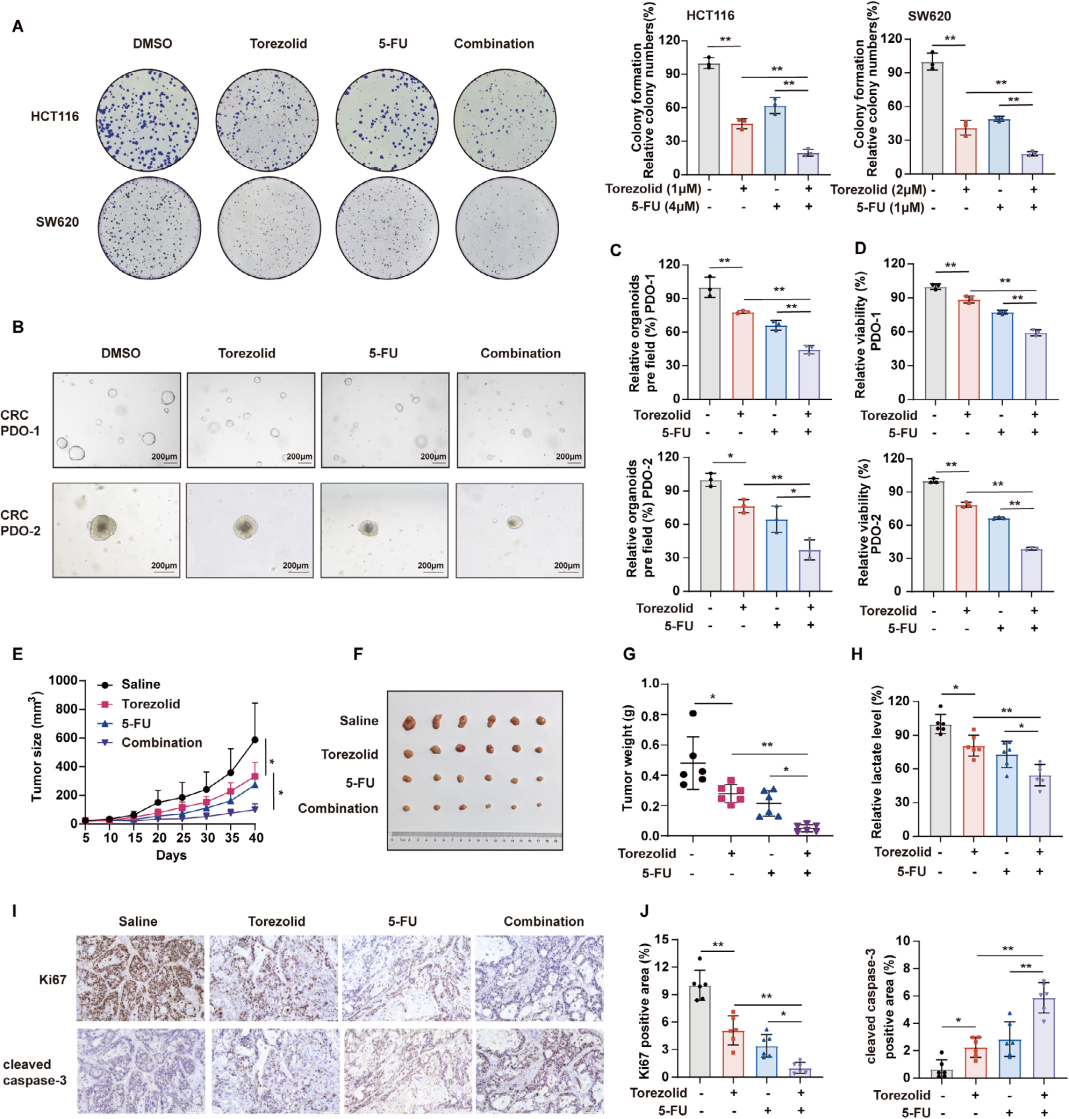
Torezolid potentiates the antitumor efficacy of 5‐FU. (A) Colony formation was determined in HCT116 and SW620 cells treated with torezolid and 5‐FU, alone or in combination (n = 3). (B) Representative images of CRC patient‐derived organoids treated with torezolid and 5‐FU, alone or in combination. Scale bar, 200 µm. (C) Quantification of organoids ≥100 µm in diameter per field (n = 3). (D) Cell viability assays were performed to assess the effect of drug treatment on CRC organoid viability (n = 3). (E‐G) Representative tumor growth curve from CRC patient‐derived xenografts (PDXs) in control and experimental groups (E). Analysis of tumor image (F) and tumor weight (G) (n = 6). (H) Tumors from PDX models were homogenized and lactate levels were measured (n = 6). (I) Representative images of Ki67 and cleaved caspase‐3 staining in xenograft models. Scale bar, 100 µm. (J) Quantification of the Ki67 and cleaved caspase‐3 in tumors (n = 6). Error bars represent the SD. Statistical significance was determined using one‐way ANOVA followed by Tukey's multiple comparisons test (A,C,D,E,G,H,J); ^**^ *p* < 0.01, ^*^ *p* < 0.05. Raw microscopy images are available in Data S2. Raw blots are available in Data S3.

## 3 Discussion and Conclusion

In this study, we identify a previously unrecognized role of the UFM1 E3 ligase UFL1 in suppressing aerobic glycolysis via UFMylation of the glycolytic enzyme ENO1. We show that UFL1 catalyzes UFMylation of ENO1 at lysine residues K285 and K420, thereby reducing its enzymatic activity by inhibiting active dimer formation and ultimately restraining CRC progression. Notably, screening of FDA‐approved compounds revealed that the antibiotic torezolid markedly enhances the UFL1‐ENO1 interaction and potentiates the antitumor efficacy of 5‐FU in both PDOs and PDX models. These findings define the UFL1‐ENO1 axis as a post‐translational regulatory mechanism that restricts glucose metabolism and highlight its potential as a therapeutic target in CRC.

UFMylation has emerged as a multifunctional post‐translational modification involved in a wide range of physiological and pathological processes, including embryonic development [23], endoplasmic reticulum homeostasis [24], immune regulation [16, 25], and DNA damage response [15, 26]. Despite these advances, the biological roles of UFMylation in cancer metabolism have remained poorly understood. A recent study demonstrated that UFL1‐catalyzed UFMylation antagonizes PD‐1 ubiquitination and degradation in T cells, thereby suppressing anti‐tumor immunity, which is disrupted by the metabolic sensor AMPK [16]. Another study reported that UFL1‐mediated UFMylation of p53 antagonizes MDM2‐dependent ubiquitination, thereby stabilizing p53 and enhancing its tumor‐suppressive function in colorectal cancer [14]. Given that p53 represses the Warburg effect by downregulating glycolytic genes such as GLUT1, GLUT4, and HK2 [27]. These findings suggest that UFMylation may influence cellular metabolism. However, whether UFMylation directly regulates core metabolic pathways has remained unclear.

Our results establish a mechanistic link between UFMylation and metabolic control by demonstrating that UFL1 suppresses aerobic glycolysis through direct UFMylation of ENO1. UFL1 depletion increases lactate accumulation even in p53‐/‐ HCT116 cells (Figure S1D). Importantly, neither knockdown of UFL1 nor the disruption of ENO1 UFMylation altered ENO1 protein abundance but instead modulated ENO1 dimerization and enzymatic activity (Figures 3, 5 and Figure S2A, B). These results indicate that UFL1 negatively regulates glycolysis through a post‐translational modification‐dependent mechanism rather than via p53‐dependent transcriptional regulation. Importantly, the incomplete rescue of UFL1‐loss phenotypes by ENO1 depletion suggests that ENO1 may not be the sole downstream effector of UFL1 in CRC. Indeed, our IP‐MS analysis identified several additional UFL1‐interacting proteins, including histone H4, CYB5R3, and RPL26, which have been implicated in metabolic regulation (Figure 2A and Data S1). For instance, Histone H4 has been linked to chromatin remodeling and transcriptional regulation of metabolic genes [28]. CYB5R3 plays an important role in cellular redox homeostasis and mitochondrial metabolism [29]. RPL26 has been reported to participate in the translational control of metabolism‐related proteins [30]. It will therefore be important to investigate whether these additional UFL1 substrates contribute to tumor metabolism and progression in the future.

ENO1 is a multifunctional protein frequently overexpressed and hyperactivated in human cancers, where it promotes tumor proliferation, metastasis, and therapeutic resistance [31]. In addition to its canonical metabolic role, ENO1 also participates in non‐metabolic processes, including plasminogen binding at the cell surface and the regulation of gene transcription and protein translation through interactions with DNA and mRNA in the nucleus [32, 33, 34, 35]. In this study, UFL1 depletion did not significantly alter αvβ3 integrin expression or *c‐Myc* transcription in CRC cells (Figure S2G, H). Consistent with this observation, immunofluorescence analyses revealed that UFL1 predominantly co‐localizes with ENO1 in the cytoplasm (Figure 2H). These findings suggest that UFL1‐mediated UFMylation primarily regulates the cytoplasmic metabolic function of ENO1 rather than its non‐metabolic activities in CRC cells.

Emerging evidence indicates that ENO1 function is tightly regulated by a complex network of post‐translational modifications, including phosphorylation, methylation, acetylation, ubiquitylation, 2‐hydroxyisobutyrylation and O‐GlcNAcylation [20, 36, 37, 38, 39, 40, 41]. For instance, PRMT5 and PRMT6 mediate symmetric and asymmetric dimethylation of ENO1 at R9 and R372 in ovarian and lung cancers, respectively, thereby enhancing its enzymatic activity [20, 37]. Conversely, ULK1/2‐mediated phosphorylation of ENO1 at S115 and S282 suppresses ENO1 activity [36], whereas O‐GlcNAcylation at T19 enhances its enzymatic function [40]. Our study adds UFMylation to this expanding repertoire of ENO1 post‐translational modifications. We demonstrate that UFMylation at K285 and K420 inhibits ENO1 dimerization and enzymatic activity, thereby limiting glycolytic flux and CRC growth (Figure 5). Mechanistically, the conjugation of the bulky UFM1 moiety to ENO1 may sterically hinder dimer assembly. Given that dimerization is indispensable for ENO1 catalytic activity, disruption of dimer assembly likely represents a key mechanism by which UFMylation suppresses ENO1 metabolic function. However, the precise structural basis of UFMylation‐dependent regulation of ENO1 remains to be established and warrants further investigation using biochemical and high‐resolution structural analyses. In addition, whether UFMylation crosstalks with other post‐translational modifications to indirectly regulate ENO1 dimerization and activity warrants further investigation. Notably, K420 has previously been identified as a critical ubiquitination site in TP53‐mutant colorectal cancer [42], suggesting potential crosstalk between UFMylation and ubiquitination in regulating ENO1. Understanding this interplay will be crucial for deciphering the multilayered metabolic control in cancer.

Given the critical role of ENO1 in aerobic glycolysis and tumor progression, it has been proposed as a therapeutic target in cancer. ENOblock, a non‐substrate analogue enolase inhibitor, suppresses ENO1 activity and tumor progression in several cancer types [43, 44], while the ENO1 monoclonal antibody HuL227 exerts antitumor effects by inhibiting its non‐metabolic function [45]. However, direct pharmacological inhibition of ENO1 remains challenging due to limited selectivity, systemic toxicity, and tumor heterogeneity [6, 21], highlighting the need for alternative therapeutic strategies. Our study proposes an alternative strategy for modulating ENO1 activity indirectly through post‐translational modification. We identify torezolid as a small‐molecule enhancer of the UFL1‐ENO1 interaction that increases ENO1 UFMylation, reduces its enzymatic activity, and suppresses CRC growth. Notably, combination therapy with torezolid and 5‐FU achieved superior antitumor efficacy in both PDOs and PDX models without detectable toxicity (Figure 7 and Figure S6). Although torezolid has demonstrated a favorable safety profile in previous studies [46], comprehensive toxicity assessments were beyond the scope of this work and warrant further investigation. In addition, future studies using orthotopic and metastatic CRC models will be important to further define the role of the UFL1‐ENO1 axis in tumor progression and to facilitate clinical translation. Despite these limitations, our findings highlight the translational potential of pharmacologically enhancing UFL1‐mediated UFMylation to suppress glycolytic metabolism and improve chemotherapy response. However, as an FDA‐approved antibiotic, torezolid likely has additional cellular targets beyond the UFL1‐ENO1 axis. Therefore, developing more selective compounds that specifically enhance the UFL1‐ENO1 interaction represents an important future direction. In this regard, high‐resolution structural characterization of the UFL1‐ENO1 complex using cryo‐EM, X‐ray crystallography, or advanced computational modeling may facilitate structure‐guided optimization of torezolid and rational design of next‐generation UFMylation modulators with improved selectivity and reduced off‐target activities. More broadly, given the central role of metabolic reprogramming in cancer, it will be important to determine whether the UFL1‐dependent regulation of ENO1 represents a conserved metabolic vulnerability across tumor types. Future studies exploring the therapeutic efficacy of torezolid or related compounds in additional malignancies may help establish the broader translational relevance of targeting the UFL1‐ENO1‐glycolysis axis.

In conclusion, our study identifies UFL1 as a metabolic regulator that suppresses aerobic glycolysis through UFMylation of ENO1, thereby inhibiting CRC progression. Our findings establish a mechanistic link between UFMylation and metabolic reprogramming and suggest that pharmacological enhancement of the UFL1‐ENO1 axis, for example through torezolid or related compounds, represents a promising therapeutic strategy for colorectal cancer.

## 4 Experimental Section

### 4.1 Cell Culture, Plasmids, and Antibodies

HCT116, SW620, SW480, LoVo, HT‐29, and HEK293T cell lines were purchased from ATCC (American Type Culture Collection, Maryland, USA). Cell lines were mycoplasma‐free and authenticated by short tandem repeat DNA profiling analysis. HCT116 p53 WT and p53−/− cells were kindly provided by Dr. Jihong Zhang from Kunming University of Science and Technology. HCT116 and HT‐29 cells were cultured in McCoy's 5A medium (Thermo Fisher Scientific, MA, USA) with 10% fetal bovine serum (FBS, Bidepharm, Shanghai, China). LoVo cells were cultured in F‐12K medium (Thermo Fisher Scientific, MA, USA) with 10% FBS. HEK293T cells were maintained in Dulbecco's Modified Eagle's medium (DMEM, Thermo Fisher Scientific, MA, USA) with 10% FBS. Cells were maintained at 37°C in a humidified incubator containing 5% CO_2_. SW480 and SW620 cells were cultured in Leibovitz's L‐15 medium (Thermo Fisher Scientific, MA, USA) with 10% FBS at 37°C without CO_2_.

Plasmids UFL1 and ENO1 were cloned into pIRES‐Flag‐S, pLV.3‐Flag, pLV.5‐HA‐S, pLV.6‐GFP, pET28a and pGEX4T‐1 vectors. Plasmids UBA5, UFC1, UFL1, UFBP1, and UFM1ΔC2 were cloned into pRSF, pET28a, pCMV‐HA and pcDNA3.1‐Myc. The UFSP2 plasmid was cloned into pCMV‐HA. TurboID‐Empty vector (EV) and TurboID‐UFL1 plasmids were kindly provided by Dr. Haoxing Zhang and Jiaxin Tang (Shenzhen University). All site mutants were generated by site‐directed mutagenesis (TOYOBO, Osaka, Japan) and identified by sequencing.

Anti‐UFL1 (A303‐456A, dilution: 1:1000) was purchased from Bethyl. Anti‐UFM1 (ab109305, dilution: 1:1000) and anti‐His (ab9108, dilution: 1:1000) were purchased from Abcam. Anti‐ENO1 (11204‐1‐AP, dilution: 1:2000), anti‐UFSP2 (16999‐1‐AP, dilution: 1:1000), anti‐UFSP1 (14655‐1‐AP, dilution: 1:1000) and anti‐p53 (60283‐2‐Ig, dilution: 1:1000) were purchased from Proteintech Group. Anti‐Myc (SC‐40, dilution: 1:1000) was purchased from Santa Cruz Biotechnology. Anti‐Flag (F1804, dilution: 1:1000), anti‐HA (H3663, dilution: 1:1000) and anti‐β‐Actin (A1978, dilution: 1:5000) antibodies were purchased from Sigma‐Aldrich. Anti‐Integrin αV (SC56‐07, dilution: 1:1000) and anti‐Integrin β3 (SJ19‐09, dilution: 1:500) were purchased from HUABIO. All primary antibodies listed above were used in TBST buffer with 5% non‐fat milk for Western blotting. The following light‐chain‐specific secondary antibodies were used where appropriate to avoid detection of IgG heavy chains: peroxidase‐AffiniPure goat anti‐mouse IgG light‐chain‐specific (Jackson ImmunoResearch, 115‐035‐174, dilution: 1:2000), peroxidase IgG fraction monoclonal mouse anti‐Rabbit IgG, light‐chain‐specific (Jackson ImmunoResearch, 211‐032‐171, dilution: 1:2000). Anti‐Flag affinity gel (A2220) and S‐protein agarose (69704‐4) were purchased from Sigma‐Aldrich. Pierce Glutathione agarose (16100) and Pierce Protein G agarose (20399) were purchased from Thermo Scientific. Streptavidin magnetic beads (SA021005) were purchased from Smart‐lifesciences.

### 4.2 Immunoblot and Coimmunoprecipitation Assay

Cells were lysed in NETN buffer (20 mM Tris‐HCl, pH 8.0, 300 mM NaCl, 1 mM ethylenediaminetetraacetic acid (EDTA) and 0.5% NP‐40) supplemented with protease inhibitor cocktail (Roche), 10 mM β‐glycerophosphate, 1 mM sodium orthovanadate, 10 mM sodium fluoride, and 1 mM phenylmethylsulfonyl fluoride (PMSF). The lysates were incubated on ice for 30 min. Whole cell lysates were resolved by SDS‐polyacrylamide gel electrophoresis (SDS‐PAGE) and immunoblotted with the indicated antibodies. For immunoprecipitation, lysates were incubated with S‐protein agarose for 4 h or anti‐Flag affinity gel for 2 h at 4 °C. The immunoprecipitates were washed four times with lysis buffer and then analyzed by immunoblotting.

### 4.3 Single‐Cell RNA‐Seq Data Processing

Single‐cell RNA‐seq datasets with available processed count matrices or Cell Ranger‐generated output files were obtained from public repositories (Table S2). In total, 102 samples from 7 independent scRNA‐seq datasets were analyzed. All datasets were aligned to the human reference genome GRCh38. Data preprocessing and downstream analysis were performed using the Seurat R package [47]. Quality control was performed to remove low‐quality cells with fewer than 500 or more than 8,000 detected genes, and those with> 20% mitochondrial transcript content. Harmony algorithm was applied to minimize technical and study‐specific batch effects [48]. Filtered matrices were then normalized using the Seurat NormalizeData function (default parameters), and the top 2,000 highly variable genes (HVGs) were identified for subsequent analyses. Principal component analysis (PCA) was performed on HVGs, and the number of principal components (PCs) retained for downstream analysis was determined based on elbow plots and Jackstraw statistics. Clustering was performed using the Seurat FindClusters function over a range of resolution parameters (0.5‐2.0), and the resulting embeddings were visualized using Uniform Manifold Approximation and Projection (UMAP). Cluster‐specific marker genes were identified using the Wilcoxon rank‐sum test implemented in the FindMarkers function. Major cell lineages were annotated based on canonical markers, including epithelial cells (EPCAM, KRT18, KRT19), T/NK cells (CD3D, CD3E, CD4, CD8A, CD96, KLRF1, NKG7), B cells (CD79A, CD79B, MS4A1), plasma cells (IGHG1, MZB1, IGKC), mast cells (TPSAB1, TPSB2, CPA3), myeloid cells (CD14, FCGR3A, CD68, CD83, CSF1R), fibroblasts (COL1A1, COL1A2, DCN), pericytes (RGS5, PDGFRB), and endothelial cells (PECAM1, CLDN5, VWF). Malignant epithelial cells were inferred using inferCNV (https://github.com/broadinstitute/inferCNV), with genomically stable immune cells designated as the reference population and epithelial cells as the observation group, enabling evaluation of large‐scale copy‐number alterations and tumorigenic genomic instability. Single‐cell glycolysis pathway activity was quantified using AUCell [49], with the gene set derived from the HALLMARK collection.

### 4.4 RNA Interference

*UFL1, UFSP1* and *UFSP2* shRNAs were cloned into the pLKO.1‐puro vector (Addgene #8453). For double knockdown experiments, *ENO1* shRNAs were cloned into both the pLKO.1‐puro and pLKO.1‐hygro (Addgene #24150) vectors. The shRNA targeting sequences for UFL1 #1 and #2 shRNAs are 5′‐CCAGTAAGCATAAGTCATATT‐3′ and 5′‐CCACCTCATACACACACAATT‐3′, respectively. The sequences for ENO1 #1 and #2 shRNAs are 5′‐CGTACCGCTTCCTTAGAACTT‐3′ and 5′‐GAATGTCATCAAGGAGAAATA‐3′, respectively. The sequence for UFSP1 #1 is 5′‐AGCCTATGTCCTGGTATTGGA‐3′. The sequence for UFSP2 #1 is 5′‐GCTGAAGACCTGCAAGTTATT‐3′.

For lentivirus production and transduction, HEK293T cells were co‐transfected with the shRNA‐expressing plasmid, psPAX3, and pMD2.G at a ratio of 4:3:1 using polyethylenimine (PEI) in Opti‐MEM medium. After 12 h, the medium was replaced with fresh culture medium. Viral supernatants were collected at 48 and 72 h post‐transfection, pooled, and filtered. For infection, cells were subjected to two rounds of lentiviral transduction. On day 1, the viral supernatant was added to cells in the presence of 8 µg/mL polybrene. After 12 h, the medium was replaced with fresh culture medium. On day 2, a second round of transduction was performed under the same conditions. On day 3, cells were cultured in fresh medium containing 1 µg/mL puromycin. Cells were then selected for 7 days to establish stable knockdown cell lines. For double knockdown experiments, the puromycin‐resistant cells were infected with lentivirus carrying the ENO1 shRNA cloned into the pLKO.1‐hygro vector, following the transduction protocol described above. After infection, cells were selected with 100 µg/mL hygromycin. Knockdown efficiency was confirmed by Western blotting analysis. For rescue experiments, ENO1 shRNA #1 targeting the endogenous ENO1 3′‐UTR was used exclusively. The exogenous Flag‐ENO1 (WT or 2KR) constructs contained only the ENO1 coding sequence without the endogenous 3′‐UTR, rendering them resistant to shRNA #1‐mediated knockdown.

### 4.5 Cell Proliferation Assay

Cells were seeded in a 6‐well plate. At the indicated time, cells were trypsinized with 0.25% trypsin and centrifuged. Cell pellets were washed and re‐suspended in PBS before counting under the microscope.

### 4.6 Cell Colony Formation Assay

Cells were plated in 6‐well plates at 1000 cells per well and cultured for the indicated time. After fixation and staining with 1% crystal violet, colonies were counted using ImageJ software.

### 4.7 Glutathione S‐Transferase (GST) Pull‐Down Assay

Recombinant GST, GST‐ENO1, and His‐UFL1 proteins were expressed in BL21(DE3) Escherichia coli. GST and GST‐ENO1 proteins were purified using Pierce Glutathione agarose (Thermo Scientific). Purified GST or GST‐ENO1 protein was incubated with His‐UFL1 for 4 h at 4°C. Beads were washed four times with NETN buffer followed by Western blotting.

### 4.8 Proximity‐Dependent Biotinylation and Streptavidin Pulldown Assay

For proximity labeling experiments, cells were transiently transfected with plasmids encoding TurboID‐EV or TurboID fused to UFL1 (TurboID‐UFL1). At 24–36 h post‐transfection, cells were incubated with biotin (50 µM) for 30 min at 37°C to induce proximity‐dependent biotinylation. Where indicated, biotin‐free medium was used as a negative control. Following treatment, cells were washed three times with PBS supplemented with 50 mM Tris to quench residual biotin and lysed in RIPA buffer containing protease inhibitors. Lysates were cleared by centrifugation (12,000 × g for 15 min at 4°C). For streptavidin affinity purification, equal amounts of protein lysate (1–2 mg per condition) were incubated with pre‐washed streptavidin magnetic beads at 4°C for 4 h with gentle rotation. Beads were subsequently washed extensively with high‐salt wash buffer (500 mM NaCl), urea wash buffer (2 M urea), and PBS to minimize non‐specific binding. Biotinylated proteins were boiled in 2× SDS loading buffer. For western blot analysis, both input lysates and streptavidin‐enriched fractions were resolved by SDS‐PAGE and transferred to PVDF membranes. Membranes were probed with antibodies against Flag, ENO1, or other indicated proteins. Global biotinylation patterns were assessed using streptavidin‐HRP. Actin was used as a loading control for input samples.

### 4.9 Lactate Production Assay

Lactate production was assessed with a Lactic Acid Assay Kit (Nanjing Jiancheng Bioengineering Institute, Nanjing, China) according to the manufacturer's instructions.

For intracellular lactate production, cells were seeded into 6‐well plates and incubated at 37°C for 24 h. After incubation, the culture medium was replaced with serum‐free, phenol red‐free RPMI medium for 1 h. Lactate levels in the medium were measured using the Lactic Acid Assay Kit. Relative lactate concentrations were normalized to cell number.

For lactate production in tissues, equal amounts of tumor tissues from each group were homogenized in ice‐cold saline at a ratio of 1:9 (w/v). The homogenates were then centrifuged at 4000 rpm for 10 min, and the supernatants were collected for lactate measurement.

### 4.10 Seahorse Assay

An XF‐96 Extracellular Flux Analyzer (Seahorse Bioscience, Agilent Technologies, CA, USA) was used to assess ECAR as described previously [50]. Briefly, 6×10^4^ cells were seeded into an XF96 plate and incubated at 37°C overnight. The next day, the culture medium was replaced with XF base medium (pH 7.4) supplemented with 4 mM glutamine, followed by a 1 h incubation. Sequential injections of glucose (10 mM), oligomycin (1.5 µM), and 2‐DG (50 mM) were performed to evaluate ECAR. Data were normalized to total protein content.

### 4.11 ENO1 Activity Assay

ENO1 activity was determined by the pyruvate kinase/L‐lactate dehydrogenase coupled reaction as described previously [20]. Briefly, 10 µg of cell lysate or 1 µg of recombinant protein was incubated in a reaction buffer containing 0.15 mM NADH, 50 mM Tris–HCl (pH 7.5), 25 mM MgSO_4_, 100 mM KCl, 1.3 mM ADP, 1.9 mM 2‐phosphoglycerate (2‐PG), 0.5 U of pyruvate kinase 2 (PKM2, Sigma, USA), and 0.67 U of lactate dehydrogenase A (LDHA, Sigma, USA). NADH oxidation was monitored by measuring the decrease in absorbance at 340 nm for 10 min.

### 4.12 Quantitative Real‐Time PCR (qRT‐PCR)

Total RNA was isolated using TRIzol reagent (Invitrogen, 15596018). One microgram of total RNA was reverse transcribed into cDNA using the FastKing gDNA Dispelling RT SuperMix (TIANGEN, KR128). Real‐time quantitative PCR was performed using the FastFire qPCR PreMix (TIANGEN, FP207‐02). The relative *c‐Myc* mRNA levels were normalized to *18S rRNA* levels. Primer sequences were as follows: *18S rRNA*: Forward: 5′‐ CGGCTACCACATCCAAGGAA ‐3′ and Reverse: 5′‐ GCTGGAATTACCGCGGCT‐3′; *c‐Myc*: Forward: 5′‐ TCGGAAGGACTATCCTGCTG‐3′, Reverse: 5′‐GTGTGTTCGCCTCTTGACATT‐3′.

### 4.13 Chemiluminescence‐Based Protein–Protein Interaction Assay

The FDA‐approved compounds and chemical libraries from the College of Pharmacy, Jinan University contained 2040 compounds supplied in 96‐well plates. HCT116 cells stably expressing Flag‐UFL1 (1‐530 aa) were generated, and cell lysates were collected. Purification of Flag‐UFL1 1–530 protein was performed by affinity enrichment on anti‐Flag affinity gel (Sigma‐Aldrich) and subsequent elution using 3x Flag Peptide working solution (Beyotime, P9801). Purified GST‐ENO1 protein (2 µg/mL) was added into each well of GSH‐coated plates (Corning, CLS‐9018) and incubated overnight at 4°C. The plate was washed three times with ice‐cold PBST. After the addition of 2 µg/mL Flag‐UFL1 1–530 protein and each compound (10 µM) to each well, the plate was incubated at 4°C for 24 h and then washed three times with PBST. The bound Flag‐UFL1 1–530 was then incubated with an HRP‐conjugated anti‐Flag antibody for 1 h at room temperature. Subsequently, 3,3’,5,5’‐Tetramethylbenzidine (TMB; BioLegend, 421101) was added. The TMB reaction was then stopped using Stop Solution (BioLegend, 423001), and the absorbance was measured at 450 nm. The levels of ENO1‐bound Flag‐UFL1 1–530 for each compound treatment were quantified relative to the vehicle control.

### 4.14 Tandem Affinity Purification and Mass Spectrometry Analyses

In brief, HCT116 cells stably expressing Flag‐UFL1 were harvested by scraping into ice‐cold PBS, pelleted at 800 × g for 5 min at 4 °C, and resuspended in NETN lysis buffer (20 mM Tris‐HCl, pH 8.0, 300 mM NaCl, and 1 mM ethylenediaminetetraacetic acid (EDTA), 0.5% NP‐40) supplemented with protease inhibitor (Roche), 10 mM β‐glycerophosphate, 1 mM sodium orthovanadate, 10 mM sodium fluoride and 1 mM phenylmethylsulfonyl fluoride (PMSF). After clearing the lysate, the supernatant was incubated with 20 µL of anti‐Flag Affinity Gel (Sigma‐Aldrich) for 2 h at 4 °C. The beads were then washed three times with cold NETN buffer. Bound proteins were released by adding 100 µL of 3×FLAG peptide solution (100 µg/mL, Sigma‐Aldrich, F4799) and incubating for 2 h at 4 °C. This elution was performed three additional times. All eluates were pooled and brought up to volume with NETN buffer. The final immunoprecipitates were resuspended in 500 µL of 50 mM ammonium bicarbonate, then sequentially incubated with 25 µL of 200 mM DTT for 30 min at 37°C and with 25 µL of 400 mM IAA for 30 min at room temperature in the dark. The sample was diluted with 500 µL of ammonium bicarbonate and digested overnight at 37°C with 5 µg of trypsin. The peptides were desalted on a C18 column and dried.

The samples were then analyzed using a Neo‐nano UPLC system coupled to an Orbitrap Ascend (Thermo Fisher Scientific). For each sample, approximately 1 µg of peptide digest was loaded per injection, and three consecutive injections were performed. The peptides were first trapped on a trap column (75 µm × 2 cm, 3 µm, 100 Å, 164535) and then separated on an Acclaim PepMapC18 RSLC analytical column (75 µm ID × 25 cm) installed in an EASY‐Spray source. Peptides were separated with a 60‐min gradient (buffer A: 0.1% formic acid in deionized water; buffer B: 0.1% formic acid in 80% acetonitrile with a flow rate of 0.3 µL/min: 45 min of 4%–25% B, 7 min of 25%–35% B, 1 min of 35%–99% B, 7 min of 99% B). The eluate was introduced directly into an Orbitrap Ascend mass spectrometer operating in data‐dependent acquisition mode. Electrospray ionization was performed with a spray voltage of 2.1 kV and the ion transfer tube held at 320 °C. The instrument switched automatically between full MS and MS/MS scans, applying a dynamic exclusion of 30 s. Mass spectra were acquired at a resolution of 60 000 with a target value of 3 × 106 ions or a maximum integration time of 50 ms. The scan range was limited from 350 to 1500 m/z. Peptides were fragmented using higher‐energy collision dissociation (HCD) at a normalized collision energy of 30%. The resulting MS^2^ spectra were collected in the Orbitrap at a resolution of 15,000, with an AGC target of 2.5 × 104, a maximum injection time of 27 ms, an isolation window of 1.6 m/z, and a fixed first m/z of 110. The dynamic exclusion duration was set to 30 s. This workflow was applied to each of the three injections per sample.

### 4.15 Database Search

The raw mass spectrometry files were processed with Proteome Discoverer following the standard workflow. The resulting mass spectrum data were analyzed quantitatively using Perseus software. Mass accuracy was set to 4.5 ppm for MS scans and 20 ppm for MS/MS scans. Spectra were searched against the human canonical UniProt FASTA database (20,395 entries, version 20210516), employing a reversed sequence decoy database to estimate the false discovery rate (FDR). Up to two tryptic missed cleavages were allowed, and compound modifications were included as variable modifications. The FDR of both peptide identification and protein identification was set to 1%. The “second peptide,” “match between runs,” and “dependent peptide” options were activated.

### 4.16 Immunofluorescence Staining

Cells were seeded onto 18 mm coverslips in 6‐well plates and maintained for 24 h before fixation with 4% paraformaldehyde for 10 min at room temperature. Fixed cells were permeabilized using 0.1% Triton X‐100 for 5 min and subsequently blocked with 5% BSA for 1 h. The samples were then incubated overnight at 4°C with anti‐ENO1 (67187‐1‐Ig, Proteintech, 1:100) and anti‐UFL1 (A303‐456A, Bethyl, 1:30). After washing with PBS, cells were incubated with secondary antibodies (Donkey anti‐Mouse IgG Alexa Fluor 488, A‐21202, 1:100; Donkey anti‐Rabbit IgG Alexa Fluor 594, A‐21207, 1:100) for 1 h at room temperature in the dark. Nuclear staining was performed with DAPI (10236276001, Sigma‐Aldrich, 1:500) for 5 min, followed by mounting coverslips onto glass slides. Cells were observed using a ZEISS Axio Observer Z1 for fluorescence microscopy. All raw microscopy images associated with this study are available in Data S2.

### 4.17 UFMylation Assay

To evaluate in vivo UFMylation, HEK293T cells were co‐transfected with ENO1 together with UFMylation‐related components and harvested 48 h later. Cell pellets were lysed by boiling for 10 min in buffer containing 150 mM Tris‐HCl (pH 8), 5% SDS, and 30% glycerol. The lysates were then diluted 20‐fold with buffer A (50 mM Tris‐HCl pH 8, 150 mM NaCl, 0.5% NP‐40, 10 mM imidazole, 2 mM NEM, and 1× protease inhibitor cocktail) according to previously described procedures [14], followed by immunoprecipitation using the indicated antibodies. To detect endogenous UFMylation of ENO1, HCT116 and SW620 cells stably expressing the relevant plasmids were grown to ∼80% confluence, lysed by boiling in lysis buffer for 10 min, and diluted 20‐fold in buffer A. After overnight incubation with an ENO1 antibody at 4°C, the immunoprecipitated complexes were analyzed by Western blotting.

To perform in vitro UFMylation, the indicated proteins were prepared through the following procedures. The coding sequences of UBA5, UFC1, and UFM1 ΔC2 were subcloned into the pET28a vector containing an N‐terminal His tag. Recombinant proteins were expressed in BL21 (DE3) and purified through Ni‐NTA affinity chromatography. For UFL1 and UFBP1 expression, full‐length sequences were inserted into the pRSFDuet‐1 vector carrying an N‐terminal His_6_‐SUMO tag and a ULP1 cleavage site. The fusion proteins were produced in BL21 (DE3) and purified using Ni‐NTA resin. After removal of the His_6_‐SUMO tag by ULP1, a second Ni‐NTA purification step was applied to eliminate the cleaved tag and ULP1, resulting in untagged UFL1 or UFBP1. GST‐ENO1 was expressed in E. coli BL21(DE3) and purified using Pierce Glutathione agarose. In vitro UFMylation reactions were assembled by combining His‐UBA5, His‐UFC1, UFL1, UFBP1, His‐UFM1 ΔC2, and GST‐ENO1 (0.1 µM each) in a reaction buffer containing 50 mM HEPES (pH 7.5), 10 mM MgCl_2_, 5 mM ATP, and 0.05% BSA. The mixture was incubated at 30°C for 2 h. The reaction was subsequently stopped by the addition of Laemmli SDS sample buffer, followed by boiling for 10 min.

### 4.18 CRC Patient‐Derived Organoids (PDOs)

CRC pathological tissues were obtained from the First Affiliated Hospital of Jinan University for the establishment of PDOs. Written informed consent was obtained from all patients, and the study was approved by the Institutional Medical Ethics Committee of Jinan University (Approval No. JNUKY‐2022‐098). To establish PDOs, freshly resected tumor tissues were cut into approximately 1–3 mm^3^ fragments and washed with antibiotic‐containing PBS. The fragments were digested at 37°C for 30 min in Advanced DMEM/F‐12 containing 1 mg/mL collagenase I, 50 U/mL hyaluronidase, and 125 µg/mL Dispase II. The suspension was filtered, centrifuged, and resuspended in Matrigel (Corning, 356234, USA). For culture, 40 µL of the Matrigel–cell mixture was plated per well in a 48‐well plate and allowed to solidify, followed by addition of 500 µL CRC organoid medium (Mogengel, MA‐0807T001SP). The medium was changed every 2–3 days, and organoids were passaged weekly. Organoids were cryopreserved using Organoid Cryopreservation Solution (Mogengel, MB‐0818L02L). For drug treatment, PDOs were seeded as single cells in 40 µL Matrigel droplets in 48‐well plates and allowed to recover for 2–3 days to form organoids. On day 3, cells were treated with torezolid (10 µM), 5‐FU (5 µM), or their combination. After 5 days, images were taken with a light microscope, and cell viability was measured using Cell Counting‐Lite 3D reagent (Vazyme, DD1102‐01), and results were normalized to the control. To assess the effect of UFL1 depletion, PDOs were dissociated into single cells and treated with lentiviruses expressing control shRNA or UFL1 shRNAs (#1 and #2). After re‐culture, images were acquired, and cell viability was assessed. Organoids were harvested using Organoid Recovery Solution (Mogengel, MA‐0837DS001P) and subsequently subjected to Western blotting analysis.

### 4.19 Animal Studies

All procedures involving animals complied with institutional ethical standards and were approved by the Institutional Animal Care and Use Committee of Jinan University (Approval Nos. 20250331‐15, 20251128‐14 and 20260126‐14).

BALB/c‐nude mice (female, 4‐6‐week‐old) were purchased from Jicui Yaokang Biotechnology Co., Ltd. (China) and maintained under specific pathogen‐free conditions in individually ventilated cages. Given that the experiments were conducted exclusively with female mice, we cannot exclude the possibility of sex‐related bias in our findings. Mice were housed under a 12‐h light/dark cycle at controlled temperature (22–24 °C) and humidity (40%–70%), with ad‐free access to food and water throughout the study. Humane endpoints were strictly followed. Mice exhibiting distress signs (hunched posture, immobility, ruffled fur, anorexia, hypothermia <34°C, or >20% weight loss) or moribund conditions (inability to stand, agonal breathing, severe atrophy/ulceration/bleeding) were euthanized immediately. The maximum allowable subcutaneous tumor volume approved by the Committee on Animal Research and Ethics was 1500 mm^3^, and this limit was not exceeded in any experiment.

For the cell‐derived xenograft (CDX) assay, HCT116 cells (5 × 10^6^) expressing the indicated plasmids were prepared in a Matrigel/PBS suspension (1:1, v/v; total volume 200 µL). The suspension was subcutaneously administered into the flanks of female BALB/c nude mice (n = 6 per group). Tumor size was monitored at five‐day intervals using electronic calipers, and tumor volume was determined using the formula V = 4π/3 × (length/2) × (width/2)^2^. At the study endpoint, mice were humanely sacrificed, and tumors were isolated and recorded.

Patient‐derived CRC xenografts were kindly provided by Dr. Changliang Shan (Nankai University) and established as previously described [20]. Briefly, PDX tumor samples were cut into approximately 3 mm^3^ fragments and implanted subcutaneously into mice using a trocar. Once tumors reached an appropriate size, tumors were harvested, divided into equal‐volume fragments, and re‐implanted subcutaneously into female BALB/c nude mice (n = 6 per group) for subsequent experiments. Tumor size was measured every five days with electronic calipers, and tumor volume was determined using the formula V = 4π/3 × (length/2) × (width/2)^2^. For in vivo gene manipulation, lentiviral particles were generated in HEK293T cells by transfection with the indicated plasmids. Viral supernatants were collected, filtered through a 0.45 µm membrane, and concentrated using PEG 8000 precipitation. When tumors reached a volume of approximately 30 mm^3^, mice received intratumoral injection with the indicated lentivirus (1 × 10^8^ PFU/100 µL per mouse) every 48 h for a total of three injections. To assess the combinatorial effect of torezolid and 5‐FU on CRC growth, mice were treated every 2 days with vehicle, torezolid (20 mg/kg, i.g.), 5‐FU (10 mg/kg, i.p.), or their combination. The dose of torezolid used in this study was selected based on previous studies demonstrating favorable tolerability and minimal toxicity in mice [46]. Tumor growth and body weight were recorded throughout the experiment. At the experimental endpoint, mice were humanely sacrificed, and tumors were isolated and recorded.

### 4.20 Immunohistochemistry (IHC)

CRC pathological tissue sections from 60 patients were collected for immunohistochemical analysis. A subset of 10 paired fresh colorectal cancer and adjacent normal tissues from this cohort was further used for immunoblotting analysis. All specimens were obtained from the First Affiliated Hospital of Jinan University with approval from the Medical Ethics Committee of Jinan University (Approval No. JNUKY‐2023‐0062). The clinical characteristics of the samples are summarized in Table S1. Tissue sections were incubated with anti‐UFL1 (Bethyl, A303‐456A, 1:200), followed by DAB‐based visualization and hematoxylin counterstaining. Immunohistochemical staining was evaluated independently by two experienced pathologists according to both the percentage of positive tumor cells and staining intensity. The proportion of positive cells was assigned a quantity score from 0 to 4 (0, 0%; 1, 1%–10%; 2, 11%–50%; 3, 51%–80%; 4, ≥81%), while staining intensity was graded on a scale of 0–3 (0, negative; 1, weak; 2, moderate; 3, strong). The final IHC score was generated by multiplying the quantity and intensity scores, resulting in a total score ranging from 0 to 12. Based on this composite score, samples were classified as negative (0), weak (1‐4), moderate (5‐8), or strong (9–12). For Ki67 and cleaved caspase‐3 immunostaining, tumor tissues were fixed in 4% paraformaldehyde, embedded in paraffin, and sectioned into 5µm slices. The sections were subsequently deparaffinized and rehydrated, followed by incubation with anti‐Ki67 (HUABIO, HA721115, 1:2500) and anti‐cleaved caspase‐3 (Cell Signaling Technology, 9661, 1:500). All stained slices were examined and imaged using a microscope. ImageJ was used for quantitative analysis of protein expression from IHC.

### 4.21 Statistical Analysis

Image quantification was performed using ImageJ, and statistical analyses were carried out using GraphPad Prism 10.1.2. The sample sizes (n) are provided in the figure legends. All in vitro experiments were independently repeated at least three times, and results are presented as mean ± SD. For animal experiments, each group contained six mice (n = 6). Comparisons between two groups were performed using a two‐tailed Student's *t*‐test, whereas multiple group comparisons were analyzed by one‐way ANOVA followed by Tukey's multiple‐comparisons test. Statistical significance was defined as ^*^ *p* < 0.05, ^**^ *p* < 0.01, and ns, not significant.

## Author Contributions

Conceptualization: XM, SHW, LS, and TL. Methodology: XM, RW, YZ, ZC, XZ, XH, RW, YZ, and LH. Software: XM, RW, ZC, YZ, and XH. Validation: XM, RW, YZ, XZ, RW, and TL. Formal analysis: XM, RW, ZC, XZ, XH, and TL. Investigation: XM, RW, YZ, and RW. Resources: XM, RW, YZ, XZ, WYS, RRH, JL, SHW, and TL. Data curation: XM and ZC. Writing – original draft: XM, RW, ZC, JL, SHW, and TL. Writing – review and editing: XM, JL, SHW, and TL. Visualization: XM, YZ, and ZC. Supervision: XM, WYS, RRH, JL, SHW, and TL. Project administration: XM, WYS, RRH, JL, SHW, and TL. Funding acquisition: XM, XZ, LS, SHW and TL.

## Ethics Statement

Human colorectal cancer specimens were obtained from the First Affiliated Hospital of Jinan University with written informed consent from all participants. PDO‐related studies were approved by the Institutional Medical Ethics Committee of Jinan University (Approval No. JNUKY‐2022‐098), and studies involving clinical specimens were approved by the Medical Ethics Committee of Jinan University (Approval No. JNUKY‐2023‐0062). All animal experiments were approved by the Institutional Animal Care and Use Committee of Jinan University (Approval Nos. 20250331‐15, 20251128‐14, and 20260126‐14).

## Conflicts of Interest

The authors declare no conflict of interest.

## Supporting information




**Supporting File 1**: advs76875‐sup‐0001‐SuppMat1.docx.

**Supporting File 2**: advs76875‐sup‐0002‐Data.zip.

## Data Availability

All data needed to evaluate the conclusions in the paper are present in the paper and/or the Supplementary Materials. The immunoprecipitation‐mass spectrometry proteomics data have been deposited in the ProteomeXchange consortium (dataset accession number: PXD078510).
