# Supplementary material for: UFL1‐Mediated UFMylation of ENO1 Restrains Aerobic Glycolysis and Colorectal Cancer Progression
Source: Adv Sci (Weinh). 2026 Jul 29:e76875. Online ahead of print. doi: 10.1002/advs.76875 (PMC13418051; doi:10.1002/advs.76875)
Supplement: Supplementary file 1 — Supporting File 1: advs76875‐sup‐0001‐SuppMat1.docx. [file ADVS-9999-e76875-s002.docx]

Supporting Information for

**UFL1-Mediated UFMylation of ENO1 Restrains Aerobic Glycolysis and Colorectal Cancer Progression**

Xiuqing Ma, Rui Wan, Yueyuan Zhong, Ziyang Cui, Xuan Zhang, Xiaoqiang He, Rong Wang, Lei Huang, Wan-Yang Sun, Rong-Rong He, Yang Zhou, Jianshuang Li, Li Shen*, Shao-Hua Wang*, Tongzheng Liu*

Corresponding authors

Tongzheng Liu: liutongzheng@jnu.edu.cn

Shao-Hua Wang: wangshh@lzu.edu.cn

Li Shen: shenli6052@sina.com

**The file includes:**

Figures S1 to S6

Tables S1 to S2

**Other supporting materials for this manuscript include the following:**

Data S1 to S3


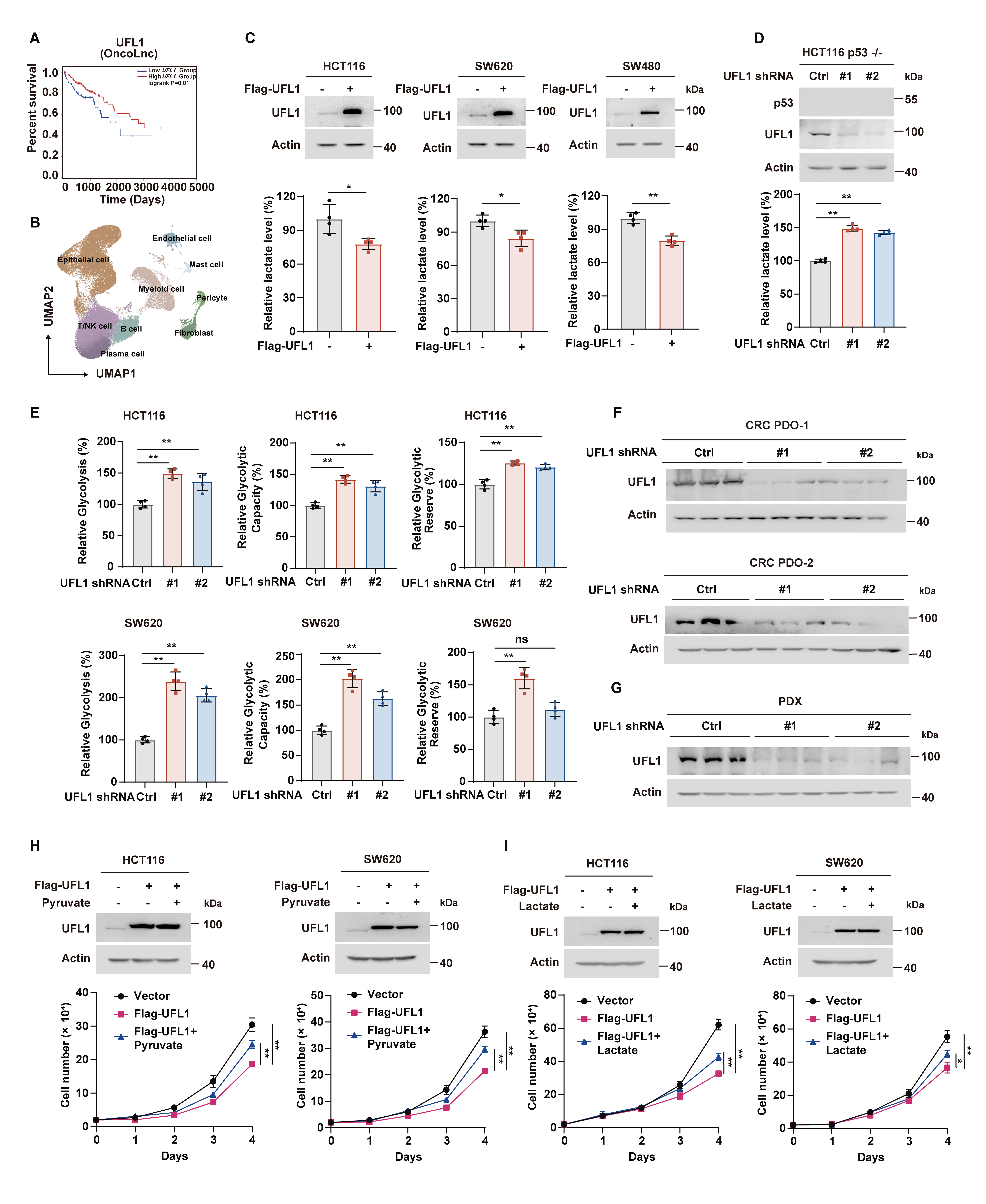


**Figure S1****. UFL1 suppresses lactate production and proliferation in CRC cells.**

(A) Kaplan-Meier survival analysis of CRC patients stratified by *UFL1* expression using OncoLnc database (http://www.oncolnc.org). (B) UMAP plot of single-cell RNA sequencing data from CRC samples. Nine major cell types were identified and annotated using established markers. (C) Lactate production assays in various CRC cells stably expressing control vector or Flag-UFL1 (n=4). (D) Lactate production assays in HCT116 p53 -/- cells stably expressing control (Ctrl), or UFL1 shRNAs (n=4). (E) Statistical analyses of ECAR measured in HCT116 and SW620 cells stably expressing control (Ctrl), or UFL1 shRNAs (n=4). (F) The efficiency of UFL1 knockdown in CRC PDO lines was analyzed by immunoblotting with the indicated antibodies. (G) Immunoblot confirming the efficiency of UFL1 knock‑down in CRC PDX tumors. (H, I) Cell proliferation assay in HCT116 and SW620 cells stably expressing control vector or Flag-UFL1, followed by treatment with 1 mM pyruvate (H) or 5 mM lactate (I) (n=3). Lysates were analyzed by immunoblotting. Error bars represent the SD. Statistical significance was determined using an unpaired Student’s t-test (C) or one-way ANOVA followed by Tukey’s multiple comparisons test (D, E, H, I); ***p* < 0.01, **p* < 0.05, ns = not significant. Raw blots are available in Data S3.

**
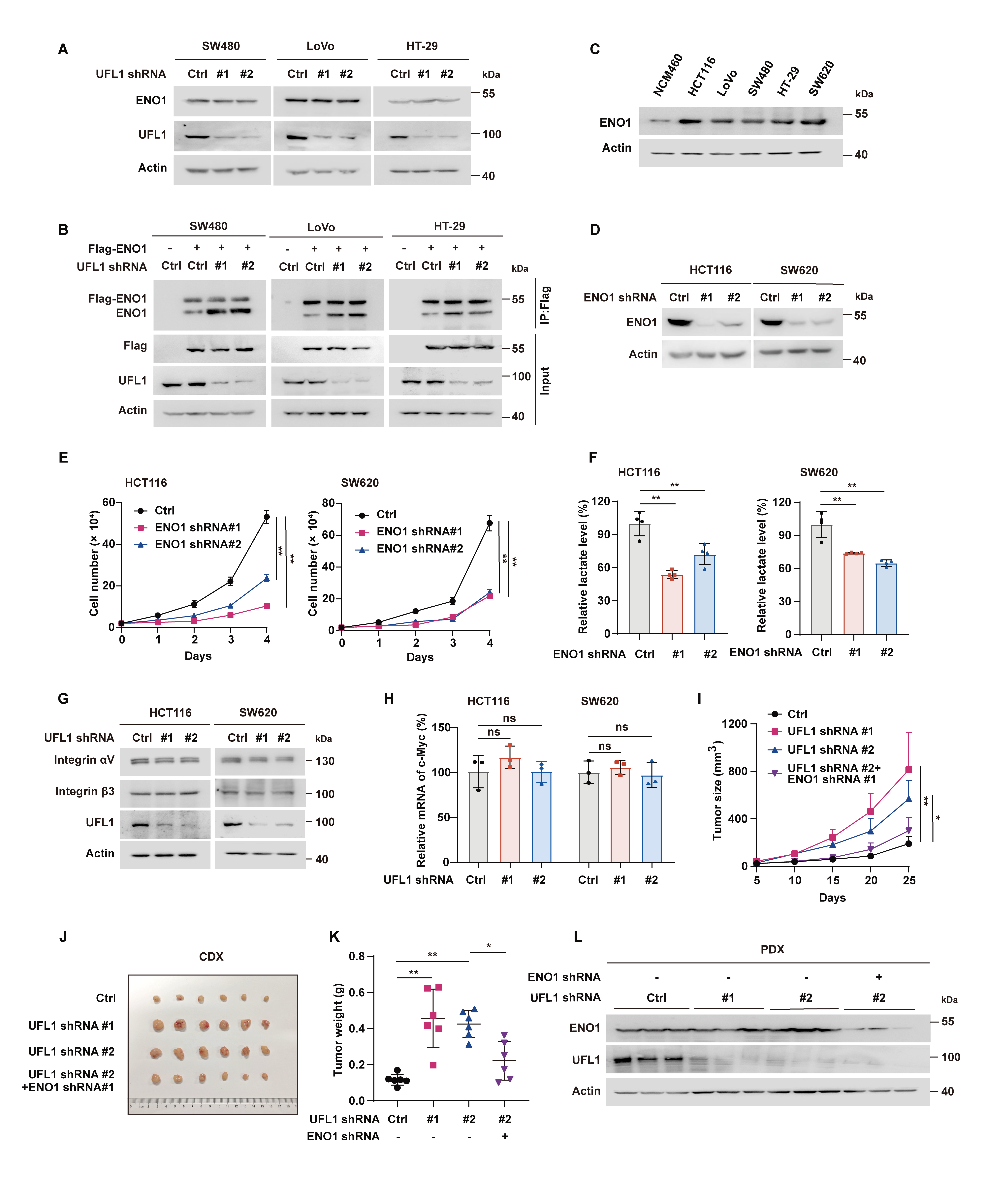
**

**Figure S2. UFL1 suppresses CRC cell proliferation through ENO1.**

(A)Various CRC cells stably expressing control (Ctrl) or UFL1 shRNAs were generated, and cell lysates were immunoblotted with the indicated antibodies. (B) CRC cells stably expressing control (Ctrl) or UFL1 shRNAs were stably transfected with Flag-tagged ENO1. ENO1 dimerization was assessed by pull-down with anti-Flag affinity gel followed by immunoblotting. (C) ENO1 expression in normal colon epithelial cells NCM460 and several CRC cell lines. (D) Western blotting analysis confirming ENO1 knockdown in HCT116 and SW620 cells. (E, F) Cell proliferation (E) (n=3) and lactate production(F) (n=4) assays in HCT116 and SW620 cells stably expressing control (Ctrl) or ENO1 shRNAs. (G) HCT116 and SW620 cells stably expressing control (Ctrl) or UFL1 shRNAs were generated, and cell lysates were immunoblotted with the indicated antibodies. (H) The expression of *c-Myc* mRNA was determined by quantitative PCR. Transcript levels were determined relative to *18S rRNA* mRNA level and normalized relative to control (n=3). (I-K) Representative tumor growth curve of xenograft tumors from HCT116 cell-derived xenografts (CDXs) in different groups (I). Analysis of tumor image (J) and tumor weight (K) (n=6). (L) Immunoblot showing simultaneous knock‑down of UFL1 and ENO1 in CRC PDX tumors from Figure 3H. Error bars represent the SD. Statistical significance was determined using one-way ANOVA followed by Tukey’s multiple comparisons test (E, F, H, I, K); ***p* < 0.01, **p* < 0.05. Raw blots are available in Data S3.


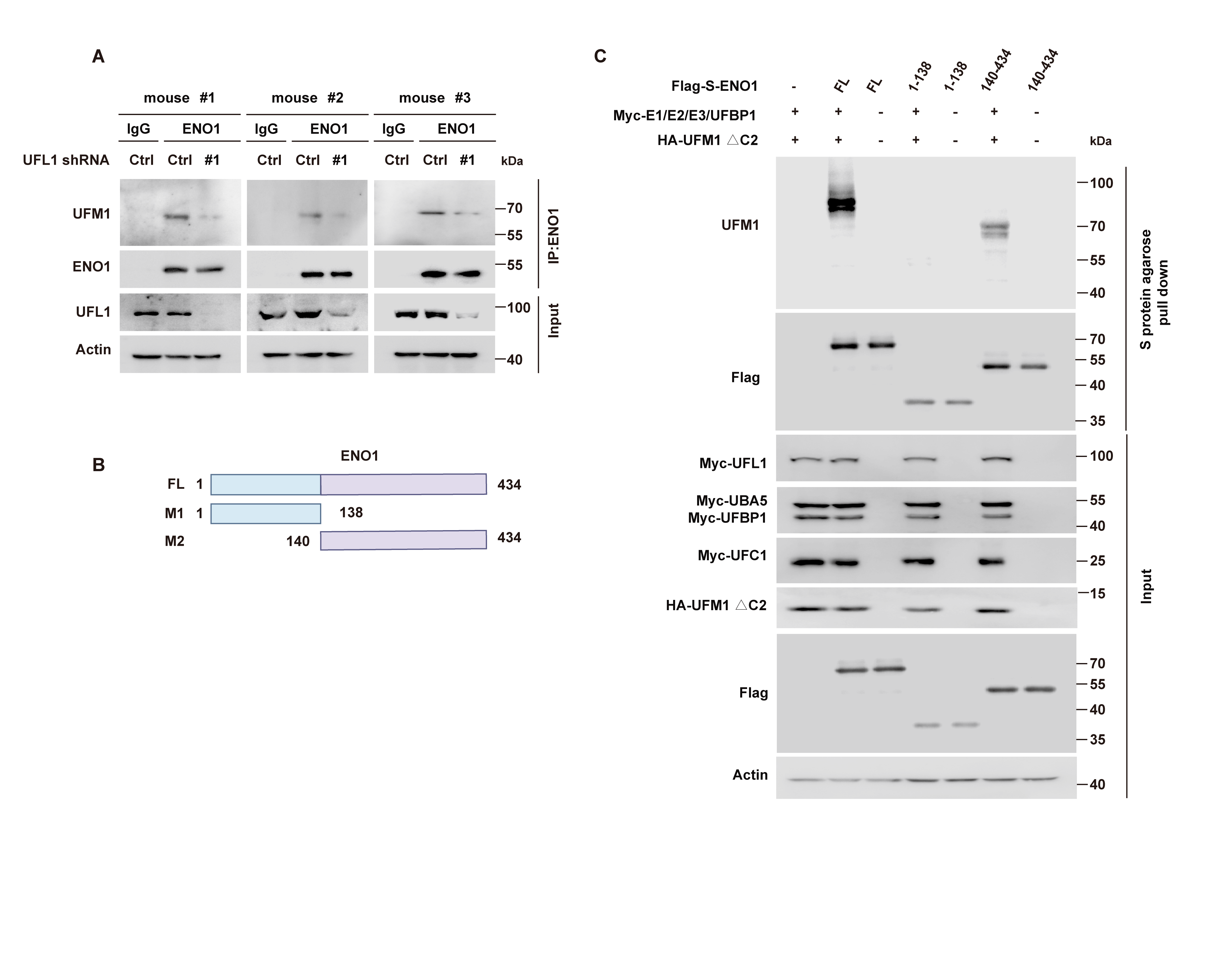


**Figure S3. ENO1 is UFMylated at C-terminal domain (140-434 aa).**

(A) Endogenous UFMylation of ENO1 was detected by immunoprecipitation using anti-ENO1 antibody, followed by immunoblotting with anti-UFM1 antibody in homogenized PDX tumors from Figure 3H. (B) Schematic diagram of ENO1 constructs, including full‐length (FL), M1 (1‐138 aa), and M2 (140‐434 aa) truncations. (C) *In vivo* UFMylation assay comparing Flag-S-ENO1 (FL) or its truncation mutants in HEK293T cells. Raw blots are available in Data S3.


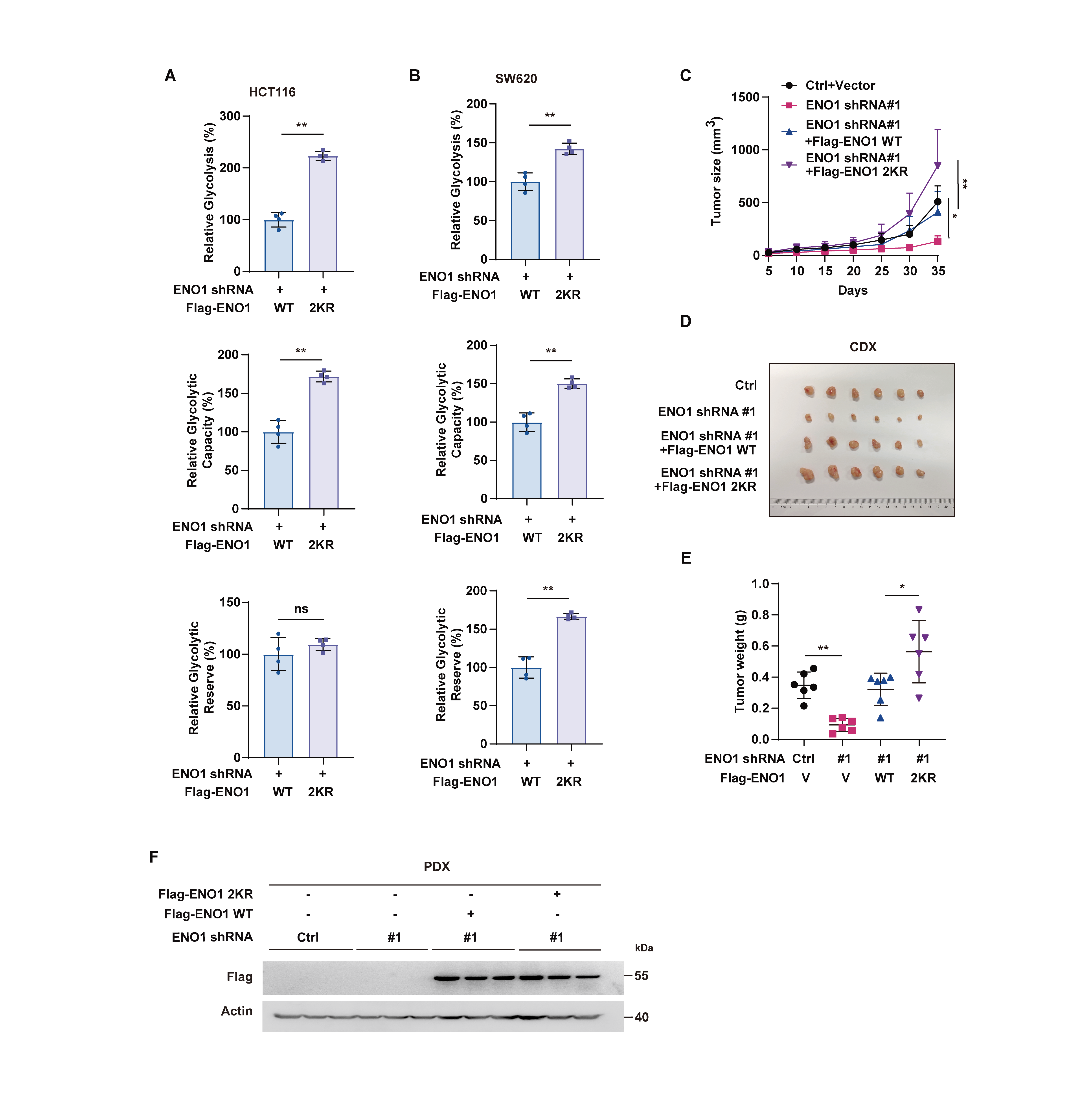


**Figure S4. UFL1-mediated UFMylation inhibits glycolytic pathway and promotes CRC progression.**

(A, B) Statistical analyses of ECAR measured in HCT116 and SW620 cells reconstituted with the indicated plasmids (n=4). (C-E) Representative tumor growth curve of xenograft tumors from HCT116 cell-derived xenografts (CDXs) in different groups(C). Analysis of tumor images (D) and tumor weight (E) (n=6). (F) Immunoblot validating exogenous ENO1 expression in PDX tumors from Figure 5H. Error bars represent the SD. Statistical significance was determined using an unpaired Student’s t-test (A, B) or one-way ANOVA followed by Tukey’s multiple comparisons test (C, E). ***p* < 0.01, **p* < 0.05, ns = not significant. Raw blots are available in Data S3.


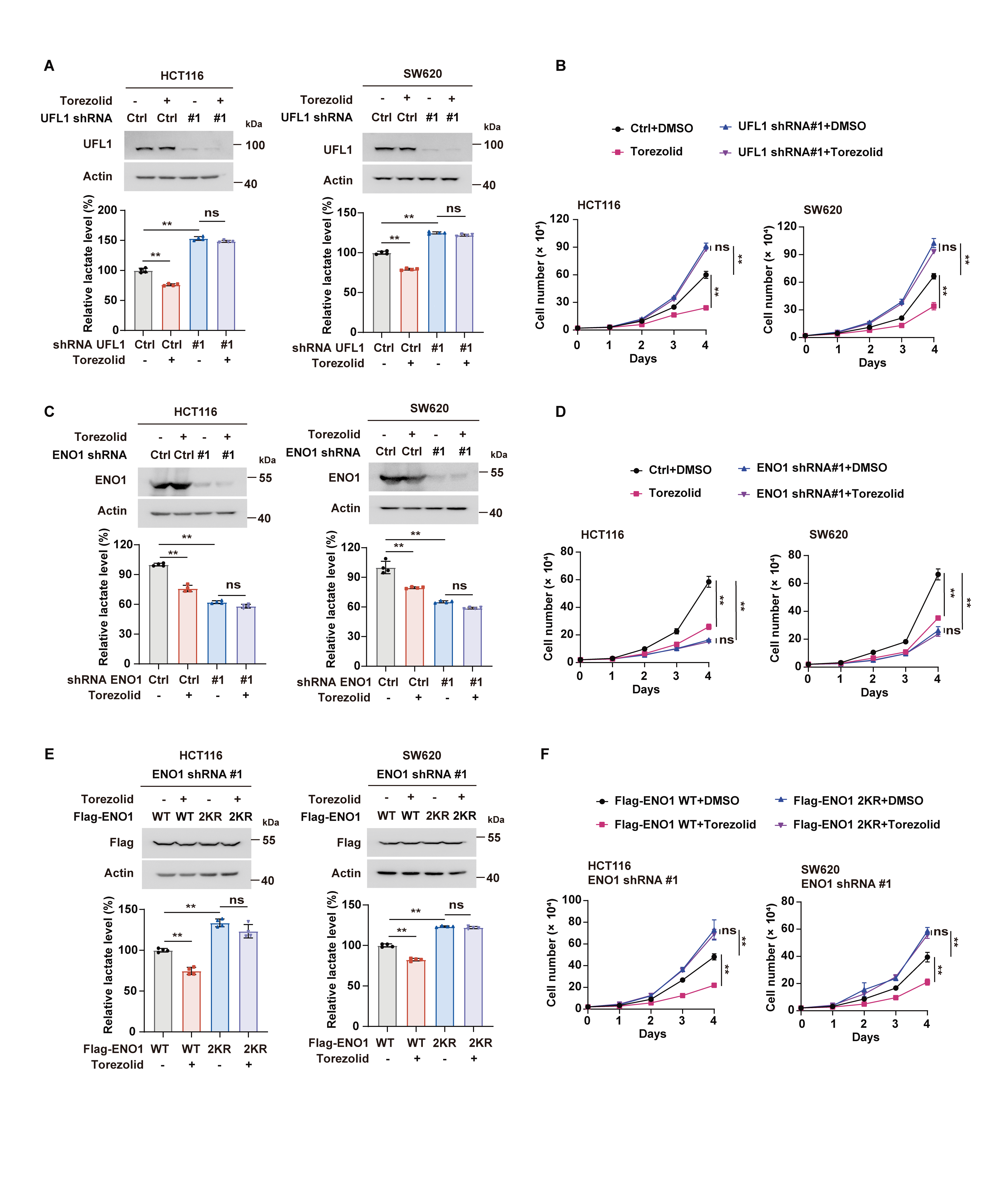


**Figure S5.** **Anti-tumor effect of torezolid is linked to ENO1 UFMyaltion mediated by UFL1.**

(A, B) Knockdown UFL1 and observe the effect of torezolid (10μM) on the cell proliferation(A) (n=4) and lactate production ability (B) (n=3) of CRC cells. (C, D) Knockdown ENO1 and observe the effect of torezolid (10μM) on the cell proliferation(C) (n=4) and lactate production ability (D) (n=3) of CRC cells. (E, F) Cell proliferation (E) (n=4) and Lactate production (F) (n=3) assays in endogenous ENO1-depleted HCT116 and SW620 cells reconstituted with the indicated plasmids, followed by treatment with DMSO or torezolid (10μM). Error bars represent the SD. Statistical significance was determined using one-way ANOVA followed by Tukey’s multiple comparisons test (A, B, C, D, E, F); ***p* < 0.01, **p* < 0.05, ns = not significant. Raw blots are available in Data S3.

**
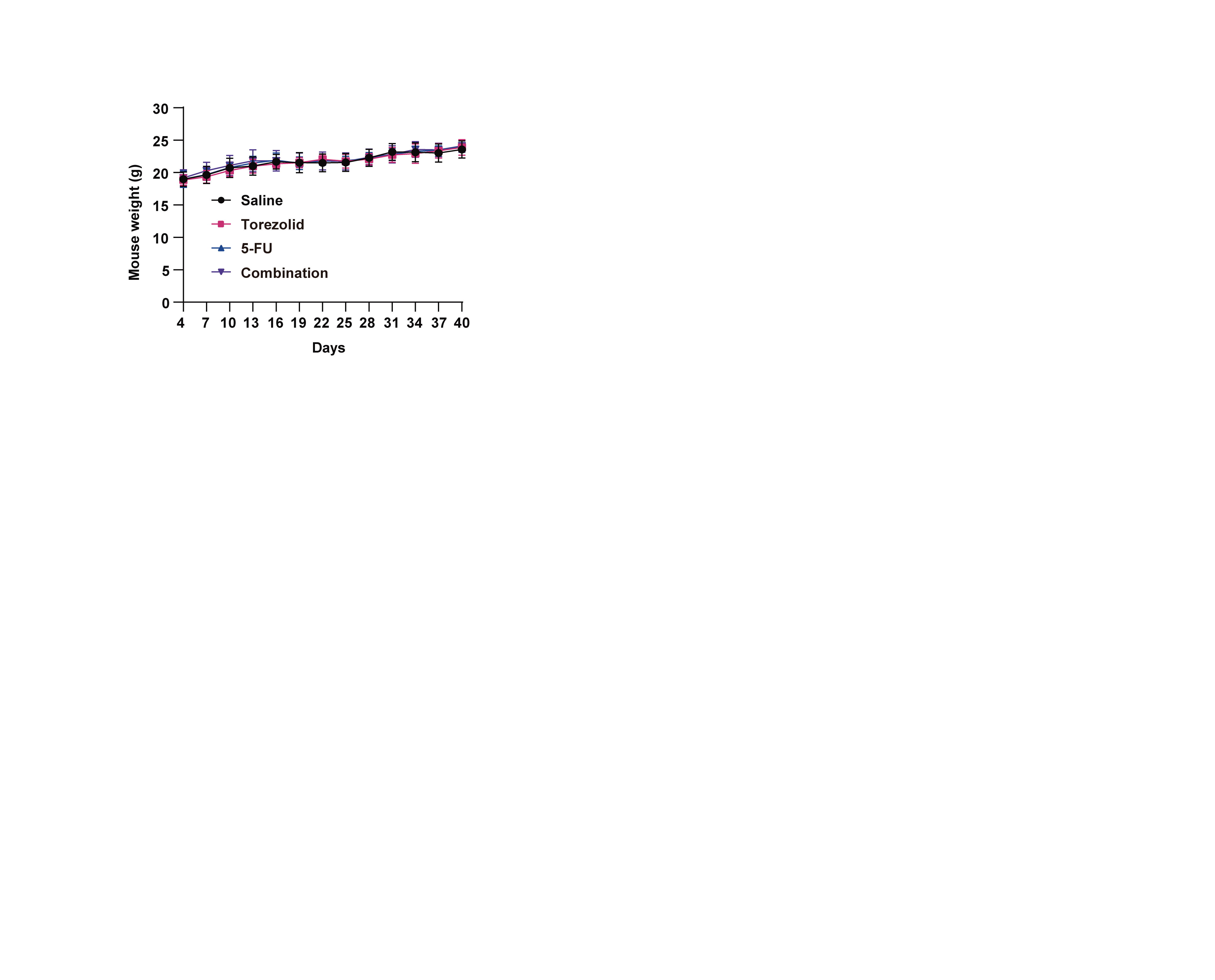
**

**Figure S6.**  **Representative mouse weight curve of xenograft tumors from CRC patient-derived xenografts (PDXs) in Fig.6l.**

**Table S1. The relationship between UFL1 expression and clinicopathological characteristics of colorectal carcinoma (CRC).**

| Clinical Character | Number | UFL1 expression | | **χ^2^** | P Value |
| --- | --- | --- | --- | --- | --- |
|  |  | Low (<4) | High (≥4) |  |  |
| Gender |  |  |  |  |  |
| Male | 29 | 15 (51.7%) | 14 (48.3%) | 0.577 | 0.448 |
| Female | 31 | 13 (41.9%) | 18 (58.1%) |  |  |
| Age (y) |  |  |  |  |  |
| <60 | 30 | 15 (50.0%) | 15 (50.0%) | 0.268 | 0.605 |
| ≥60 | 30 | 13 (43.3%) | 17 (56.7%) |  |  |
| Size |  |  |  |  |  |
| <5cm | 45 | 20 (44.4%) | 25 (55.6%) | 0.357 | 0.550 |
| ≥5cm | 15 | 8 (53.3%) | 7 (46.7%) |  |  |
| Location |  |  |  |  |  |
| Rectum | 28 | 12 (42.9%) | 16 (57.1%) | 0.306 | 0.580 |
| Colon | 32 | 16 (50.0%) | 16 (50.0%) |  |  |
| Differentiated degree |  |  |  |  |  |
| High- Mid differentiation | 48 | 26 (54.2%) | 22 (45.8%) | 5.424 | 0.020 |
| Low differentiation | 12 | 2 (16.7%) | 10 (83.3%) |  |  |
| T(Tumor)-stage |  |  |  |  |  |
| Tis-T2 | 29 | 5 (17.2%) | 24 (82.8%) | 19.526 | <0.001 |
| T3-T4 | 31 | 23 (74.2%) | 8 (25.8%) |  |  |
| N(Node)-Stage |  |  |  |  |  |
| N0 | 36 | 10 (27.8%) | 26 (72.2%) | 12.902 | <0.001 |
| N1-N2 | 24 | 18 (75.0%) | 6 (25.0%) |  |  |
| Clinical Stage |  |  |  |  |  |
| I-II | 36 | 10 (27.8%) | 26 (72.2%) | 12.902 | <0.001 |
| III-IV | 24 | 18 (75.0%) | 6 (25.0%) |  |  |

**Table S2. Sources of the single-cell RNA-seq datasets.**

| **Sample Type** | **PMID** | **GSE** | **GSM** | **YEAR** |
| --- | --- | --- | --- | --- |
| Tumor | NA | GSE302903 | GSM9113377 | 2025 |
| Tumor | NA | GSE302903 | GSM9113378 | 2025 |
| Tumor | NA | GSE302903 | GSM9113379 | 2025 |
| Tumor | NA | GSE302903 | GSM9113380 | 2025 |
| Tumor | NA | GSE302903 | GSM9113381 | 2025 |
| Tumor | NA | GSE302903 | GSM9113382 | 2025 |
| Tumor | NA | GSE302903 | GSM9113383 | 2025 |
| Tumor | NA | GSE302903 | GSM9113384 | 2025 |
| Tumor | NA | GSE302903 | GSM9113385 | 2025 |
| Tumor | NA | GSE302903 | GSM9113386 | 2025 |
| Tumor | NA | GSE302903 | GSM9113387 | 2025 |
| Tumor | NA | GSE302903 | GSM9113388 | 2025 |
| Tumor | 38335276 | GSE245552 | GSM7844812 | 2024 |
| Tumor | 38335276 | GSE245552 | GSM7844814 | 2024 |
| Tumor | 38335276 | GSE245552 | GSM7844816 | 2024 |
| Tumor | 38335276 | GSE245552 | GSM7844818 | 2024 |
| Tumor | 38335276 | GSE245552 | GSM7844820 | 2024 |
| Tumor | 38335276 | GSE245552 | GSM7844823 | 2024 |
| Tumor | 38335276 | GSE245552 | GSM7844826 | 2024 |
| Tumor | 38335276 | GSE245552 | GSM7844828 | 2024 |
| Tumor | 38335276 | GSE245552 | GSM7844829 | 2024 |
| Tumor | 38335276 | GSE245552 | GSM7844832 | 2024 |
| Tumor | 38335276 | GSE245552 | GSM7844833 | 2024 |
| Tumor | 38335276 | GSE245552 | GSM7844835 | 2024 |
| Tumor | 38335276 | GSE245552 | GSM7844837 | 2024 |
| Tumor | 38335276 | GSE245552 | GSM7844839 | 2024 |
| Tumor | 38335276 | GSE245552 | GSM7844844 | 2024 |
| Tumor | 38335276 | GSE245552 | GSM7844847 | 2024 |
| Tumor | 35538548 | GSE200997 | GSM6048346 | 2022 |
| Tumor | 35538548 | GSE200997 | GSM6048347 | 2022 |
| Tumor | 35538548 | GSE200997 | GSM6048348 | 2022 |
| Tumor | 35538548 | GSE200997 | GSM6048349 | 2022 |
| Tumor | 35538548 | GSE200997 | GSM6048350 | 2022 |
| Tumor | 35538548 | GSE200997 | GSM6048351 | 2022 |
| Tumor | 35538548 | GSE200997 | GSM6048352 | 2022 |
| Tumor | 35538548 | GSE200997 | GSM6048353 | 2022 |
| Tumor | 35538548 | GSE200997 | GSM6048354 | 2022 |
| Tumor | 35538548 | GSE200997 | GSM6048355 | 2022 |
| Tumor | 35538548 | GSE200997 | GSM6048356 | 2022 |
| Tumor | 35538548 | GSE200997 | GSM6048357 | 2022 |
| Tumor | 35538548 | GSE200997 | GSM6048358 | 2022 |
| Tumor | 35538548 | GSE200997 | GSM6048359 | 2022 |
| Tumor | 35538548 | GSE200997 | GSM6048360 | 2022 |
| Tumor | 35538548 | GSE200997 | GSM6048361 | 2022 |
| Tumor | 34793335 | GSE188711 | GSM5688706 | 2021 |
| Tumor | 34793335 | GSE188711 | GSM5688707 | 2021 |
| Tumor | 34793335 | GSE188711 | GSM5688708 | 2021 |
| Tumor | 34793335 | GSE188711 | GSM5688709 | 2021 |
| Tumor | 34793335 | GSE188711 | GSM5688710 | 2021 |
| Tumor | 34793335 | GSE188711 | GSM5688711 | 2021 |
| Tumor | 34409732 | GSE166555 | GSM5075660 | 2021 |
| Tumor | 34409732 | GSE166555 | GSM5075662 | 2021 |
| Tumor | 34409732 | GSE166555 | GSM5075665 | 2021 |
| Tumor | 34409732 | GSE166555 | GSM5075666 | 2021 |
| Tumor | 34409732 | GSE166555 | GSM5075668 | 2021 |
| Tumor | 34409732 | GSE166555 | GSM5075670 | 2021 |
| Tumor | 34409732 | GSE166555 | GSM5075672 | 2021 |
| Tumor | 34409732 | GSE166555 | GSM5075674 | 2021 |
| Tumor | 34409732 | GSE166555 | GSM5075676 | 2021 |
| Tumor | 34409732 | GSE166555 | GSM5075678 | 2021 |
| Tumor | 34409732 | GSE166555 | GSM5075680 | 2021 |
| Tumor | 34409732 | GSE166555 | GSM5075682 | 2021 |
| Tumor | 34409732 | GSE166555 | GSM5075683 | 2021 |
| Tumor | 37172580 | [GSE205506](https://www.ncbi.nlm.nih.gov/geo/query/acc.cgi?acc=GSE205506) | GSM6213970 | 2023 |
| Tumor | 37172580 | [GSE205506](https://www.ncbi.nlm.nih.gov/geo/query/acc.cgi?acc=GSE205506) | GSM6213971 | 2023 |
| Tumor | 37172580 | [GSE205506](https://www.ncbi.nlm.nih.gov/geo/query/acc.cgi?acc=GSE205506) | GSM6213973 | 2023 |
| Tumor | 37172580 | [GSE205506](https://www.ncbi.nlm.nih.gov/geo/query/acc.cgi?acc=GSE205506) | GSM6213976 | 2023 |
| Tumor | 37172580 | [GSE205506](https://www.ncbi.nlm.nih.gov/geo/query/acc.cgi?acc=GSE205506) | GSM6213980 | 2023 |
| Tumor | 37172580 | [GSE205506](https://www.ncbi.nlm.nih.gov/geo/query/acc.cgi?acc=GSE205506) | GSM6213983 | 2023 |
| Tumor | 37172580 | [GSE205506](https://www.ncbi.nlm.nih.gov/geo/query/acc.cgi?acc=GSE205506) | GSM6213988 | 2023 |
| Tumor | 37172580 | [GSE205506](https://www.ncbi.nlm.nih.gov/geo/query/acc.cgi?acc=GSE205506) | GSM6213991 | 2023 |
| Tumor | 37172580 | [GSE205506](https://www.ncbi.nlm.nih.gov/geo/query/acc.cgi?acc=GSE205506) | GSM6213994 | 2023 |
| Tumor | 37172580 | [GSE205506](https://www.ncbi.nlm.nih.gov/geo/query/acc.cgi?acc=GSE205506) | GSM6213995 | 2023 |
| Tumor | 37768068 | GSE231559 | GSM7290763 | 2023 |
| Tumor | 37768068 | GSE231559 | GSM7290769 | 2023 |
| Tumor | 37768068 | GSE231559 | GSM7290772 | 2023 |
| Tumor | 37768068 | GSE231559 | GSM7290773 | 2023 |
| Tumor | 37768068 | GSE231559 | GSM7290774 | 2023 |
| Tumor | 37768068 | GSE231559 | GSM7290777 | 2023 |
| Normal | 38335276 | GSE245552 | GSM7844838 | 2024 |
| Normal | 38335276 | GSE245552 | GSM7844841 | 2024 |
| Normal | 38335276 | GSE245552 | GSM7844846 | 2024 |
| Normal | 38335276 | GSE245552 | GSM7844849 | 2024 |
| Normal | 35538548 | GSE200997 | GSM6048362 | 2022 |
| Normal | 35538548 | GSE200997 | GSM6048363 | 2022 |
| Normal | 35538548 | GSE200997 | GSM6048364 | 2022 |
| Normal | 35538548 | GSE200997 | GSM6048365 | 2022 |
| Normal | 35538548 | GSE200997 | GSM6048366 | 2022 |
| Normal | 35538548 | GSE200997 | GSM6048367 | 2022 |
| Normal | 35538548 | GSE200997 | GSM6048368 | 2022 |
| Normal | 34409732 | GSE166555 | GSM5075659 | 2021 |
| Normal | 34409732 | GSE166555 | GSM5075661 | 2021 |
| Normal | 34409732 | GSE166555 | GSM5075663 | 2021 |
| Normal | 34409732 | GSE166555 | GSM5075664 | 2021 |
| Normal | 34409732 | GSE166555 | GSM5075667 | 2021 |
| Normal | 34409732 | GSE166555 | GSM5075669 | 2021 |
| Normal | 34409732 | GSE166555 | GSM5075671 | 2021 |
| Normal | 34409732 | GSE166555 | GSM5075673 | 2021 |
| Normal | 34409732 | GSE166555 | GSM5075675 | 2021 |
| Normal | 34409732 | GSE166555 | GSM5075677 | 2021 |
| Normal | 34409732 | GSE166555 | GSM5075679 | 2021 |
| Normal | 34409732 | GSE166555 | GSM5075681 | 2021 |
