# Supplementary material for: UFL1‐Mediated UFMylation of ENO1 Restrains Aerobic Glycolysis and Colorectal Cancer Progression
Source: Adv Sci (Weinh). 2026 Jul 29:e76875. Online ahead of print. doi: 10.1002/advs.76875 (PMC13418051; doi:10.1002/advs.76875)
Supplement: Supplementary file 2 — Supporting File 2: advs76875‐sup‐0002‐Data.zip. [file ADVS-9999-e76875-s001.zip › advs76875-sup-0002-Data/Data S2 Raw Microscopy Images.pptx]

## Slide 1
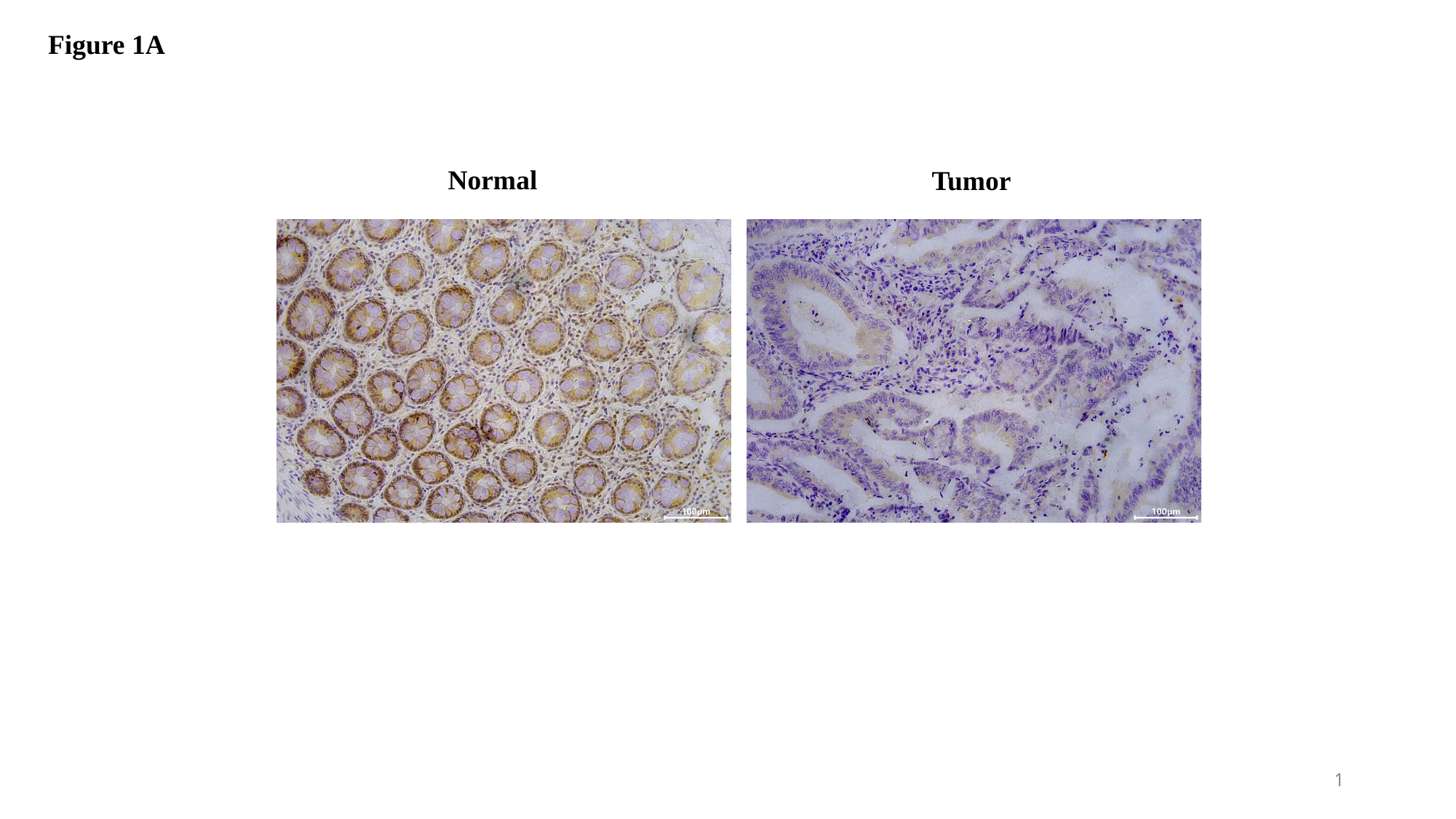

Figure 1A
Normal
Tumor
1

## Slide 2
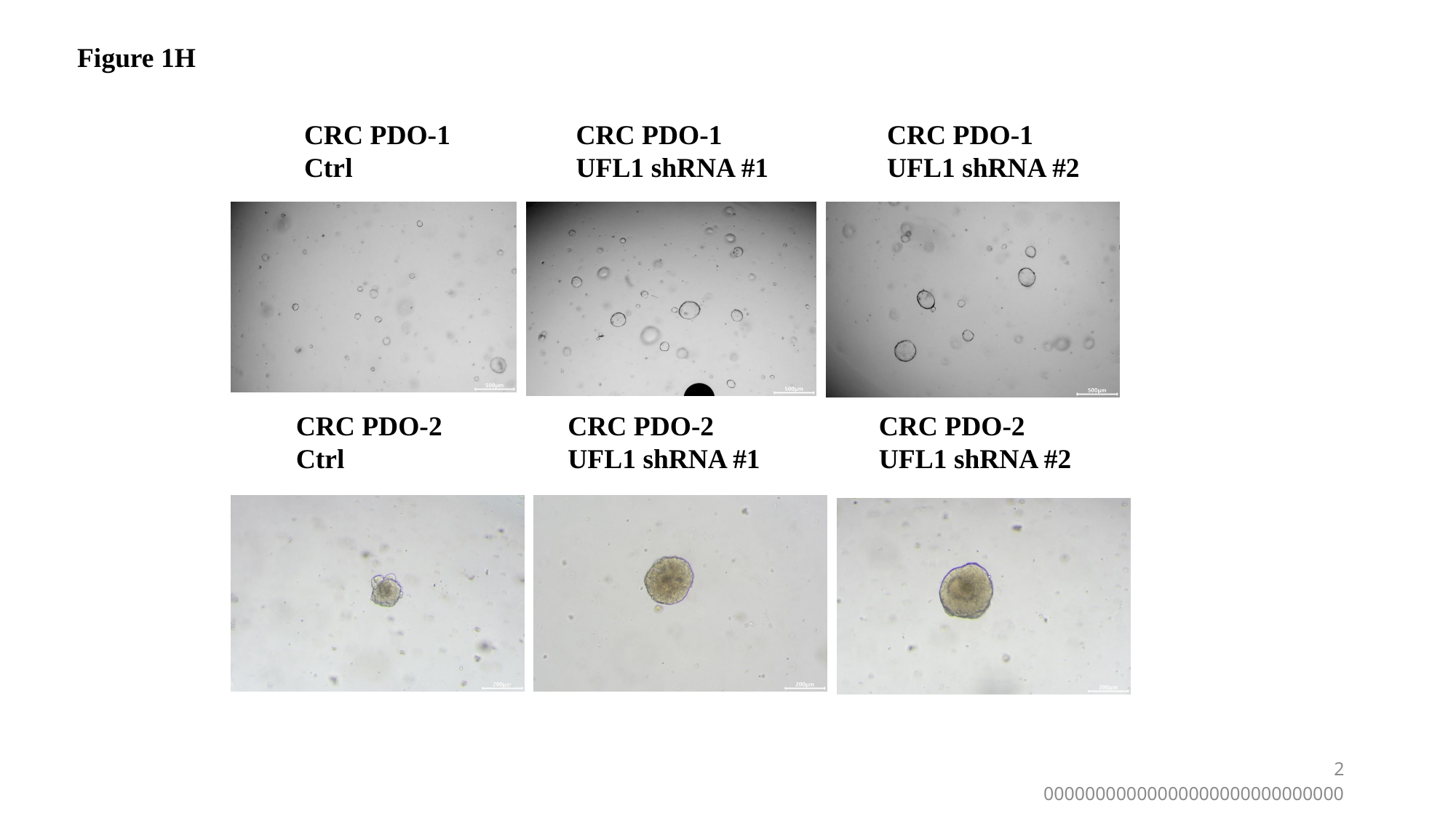

Figure 1H
CRC PDO-1
UFL1 shRNA #1
CRC PDO-1
UFL1 shRNA #2
CRC PDO-1
Ctrl
CRC PDO-2
UFL1 shRNA #1
CRC PDO-2
UFL1 shRNA #2
CRC PDO-2
Ctrl
200000000000000000000000000000

## Slide 3
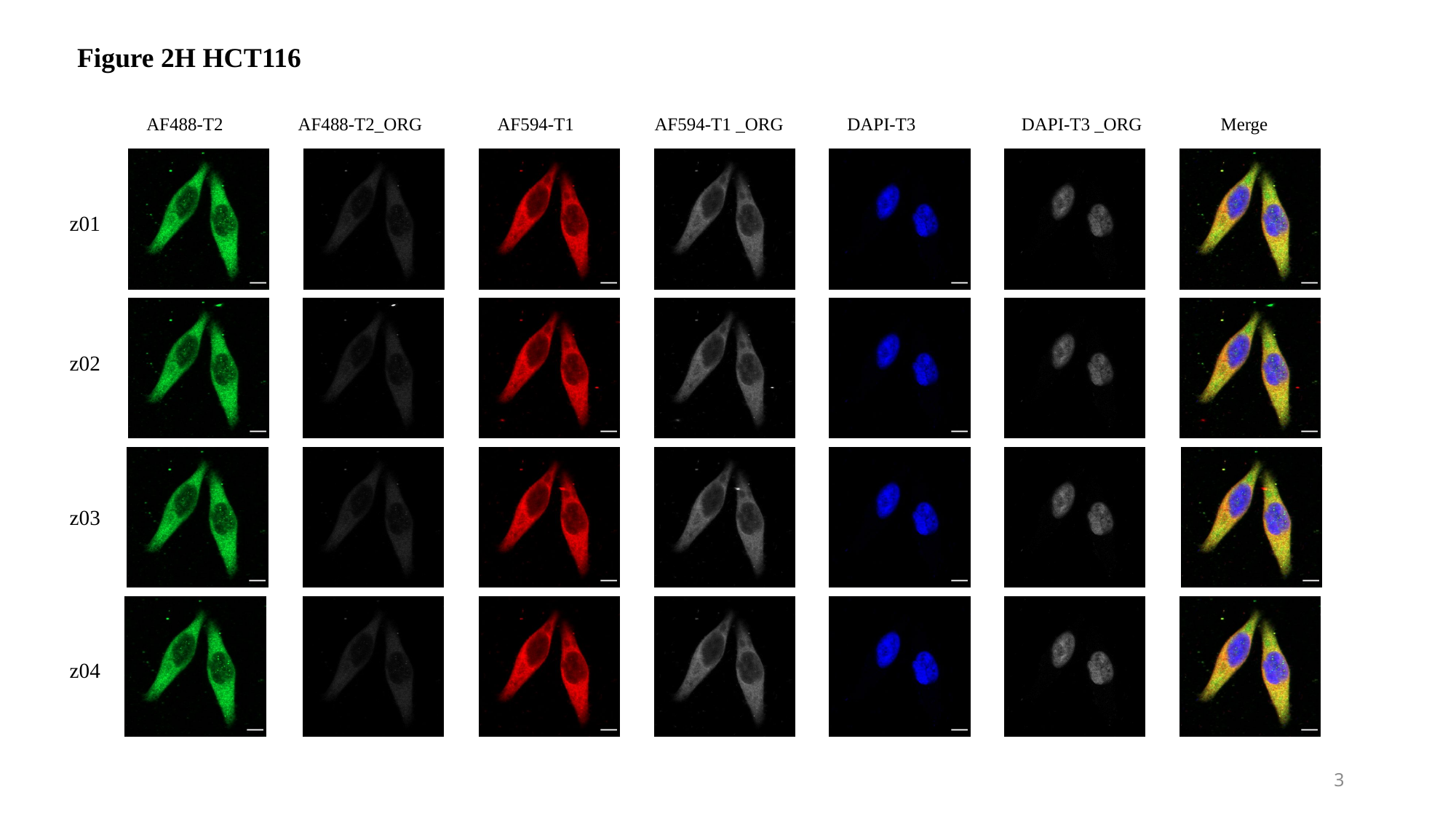

Figure 2H HCT116
AF488-T2
AF488-T2_ORG
AF594-T1
AF594-T1 _ORG
DAPI-T3
DAPI-T3 _ORG
Merge
z01
z02
z03
z04
3

## Slide 4
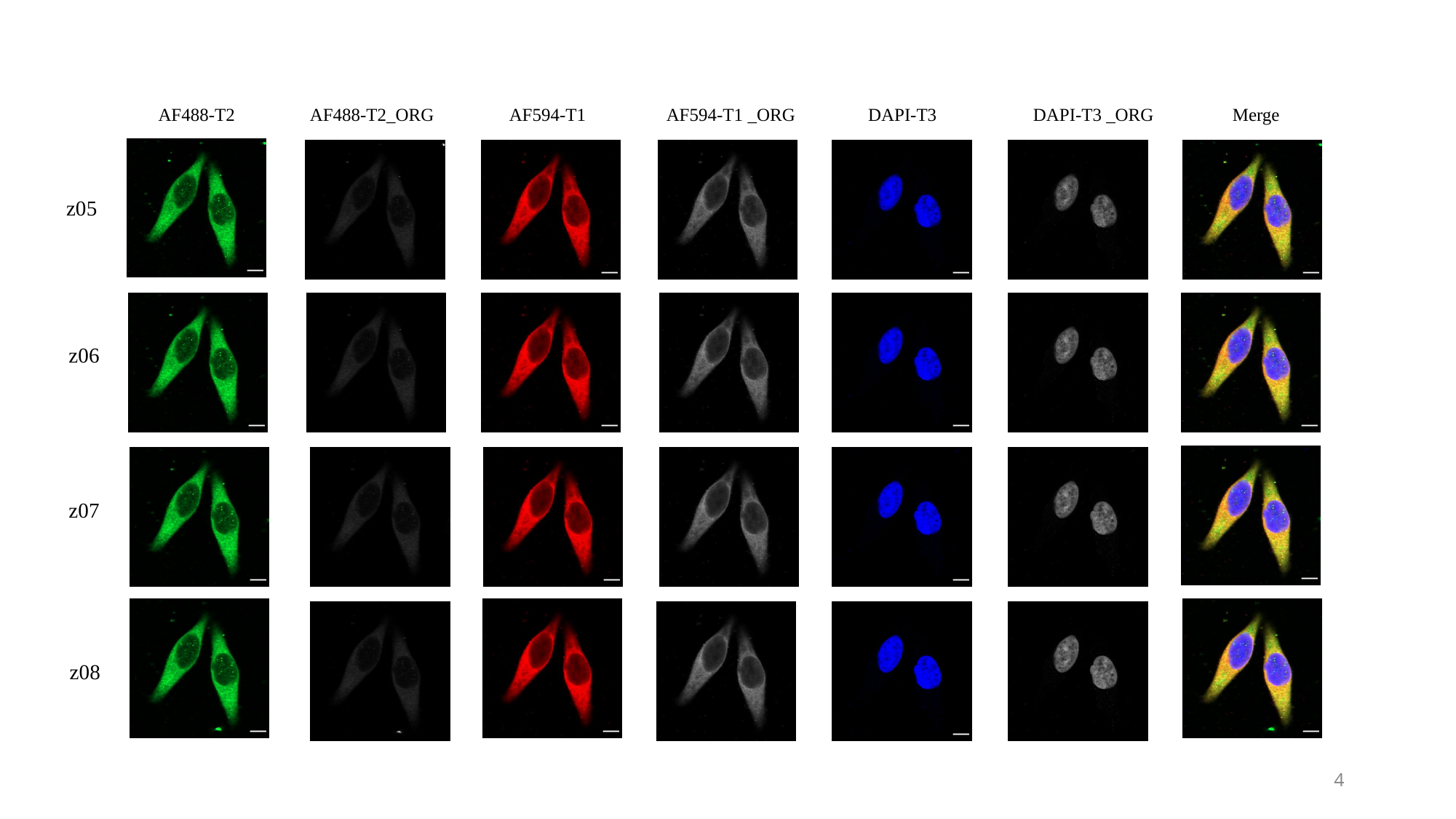

AF488-T2
AF488-T2_ORG
AF594-T1
AF594-T1 _ORG
 DAPI-T3
DAPI-T3 _ORG
Merge
z05
z06
z07
z08
4

## Slide 5
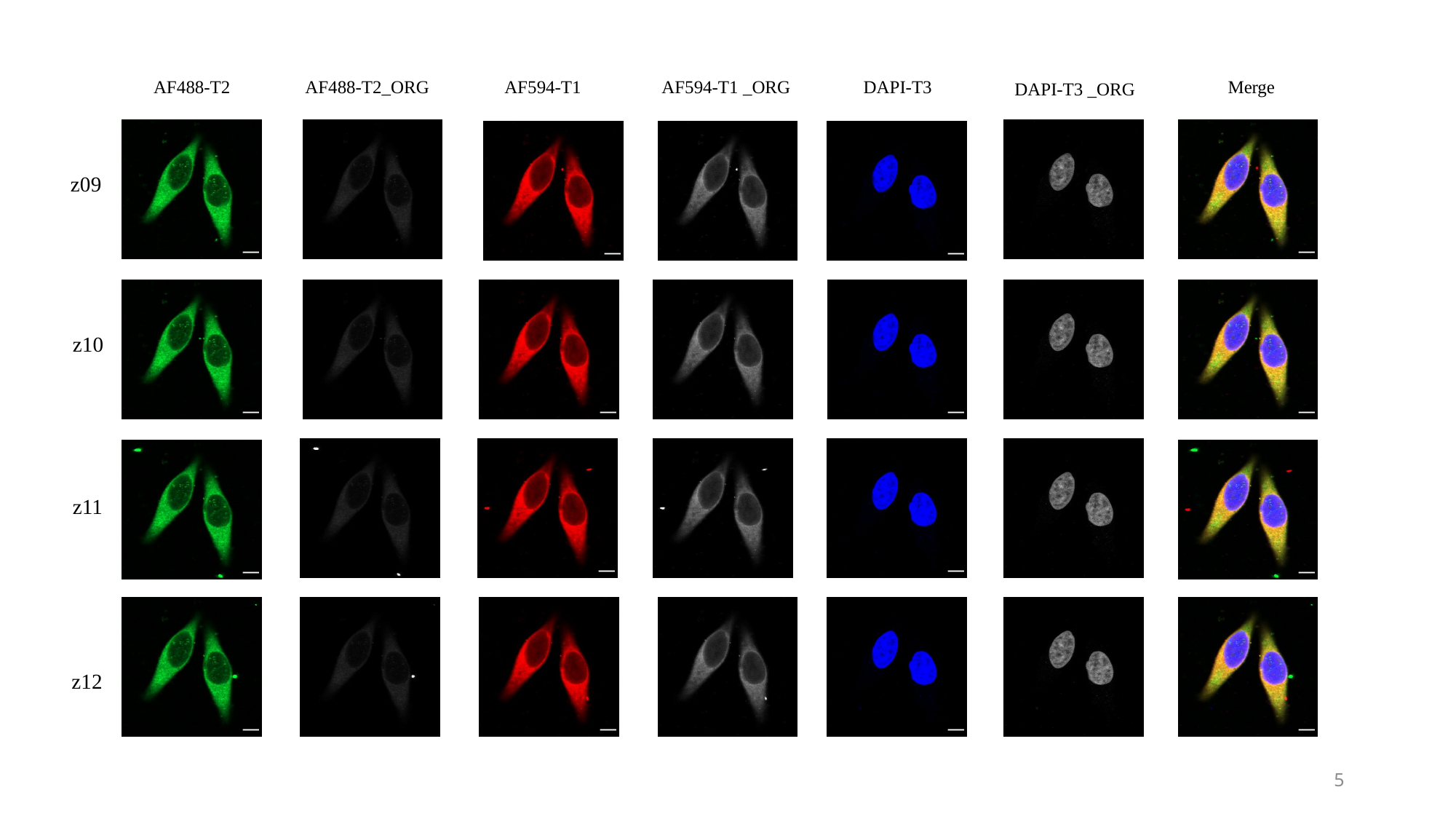

AF488-T2
AF488-T2_ORG
AF594-T1
AF594-T1 _ORG
 DAPI-T3
Merge
DAPI-T3 _ORG
z09
z10
z11
z12
5

## Slide 6
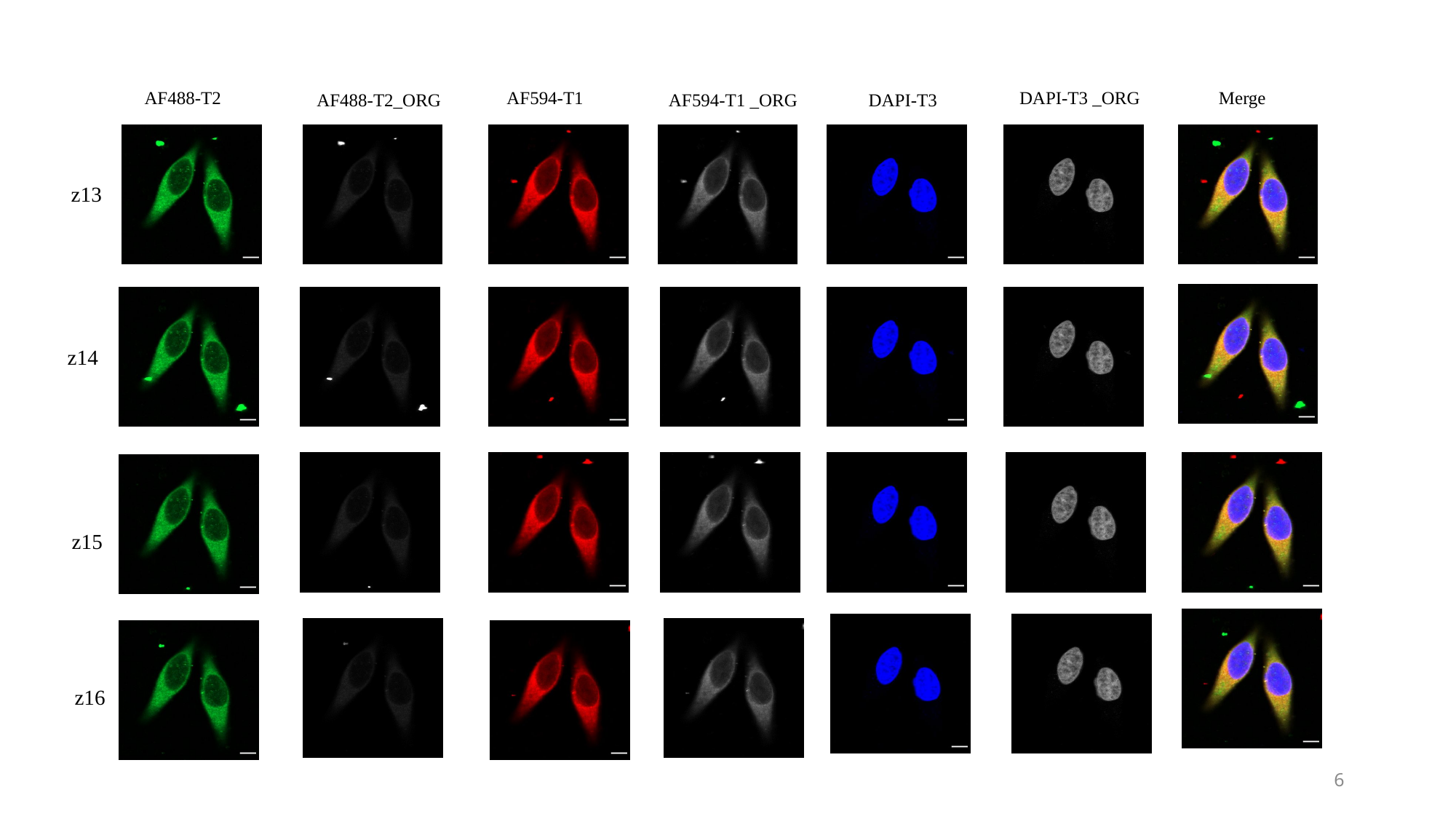

AF488-T2
AF594-T1
DAPI-T3 _ORG
Merge
AF488-T2_ORG
AF594-T1 _ORG
 DAPI-T3
z13
z14
z15
z16
6

## Slide 7
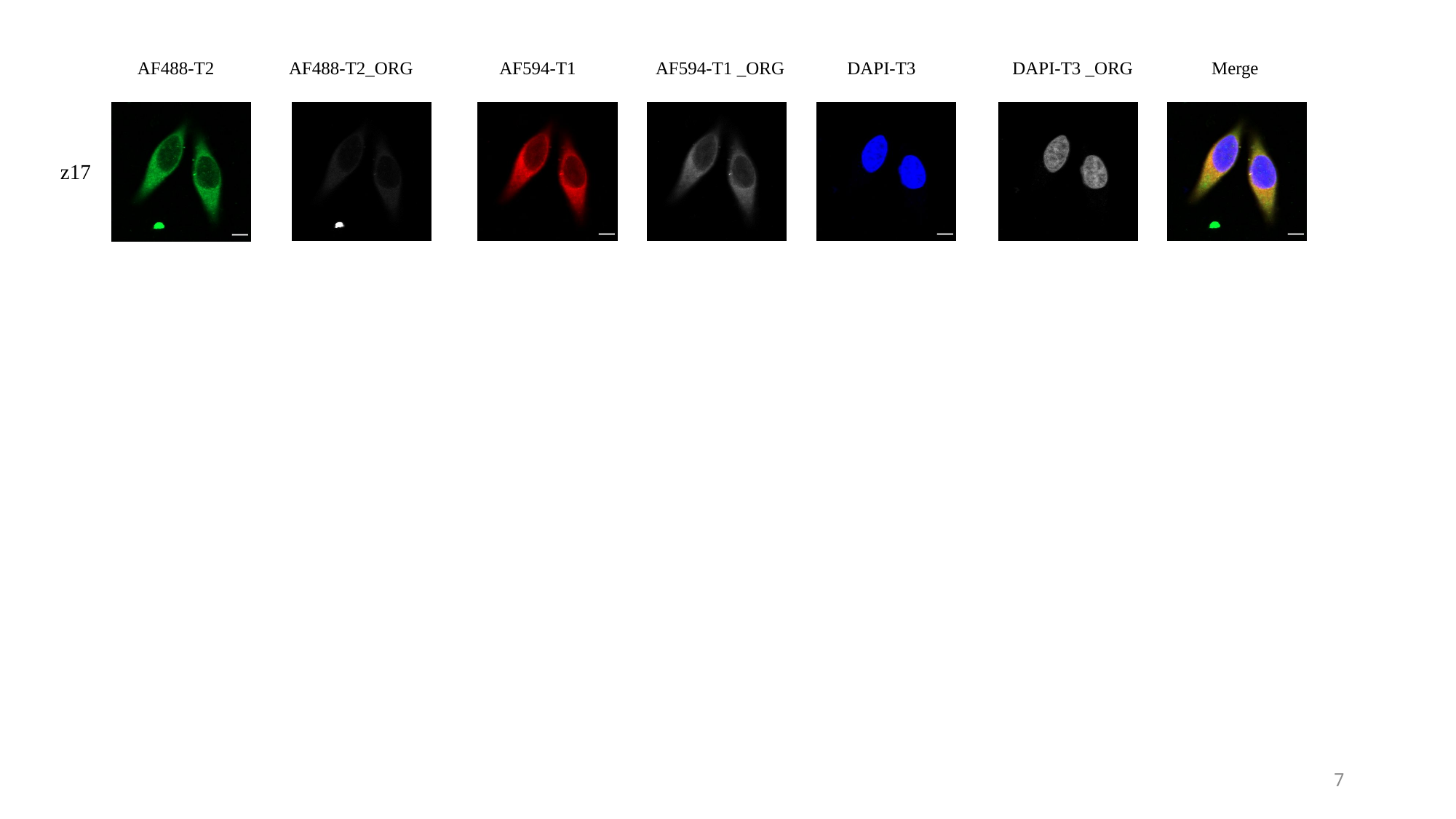

AF488-T2
AF488-T2_ORG
AF594-T1
AF594-T1 _ORG
 DAPI-T3
DAPI-T3 _ORG
Merge
z17
7

## Slide 8
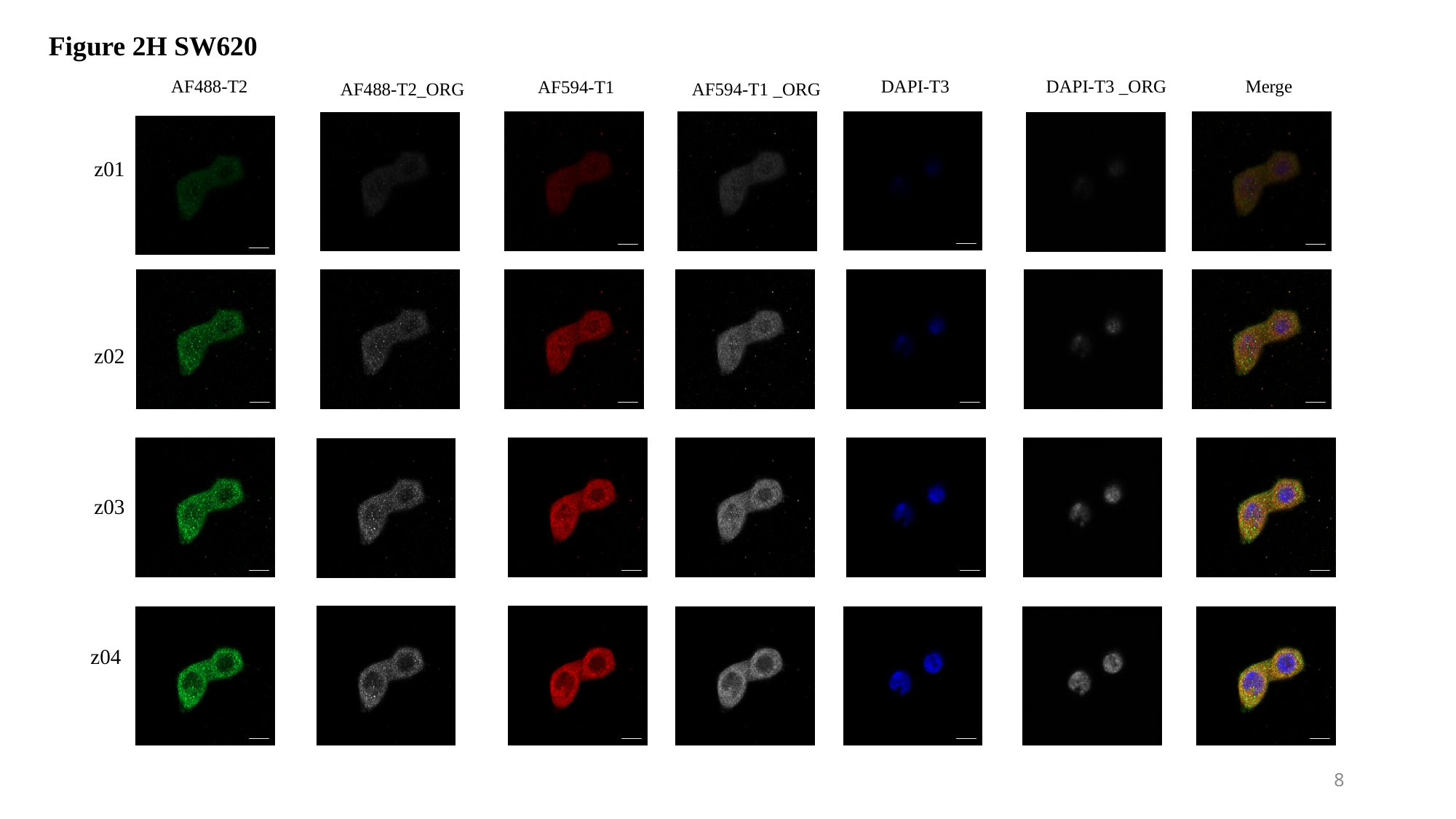

Figure 2H SW620
AF488-T2
 DAPI-T3
DAPI-T3 _ORG
Merge
AF594-T1
AF594-T1 _ORG
AF488-T2_ORG
z01
z02
z03
z04
8

## Slide 9
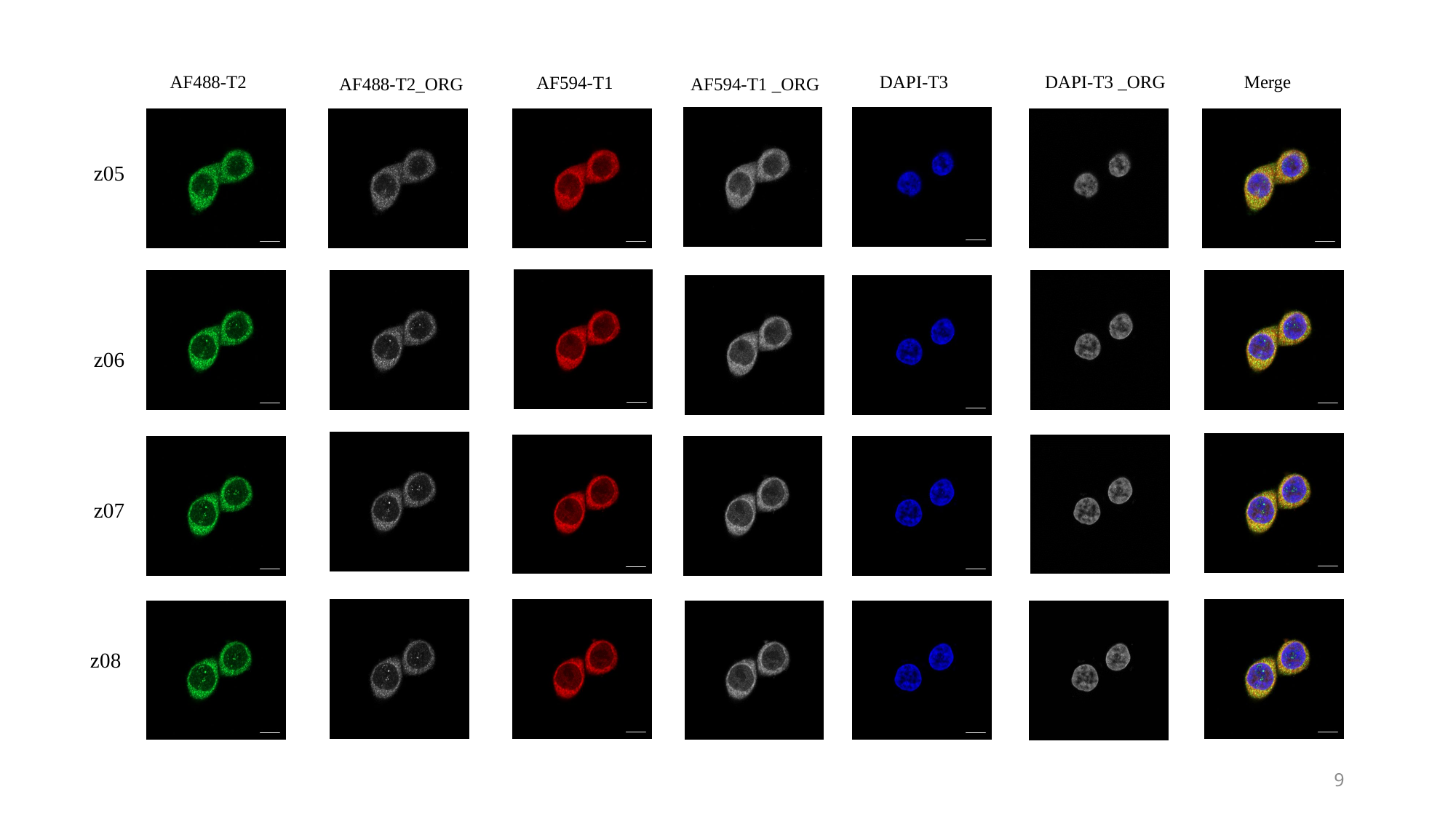

AF488-T2
 DAPI-T3
DAPI-T3 _ORG
Merge
AF594-T1
AF594-T1 _ORG
AF488-T2_ORG
z05
z06
z07
z08
9

## Slide 10
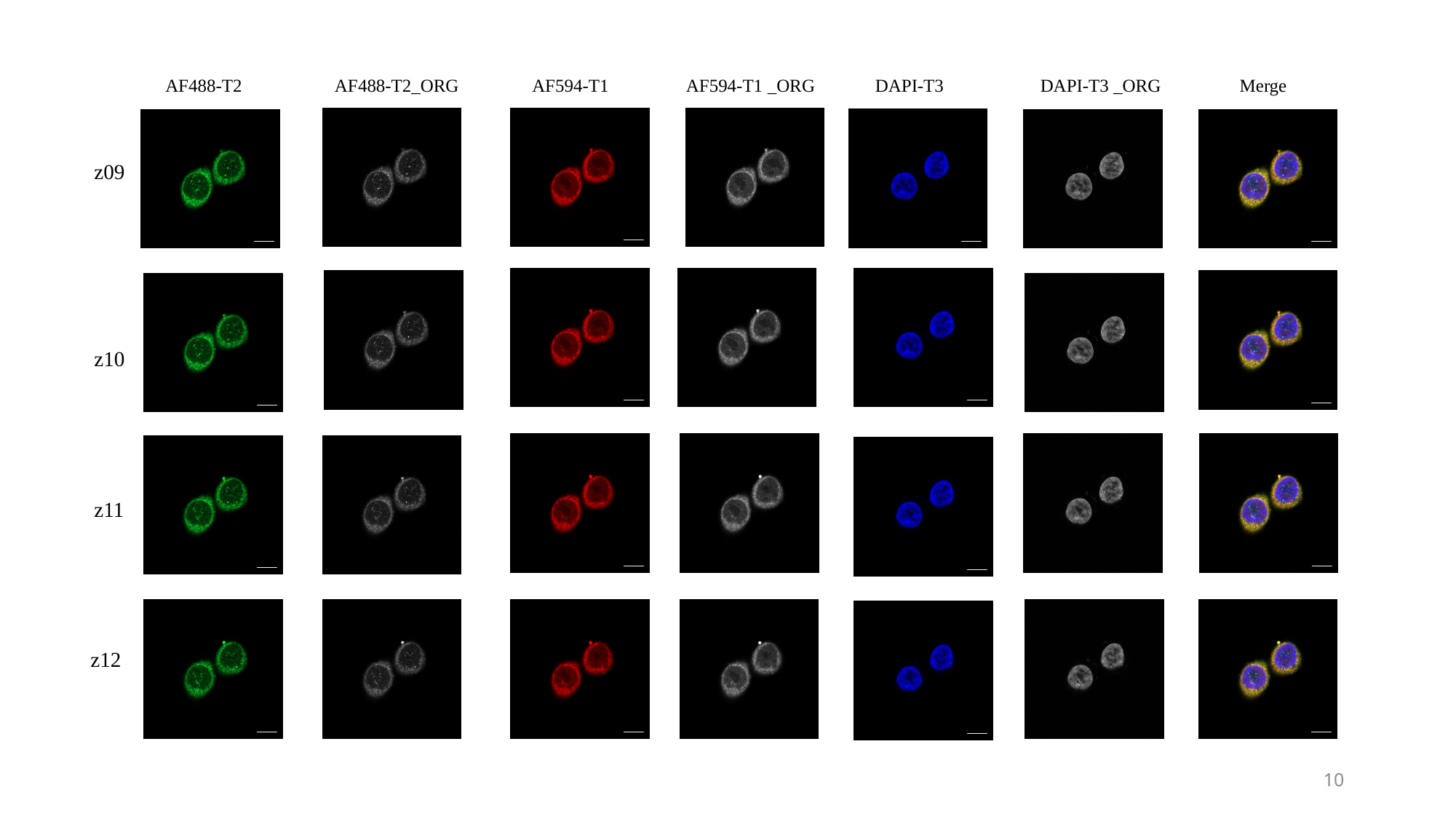

AF488-T2
AF488-T2_ORG
AF594-T1
AF594-T1 _ORG
 DAPI-T3
DAPI-T3 _ORG
Merge
z09
z10
z11
z12
10

## Slide 11
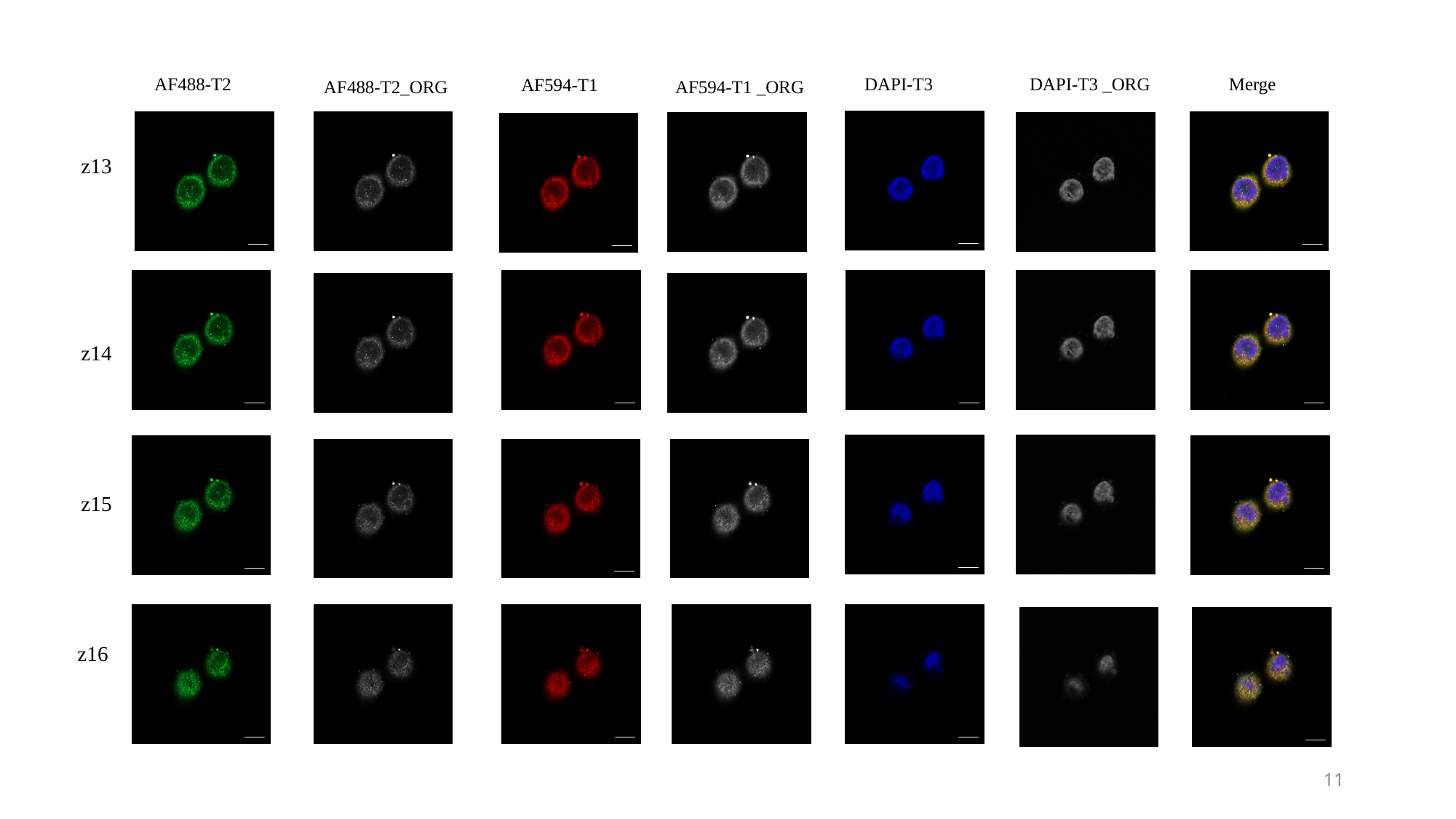

AF488-T2
 DAPI-T3
DAPI-T3 _ORG
Merge
AF594-T1
AF594-T1 _ORG
AF488-T2_ORG
z13
z14
z15
z16
11

## Slide 12
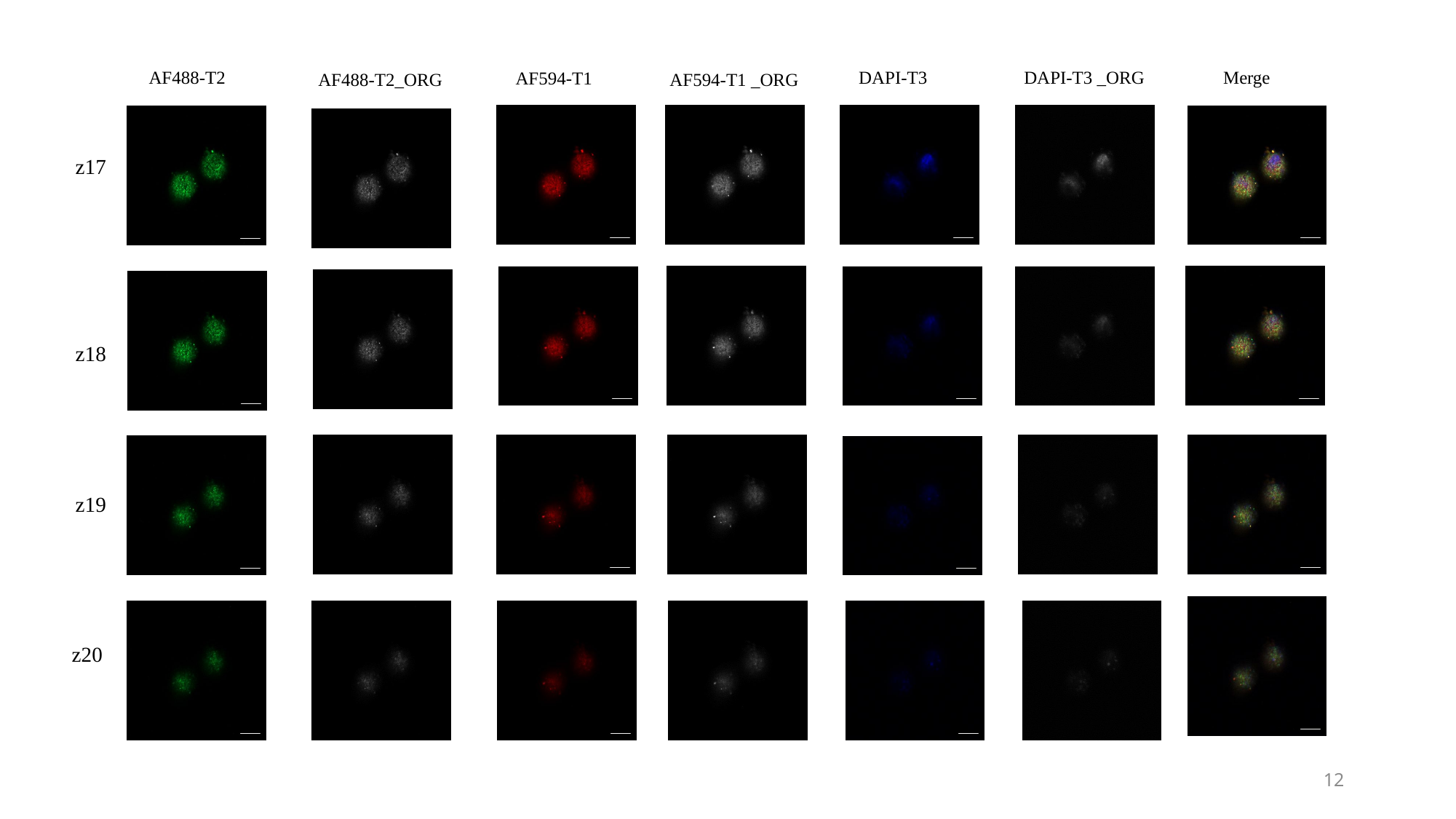

AF488-T2
 DAPI-T3
DAPI-T3 _ORG
Merge
AF594-T1
AF594-T1 _ORG
AF488-T2_ORG
z17
z18
z19
z20
12

## Slide 13
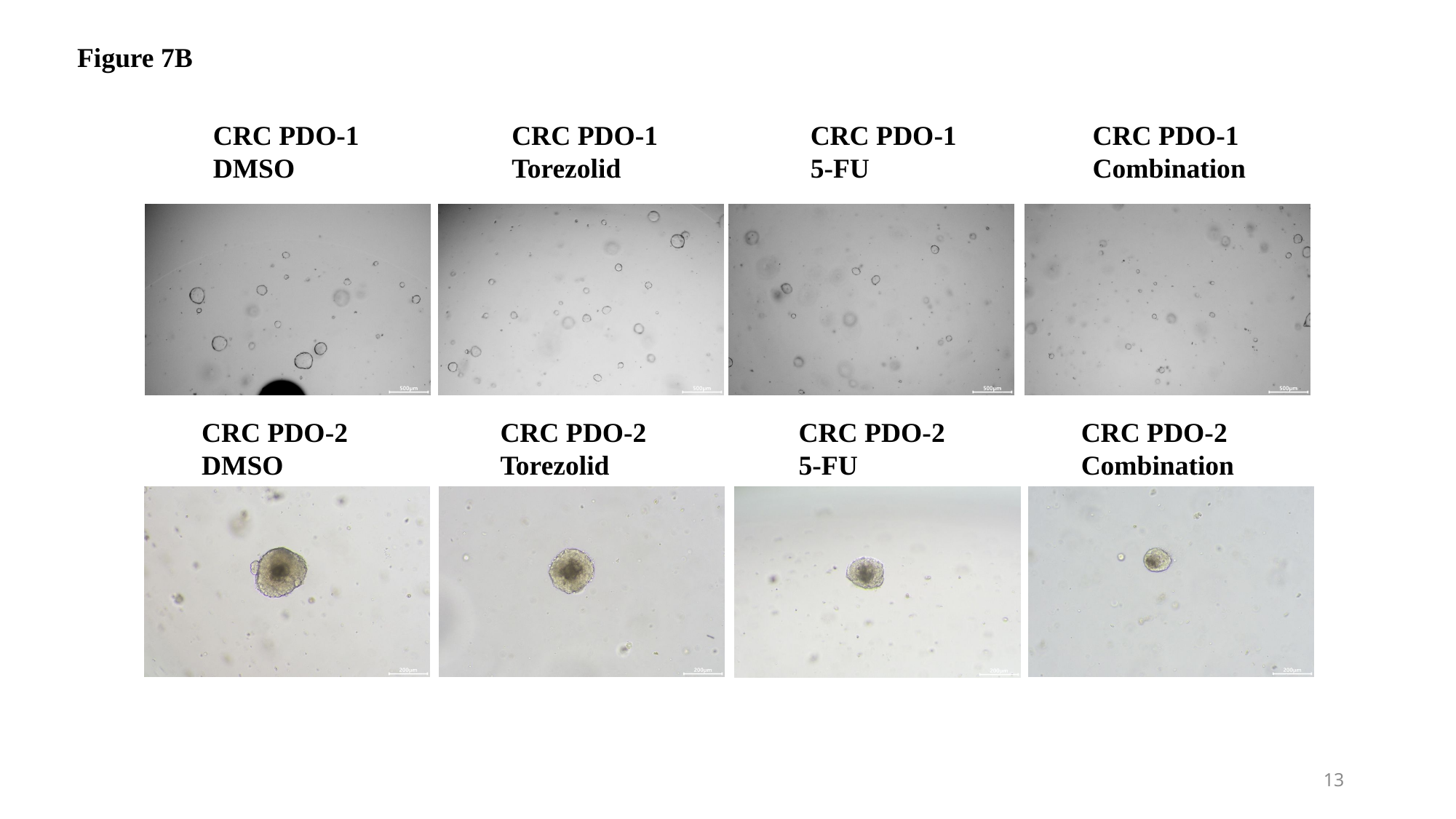

Figure 7B
CRC PDO-1
5-FU
CRC PDO-1
Combination
CRC PDO-1
DMSO
CRC PDO-1
Torezolid
CRC PDO-2
5-FU
CRC PDO-2
Combination
CRC PDO-2
DMSO
CRC PDO-2
Torezolid
13

## Slide 14
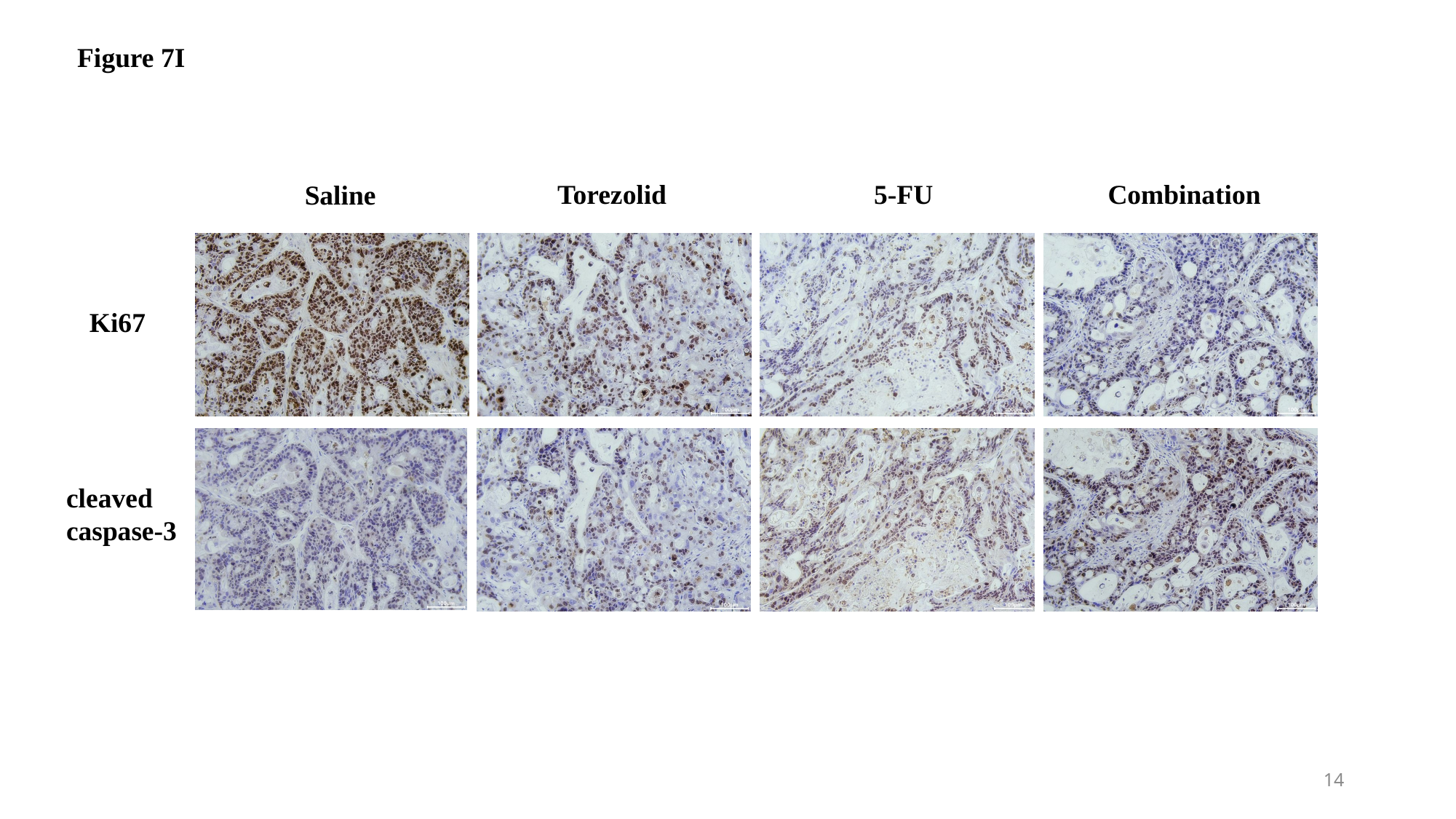

Figure 7I
Torezolid
5-FU
Combination
Saline
Ki67
cleaved
caspase-3
14
