# Supplementary material for: UFL1‐Mediated UFMylation of ENO1 Restrains Aerobic Glycolysis and Colorectal Cancer Progression
Source: Adv Sci (Weinh). 2026 Jul 29:e76875. Online ahead of print. doi: 10.1002/advs.76875 (PMC13418051; doi:10.1002/advs.76875)
Supplement: Supplementary file 2 — Supporting File 2: advs76875‐sup‐0002‐Data.zip. [file ADVS-9999-e76875-s001.zip › advs76875-sup-0002-Data/Data S3 Original scans of blots.docx]

**Data S2. Original scans of blots**


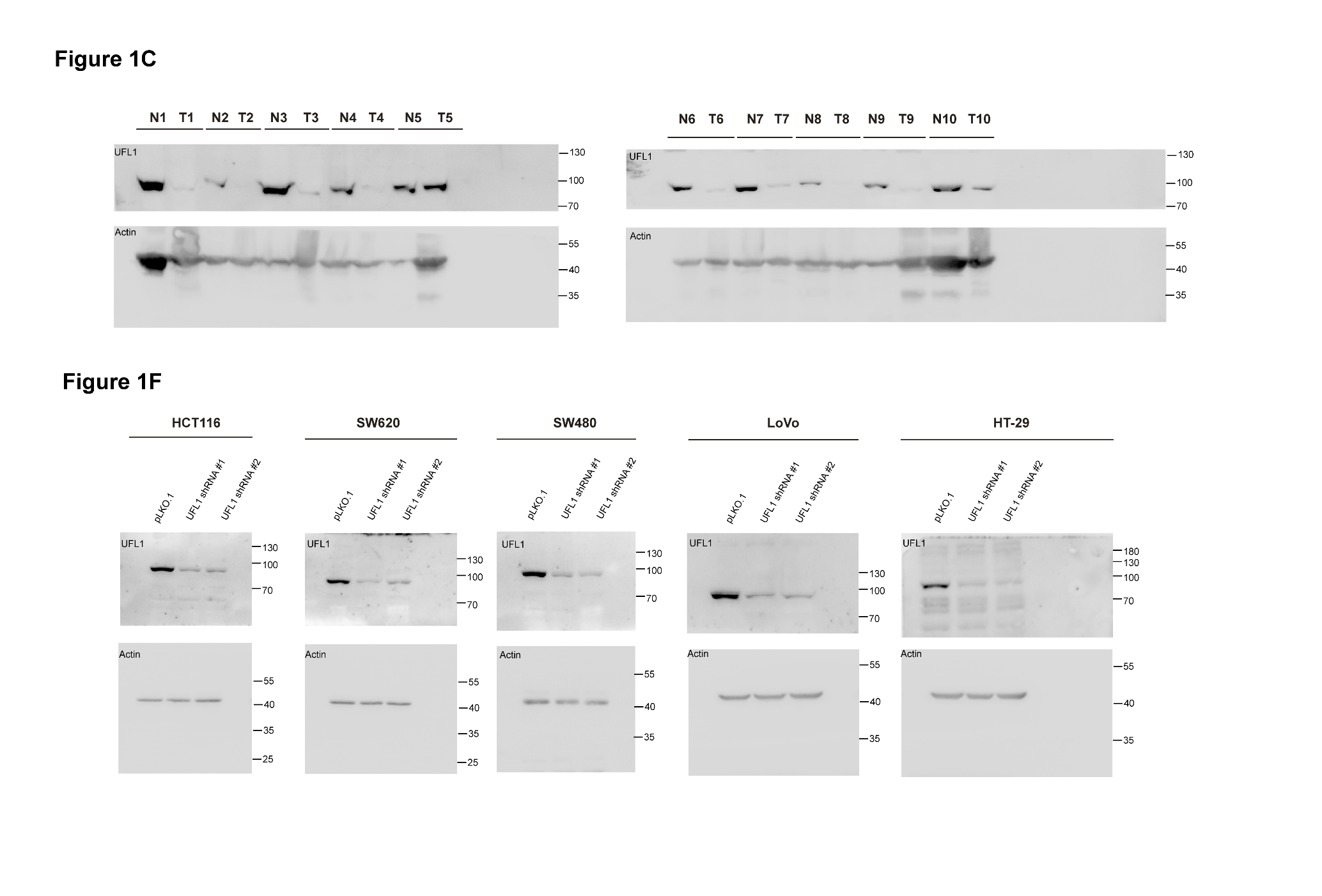


Original scan of the blots presented in the main text. Related to Figure 1.


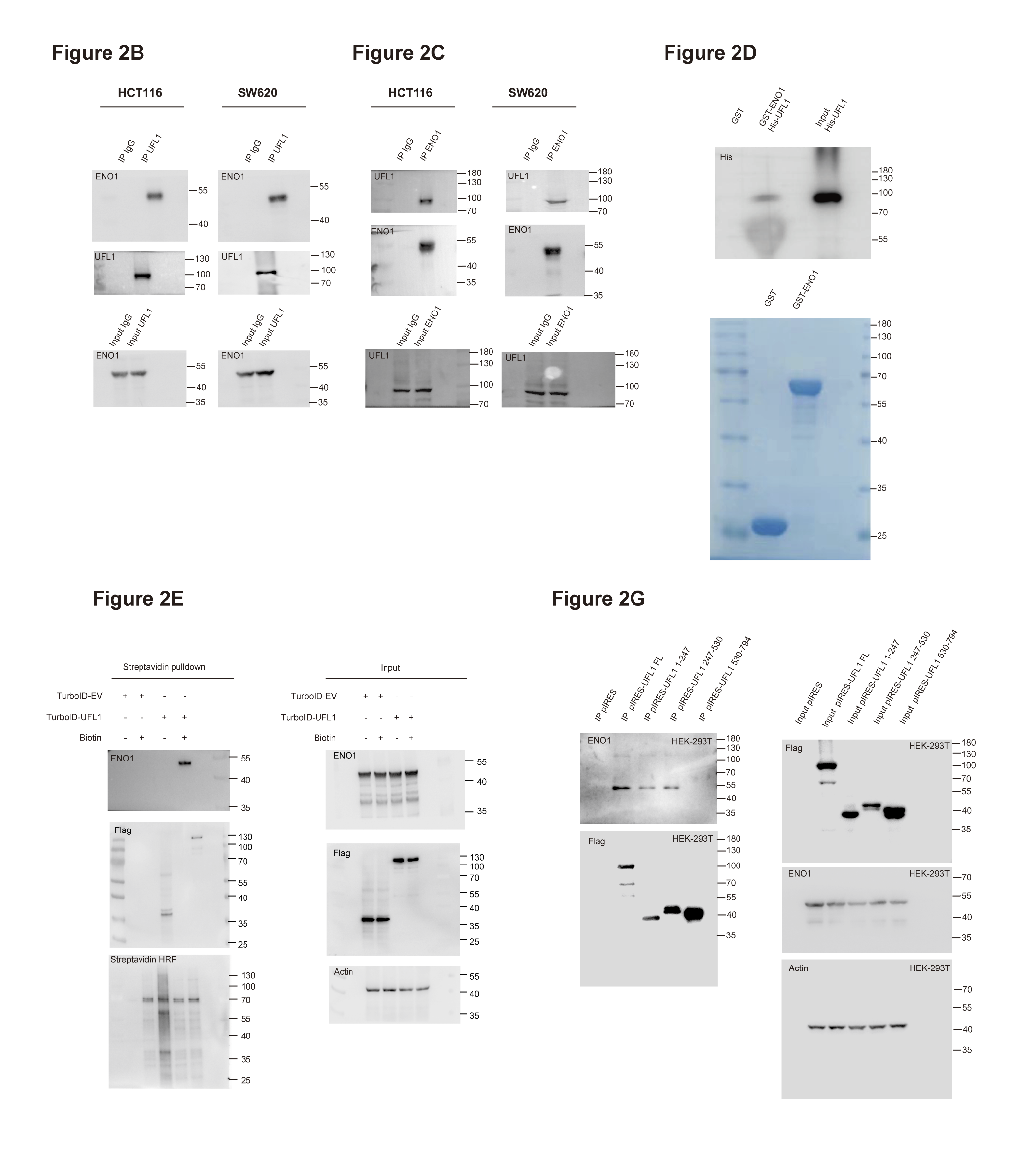


Original scan of the blots presented in the main text. Related to Figure 2.


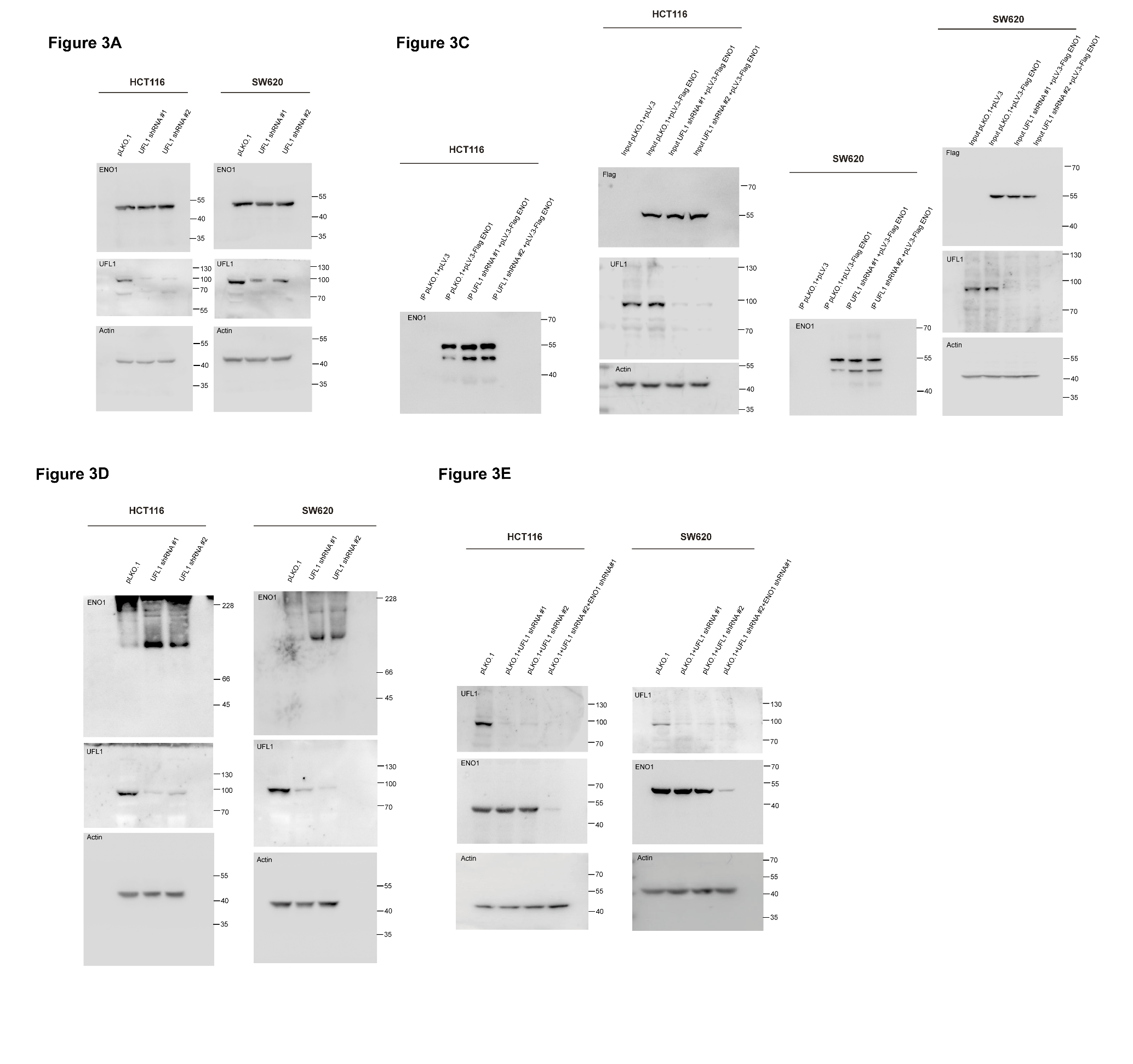


Original scan of the blots presented in the main text. Related to Figure 3.


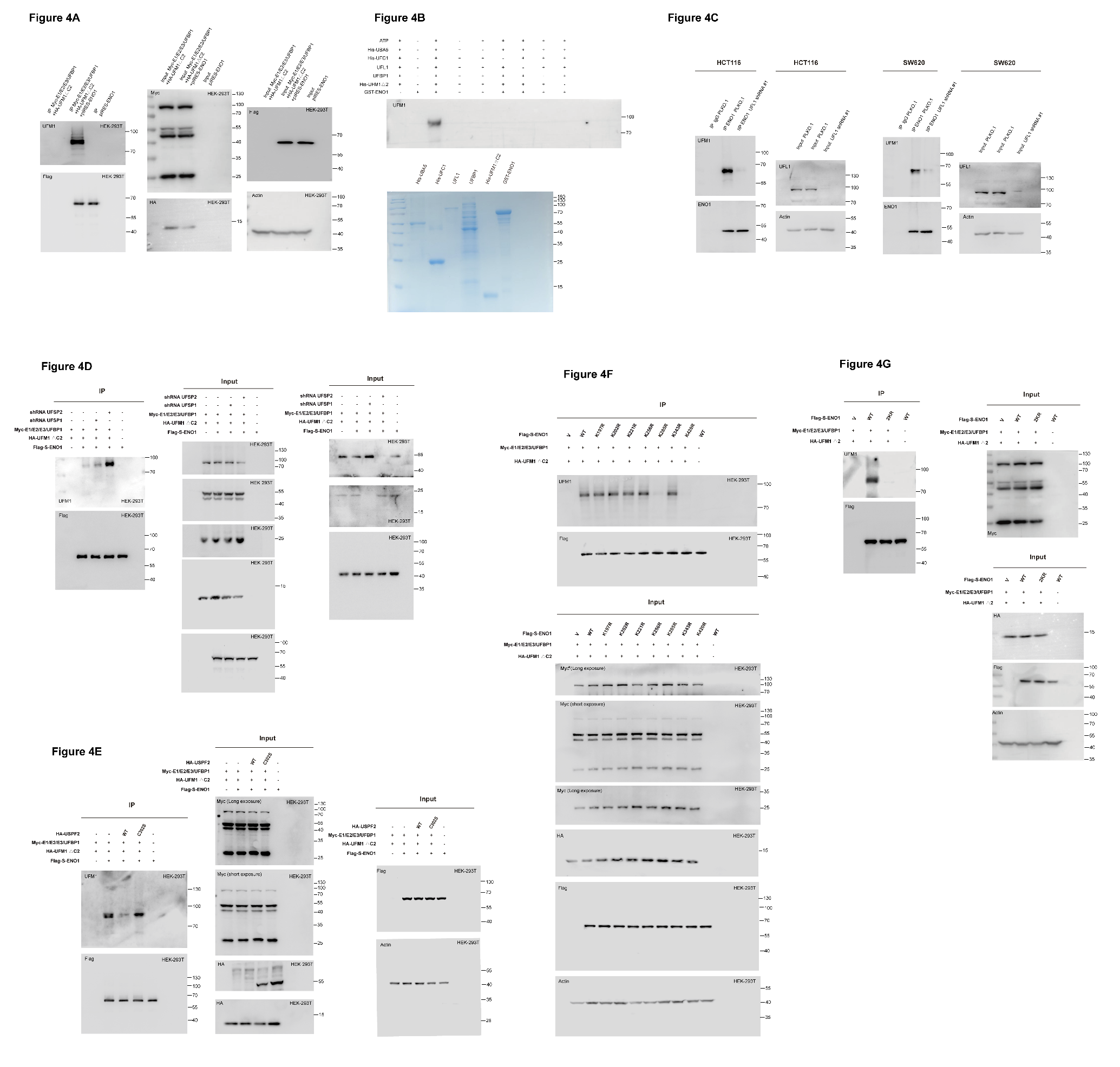


Original scan of the blots presented in the main text. Related to Figure. 4


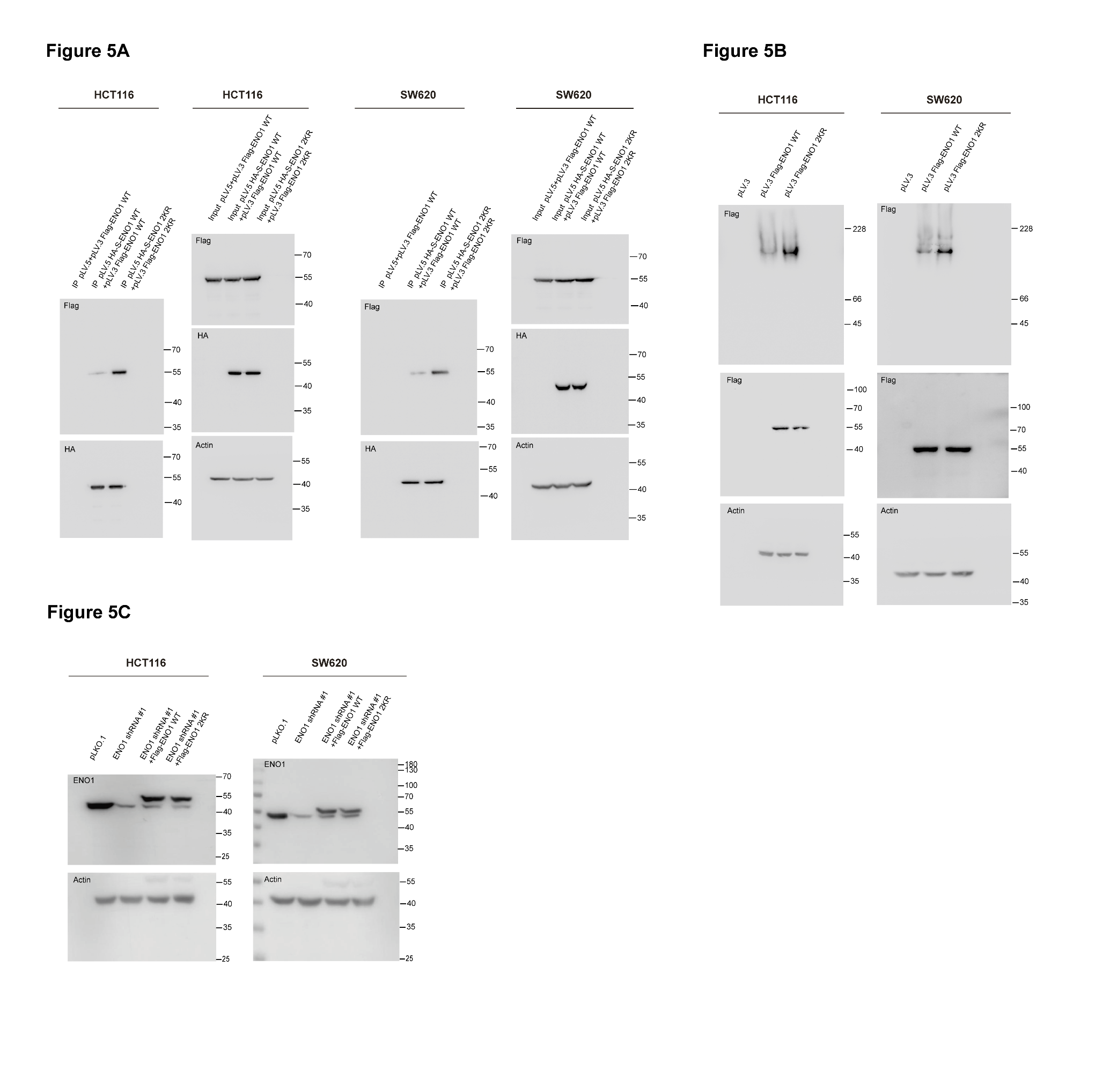


Original scan of the blots presented in the main text. Related to Figure 5.


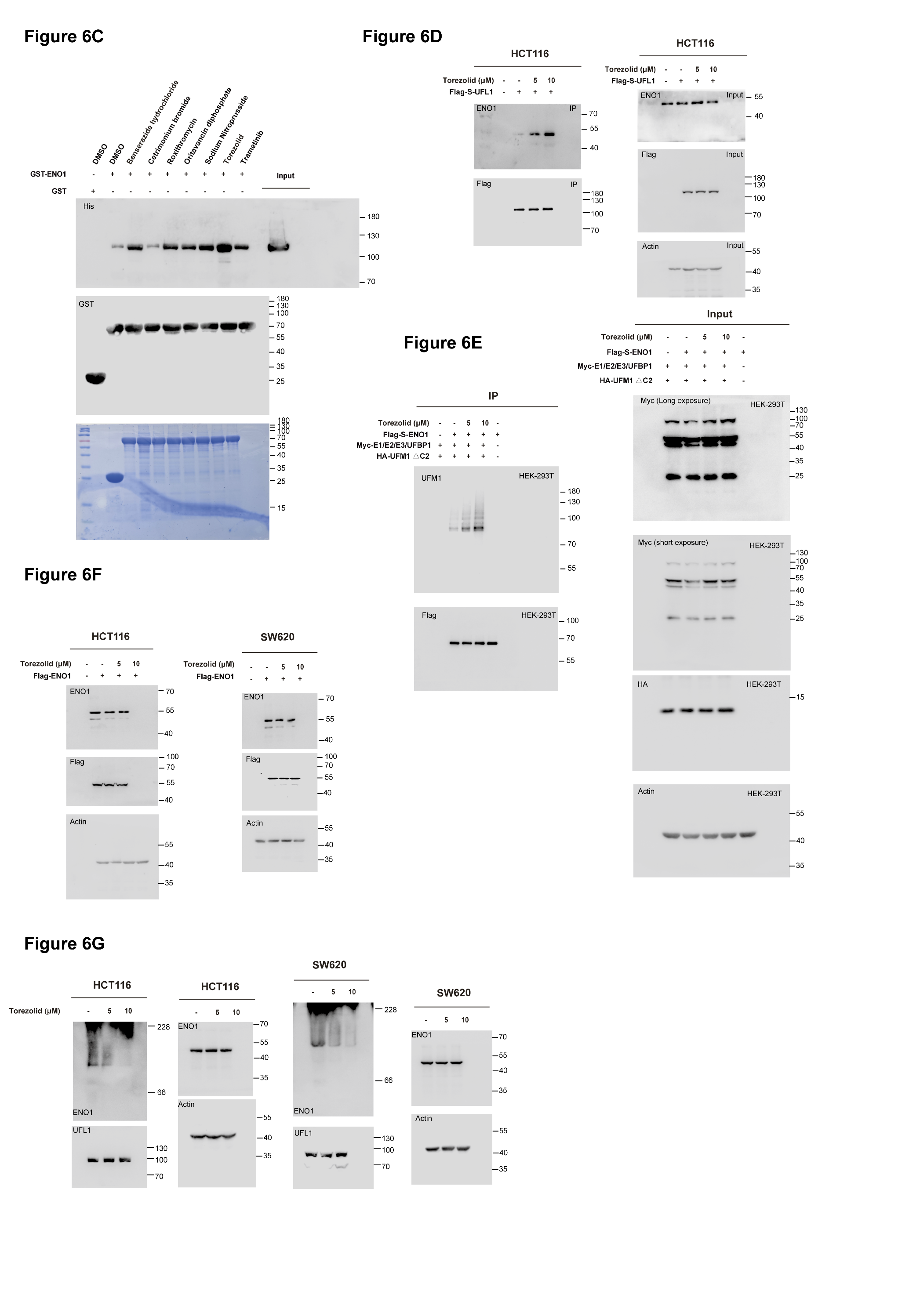


Original scan of the blots presented in the main text. Related to Figure 6.


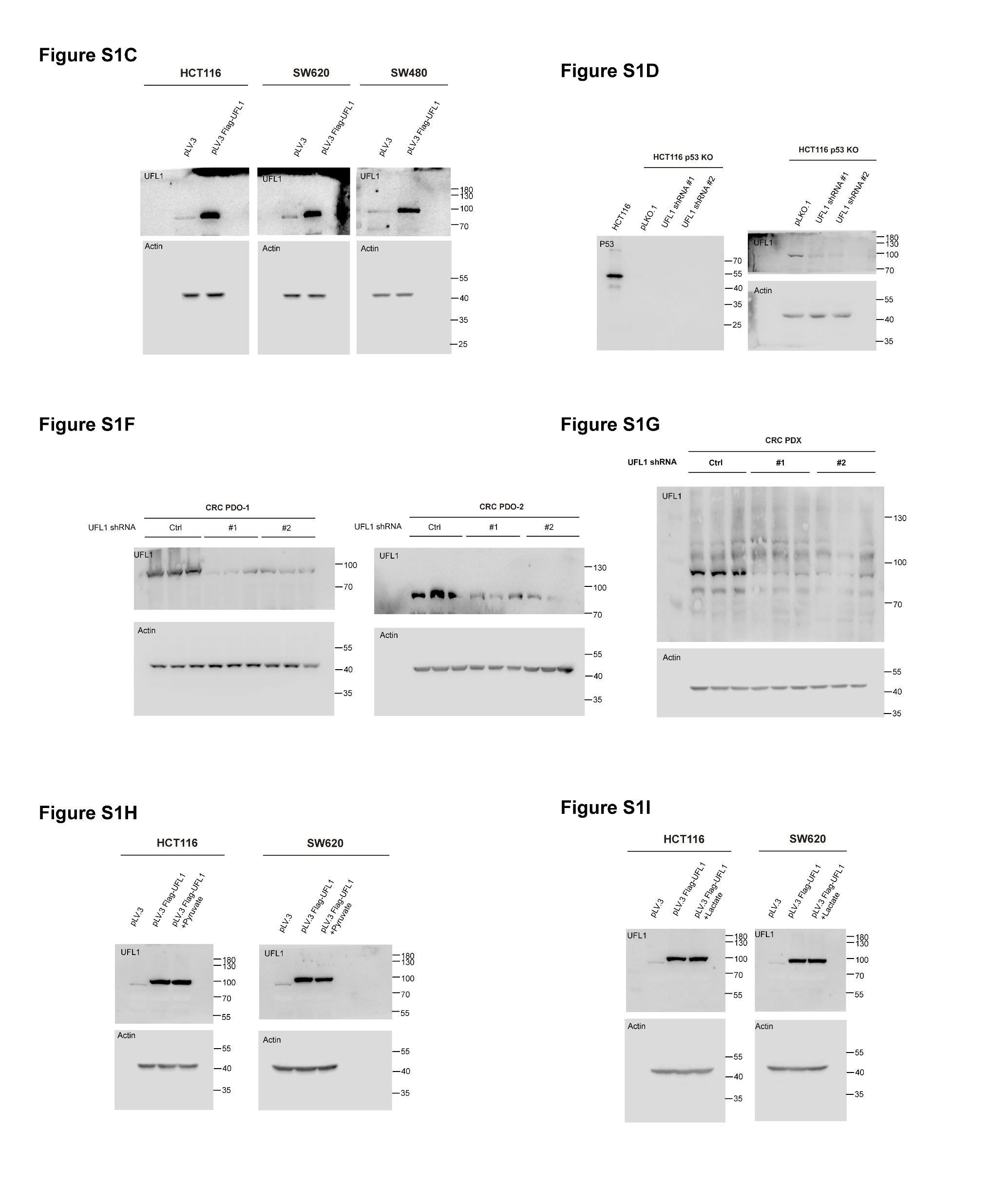


Original scan of the blots presented in the main text. Related to Figure S1.
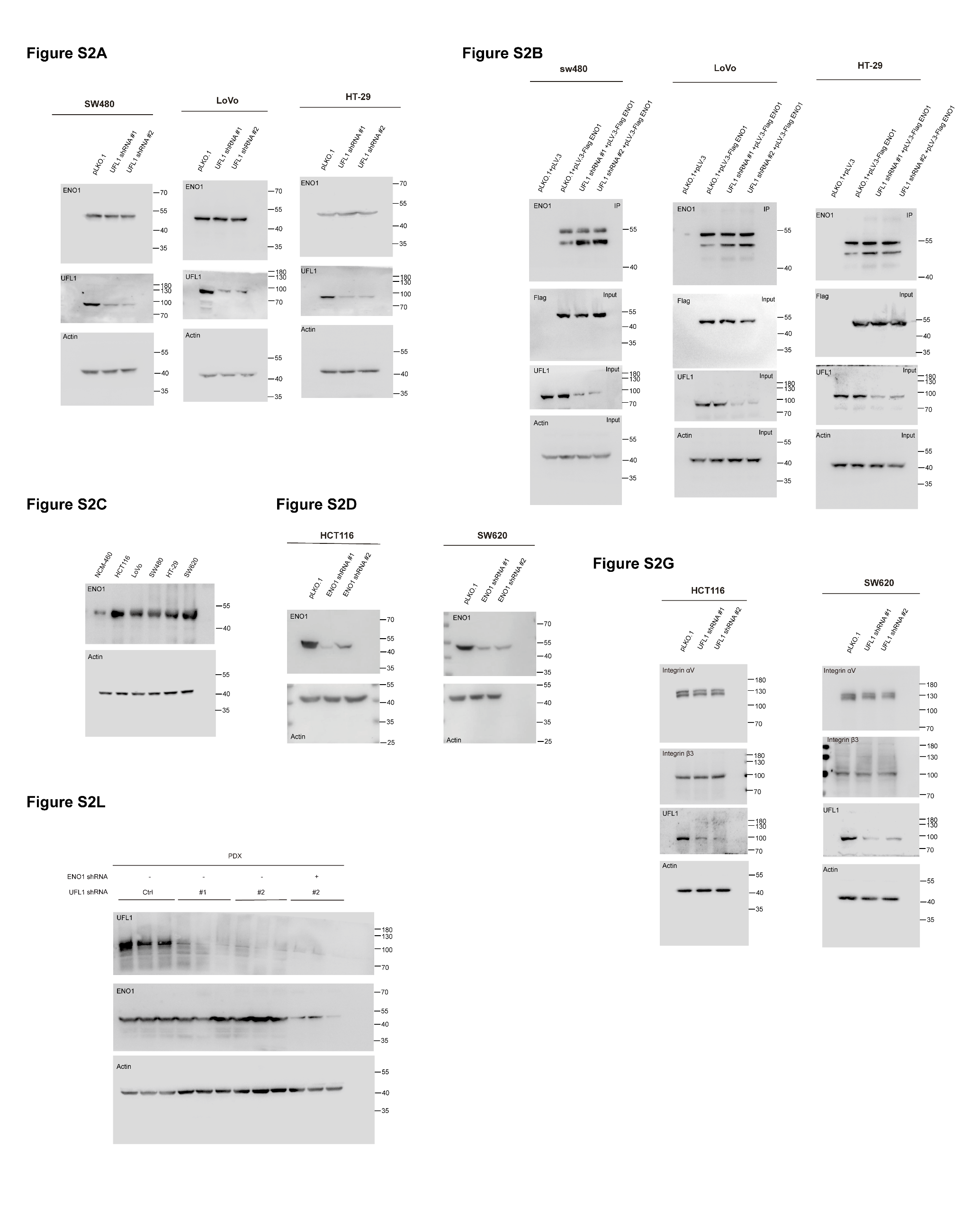


Original scan of the blots presented in the main text. Related to Figure S2.


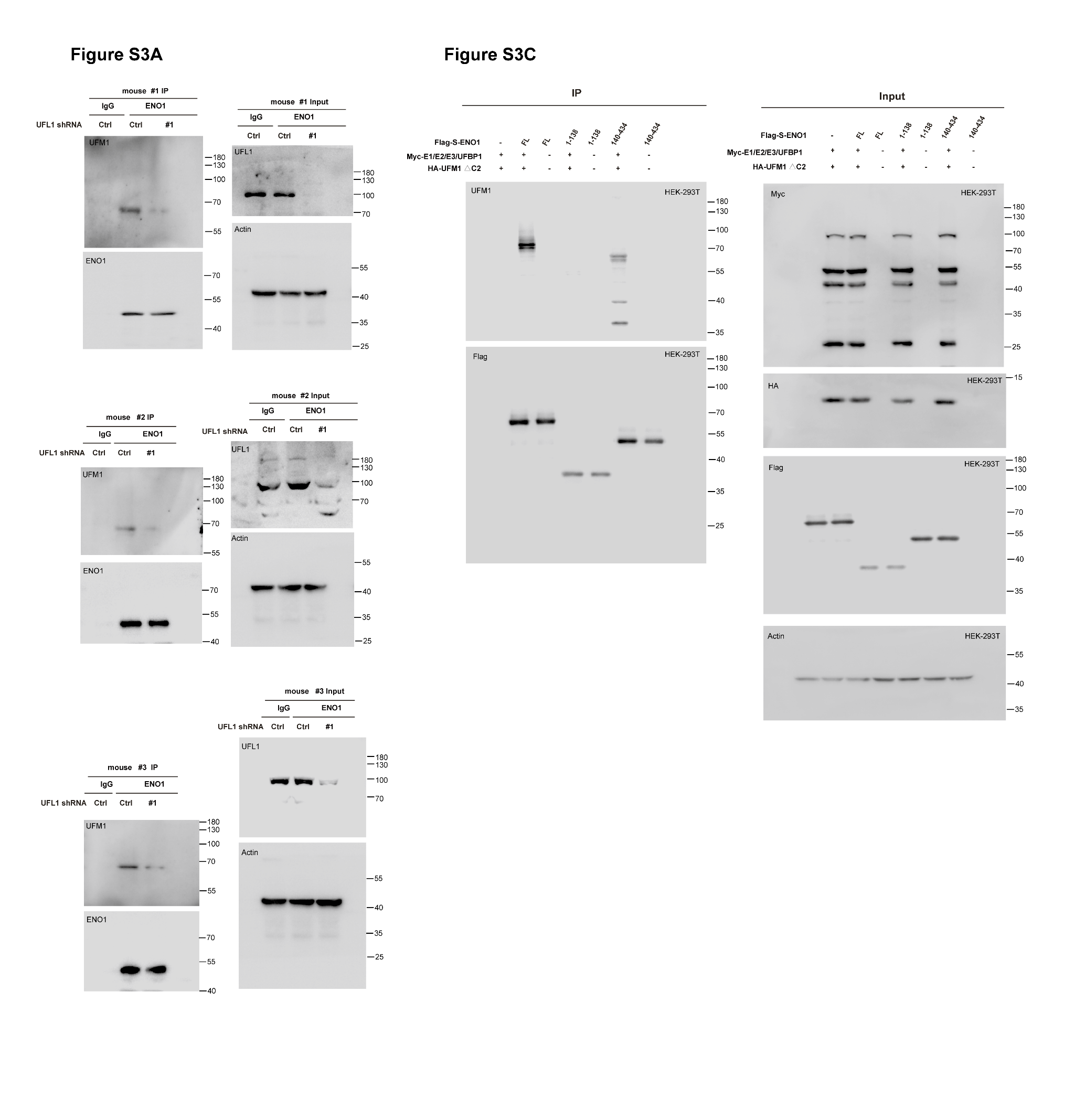


Original scan of the blots presented in the main text. Related to Figure S3.


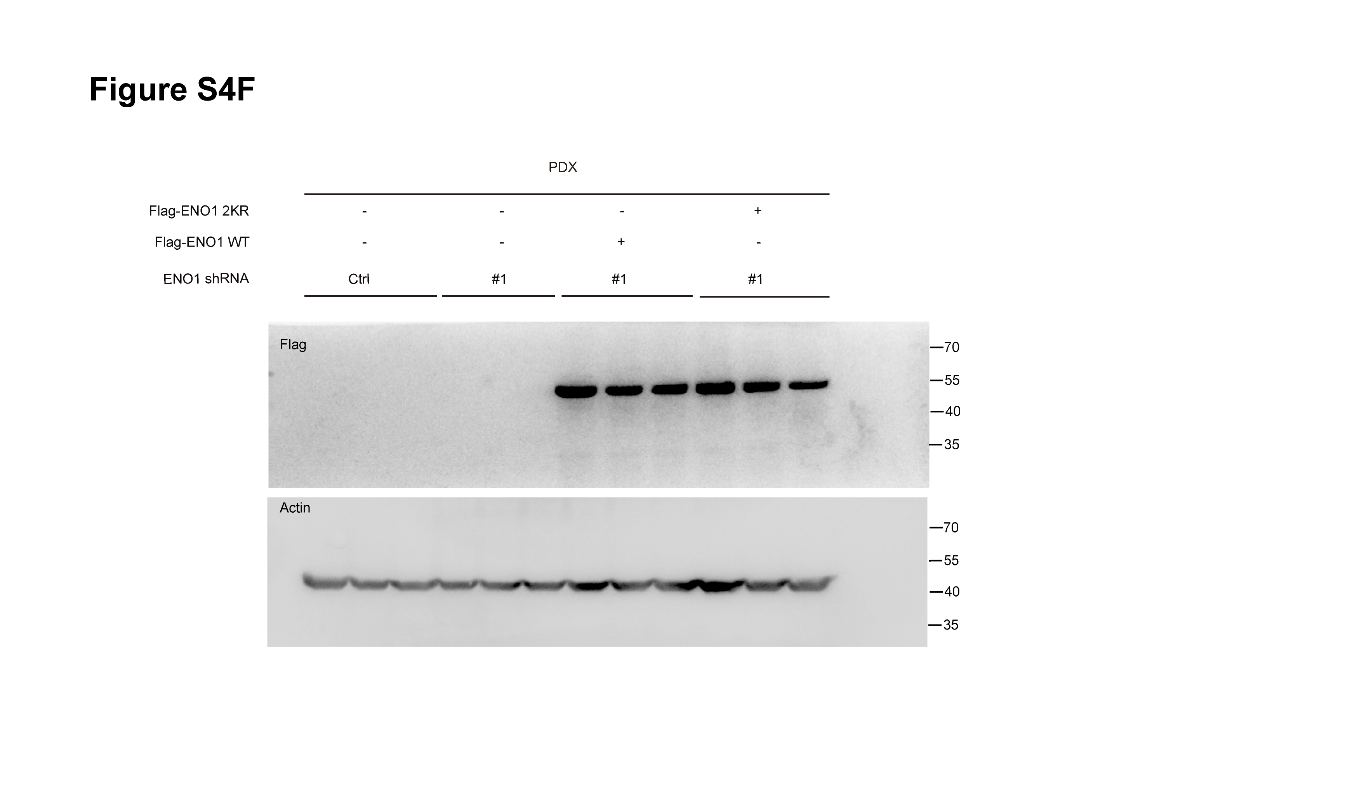


Original scan of the blots presented in the main text. Related to Figure S4.


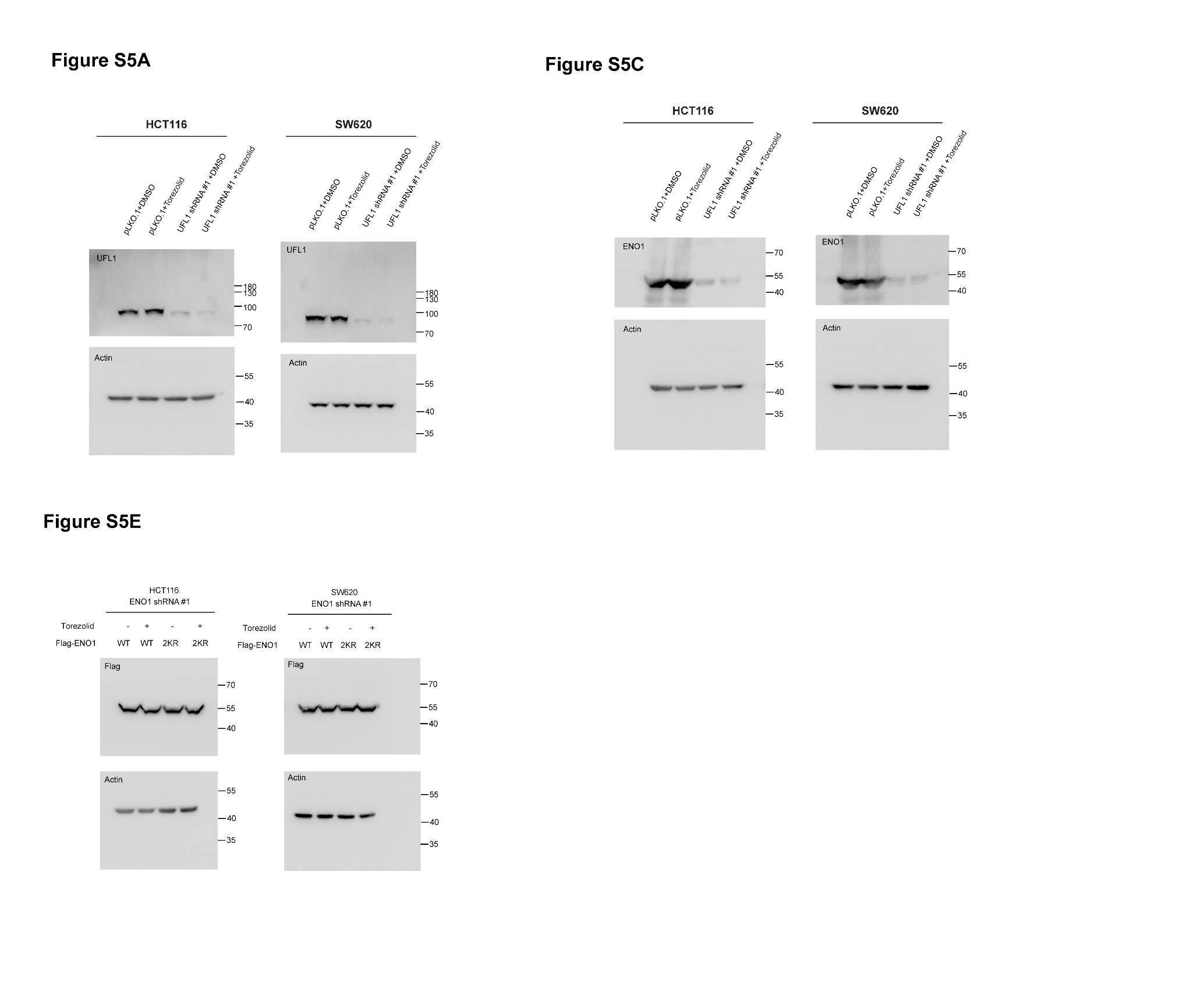


Original scan of the blots presented in the main text. Related to Figure S5.
